# Indigenous Arabs are descendants of the earliest split from ancient Eurasian populations

**DOI:** 10.1101/gr.191478.115

**Published:** 2016-02

**Authors:** Juan L. Rodriguez-Flores, Khalid Fakhro, Francisco Agosto-Perez, Monica D. Ramstetter, Leonardo Arbiza, Thomas L. Vincent, Amal Robay, Joel A. Malek, Karsten Suhre, Lotfi Chouchane, Ramin Badii, Ajayeb Al-Nabet Al-Marri, Charbel Abi Khalil, Mahmoud Zirie, Amin Jayyousi, Jacqueline Salit, Alon Keinan, Andrew G. Clark, Ronald G. Crystal, Jason G. Mezey

**Affiliations:** 1 Department of Genetic Medicine, Weill Cornell Medical College, New York, New York 10065, USA;; 2 Sidra Medical and Research Center, Doha, Qatar;; 3 Department of Genetic Medicine, Weill Cornell Medical College–Qatar, Doha, Qatar;; 4 Department of Biological Statistics and Computational Biology, Cornell University, Ithaca, New York 14850, USA;; 5 Bioinformatics Core, Weill Cornell Medical College–Qatar, Doha, Qatar;; 6 Laboratory Medicine and Pathology, Hamad Medical Corporation, Doha, Qatar;; 7 Department of Medicine, Hamad Medical Corporation, Doha, Qatar

## Abstract

An open question in the history of human migration is the identity of the earliest Eurasian populations that have left contemporary descendants. The Arabian Peninsula was the initial site of the out-of-Africa migrations that occurred between 125,000 and 60,000 yr ago, leading to the hypothesis that the first Eurasian populations were established on the Peninsula and that contemporary indigenous Arabs are direct descendants of these ancient peoples. To assess this hypothesis, we sequenced the entire genomes of 104 unrelated natives of the Arabian Peninsula at high coverage, including 56 of indigenous Arab ancestry. The indigenous Arab genomes defined a cluster distinct from other ancestral groups, and these genomes showed clear hallmarks of an ancient out-of-Africa bottleneck. Similar to other Middle Eastern populations, the indigenous Arabs had higher levels of Neanderthal admixture compared to Africans but had lower levels than Europeans and Asians. These levels of Neanderthal admixture are consistent with an early divergence of Arab ancestors after the out-of-Africa bottleneck but before the major Neanderthal admixture events in Europe and other regions of Eurasia. When compared to worldwide populations sampled in the 1000 Genomes Project, although the indigenous Arabs had a signal of admixture with Europeans, they clustered in a basal, outgroup position to all 1000 Genomes non-Africans when considering pairwise similarity across the entire genome. These results place indigenous Arabs as the most distant relatives of all other contemporary non-Africans and identify these people as direct descendants of the first Eurasian populations established by the out-of-Africa migrations.

All humans can trace their ancestry back to Africa (Cann et al. 1987), where the ancestors of anatomically modern humans first diverged from primates (Patterson et al. 2006), and then from archaic humans (Prüfer et al. 2014). Humans began leaving Africa through a number of coastal routes, where estimates suggest these “out-of-Africa” migrations reached the Arabian Peninsula as early as 125,000 yr ago (Armitage et al. 2011) and as late as 60,000 yr ago (Henn et al. 2012). After entering the Arabian Peninsula, human ancestors entered South Asia and spread to Australia (Rasmussen et al. 2011), Europe, and eventually, the Americas. The individuals in these migrations were the most direct ancestors of ancient non-African peoples, and they established the contemporary non-African populations recognized today (Cavalli-Sforza and Feldman 2003).

The relationship between contemporary Arab populations and these ancient human migrations is an open question (Lazaridis et al. 2014; Shriner et al. 2014). Given that the Arabian Peninsula was an initial site of egress from Africa, one hypothesis is that the original out-of-Africa migrations established ancient populations on the peninsula that were direct ancestors of contemporary Arab populations (Lazaridis et al. 2014). These people would therefore be direct descendants of the earliest split in the lineages that established Eurasian and other contemporary non-African populations (Armitage et al. 2011; Rasmussen et al. 2011; Henn et al. 2012; Lazaridis et al. 2014; Shriner et al. 2014). If this hypothesis is correct, we would expect that there are contemporary, indigenous Arabs who are the most distant relatives of other Eurasians. To assess this hypothesis, we carried out deep-coverage genome sequencing of 104 unrelated natives of the Arabian Peninsula who are citizens of the nation of Qatar (Supplemental Fig. 1), including 56 of indigenous Bedouin ancestry who are the best representatives of autochthonous Arabs, and compared these genomes to contemporary genomes of Africa, Asia, Europe, and the Americas (The 1000 Genomes Project Consortium 2012; Lazaridis et al. 2014).

## Results

### Population structure of the Arabian Peninsula

Previous analyses of the populations of the Arabian Peninsula (Hunter-Zinck et al. 2010; Alsmadi et al. 2013) have found three distinct clusters that reflect primary ancestry: Q1 (Bedouin); Q2 (Persian-South Asian); and Q3 (African) (Omberg et al. 2012). By assessment of medical records and ancestry-informative SNP genotyping (Supplemental Fig. 2), a sample of 108 purportedly unrelated individuals was selected for sequencing, including 60 Q1 (Bedouin), 20 Q2 (Persian-South Asian), and 20 Q3 (African), as well as 8 Q0 (Subpopulation Unassigned) that could not be cleanly placed in one of these three groups (Supplemental Table I). Each of these genomes was sequenced to a median depth of 37× (minimum 30×) by Illumina technology, identifying a total of 23,784,210 SNPs (see Methods, Supplemental Table II).

To confirm that none of the 108 individuals were closely related, we used KING-robust (Manichaikul et al. 2010) and PREST-plus (McPeek and Sun 2000) to estimate family relationships based on a set of 1,407,483 SNPs after pruning of the full set of 22,958,844 autosomal SNPs in Qatar (see Methods). Both analyses identified five pairs of related individuals greater than third-degree that were subsequently confirmed by investigative reassessment of medical records (Supplemental Table III; Supplemental Fig. 3). Three of the pairs form a trio; hence, two individuals from the trio were removed, and one individual from each of the two remaining pairs was removed, such that the remaining 104 individuals analyzed further included 8 Q0 (Subpopulation Unassigned) and 96 Q1, Q2, or Q3 Qatari: 56 Q1 (Bedouin), 20 Q2 (Persian-South Asian), and 20 Q3 (African).

An analysis of inbreeding for these remaining individuals showed the Q1 (Bedouin) to have a more positive inbreeding coefficient than most of the non-admixed 1000 Genomes (The 1000 Genomes Project Consortium 2012) populations (Supplemental Table IV; Supplemental Fig. 4), consistent with the known inbreeding of this group (Hunter-Zinck et al. 2010; Omberg et al. 2012); although we also found the Q1 (Bedouin) to be less inbred than many small and/or isolated populations worldwide represented in the Human Origins samples (Lazaridis et al. 2014) (Supplemental Table V; Supplemental Table VI; Supplemental Fig. 4). The Q2 (Persian-South Asian) had a positive, but slightly lower, inbreeding coefficient than the Q1 (Bedouin). In contrast, the Q3 (African) had a non-negative coefficient that reflects known admixture with African populations (Hunter-Zinck et al. 2010; Omberg et al. 2012).

**Figure 1.**
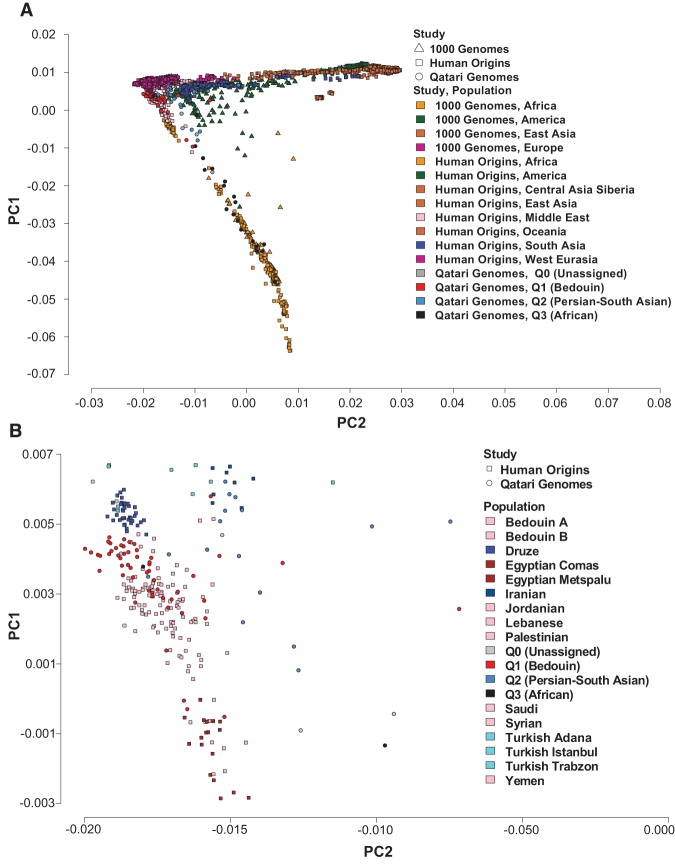
Principal component analysis (PCA) (Price et al. 2006) of the 104 Qatari genomes (circle), 1000 Genomes (triangle), and Human Origins (square) study samples. Shown are individuals plotted on principal components PC1 and PC2, with genomes color-coded by study and population, with the Q0 (Subpopulation Unassigned) in gray, Q1 (Bedouin) in red, Q2 (Persian-South Asian) in azure, and Q3 (African) in black. (*A*) Plot of all populations, defined by study and by population, in which all populations from the same region and study are grouped and color-coded together (1000 Genomes: Africa, America, East Asia, and Europe; Human Origins: Africa, America, Central Asia/Siberia, East Asia, Middle East, Oceania, South Asia, and West Eurasia). (*B*) Plot of Middle Eastern subpopulations from Human Origins that cluster near Q1 (Bedouin) and Q2 (Persian-South Asian).

We confirmed the primary ancestry classifications of the 104 Qataris by principal component analysis (Price et al. 2006). We combined the 104 Qataris, the Human Origins populations (Lazaridis et al. 2014), and 1000 Genomes populations (The 1000 Genomes Project Consortium 2012) (excluding individuals already in Human Origins), and performed principal component analysis on a set of 197,714 linkage disequilibrium pruned autosome SNPs (Fig. 1A; Supplemental Fig. 5A). We also confirmed these clusterings just with the 104 Qataris and 1000 Genomes samples based on the same set of autosomal SNPs (Supplemental Fig. 5B). These analyses reproduced the population clustering observed previously (Hunter-Zinck et al. 2010; Omberg et al. 2012), with the Q1 (Bedouin) closest to Europeans, the Q2 (Persian-South Asian) between Q1 (Bedouin) and Asians, and the Q3 (African) closest to African populations. A plot of just the Middle Eastern populations on the principal components also showed clustering as expected, with the Q1 (Bedouin) clustering with previously sampled Bedouins and Arabs, Q2 (Persian-South Asians) with Iranians, and Q3 (African) outside of the Middle Eastern cluster (data not shown) (Fig. 1B).

### Y Chromosome and mitochondrial DNA haplogroups

**Figure 2.**
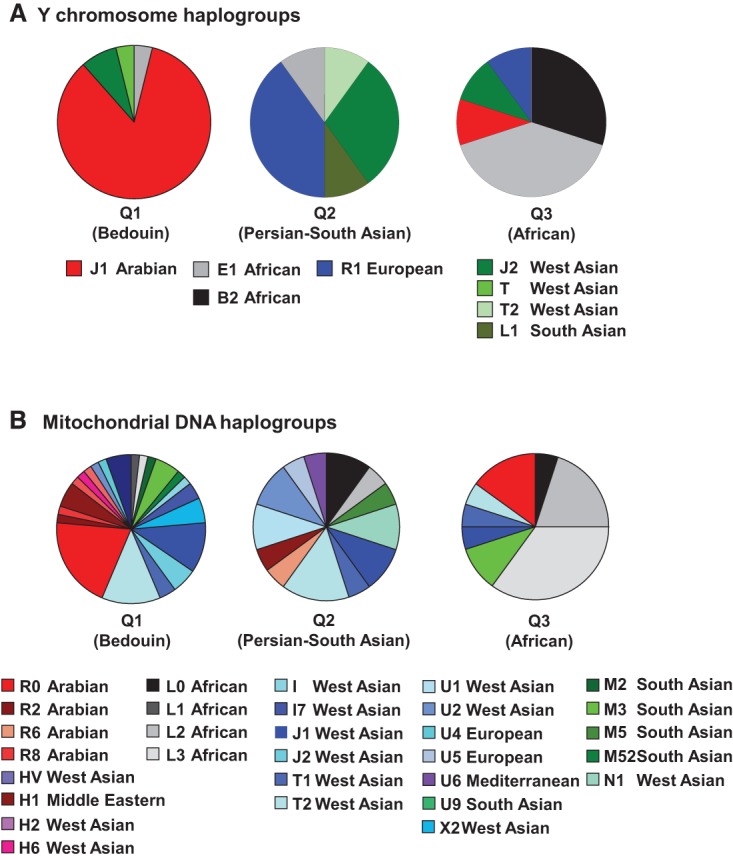
Y Chromosome (Chr Y) and mitochondrial DNA (mtDNA) haplogroup assignments. The Chr Y and mtDNA haplogroups were determined for Q1 (Bedouin), Q2 (Persian-South Asian), and Q3 (African). (*A*) Pie charts of the haplogroup frequencies for Chr Y. (*B*) Pie charts of the haplogroup frequencies for mtDNA.

We next analyzed the Y Chromosome (Chr Y) and mitochondrial DNA (mtDNA) to assess the degree to which the Q1 (Bedouin), Q2 (Persian-South Asian), or Q3 (African) Qatari ancestry groups represent distinct subpopulations (Fig. 2). The Chr Y haplogroups showed almost no overlap between the Q1 (Bedouin) Qataris and Q2 (Persian-South Asian) Qataris, in which an Analysis of Molecular Variance (AMOVA) was highly significant (*P* < 0.018) (Supplemental Table VII). The Arab haplogroup J1 was the dominant haplogroup in the Q1 (Bedouin) Qataris, but this haplogroup was not represented at all among the Q2 (Persian-South Asian) Qataris (Fig. 2A). This confirmed that these are genetically well-defined subpopulations that are relatively isolated from one another (Omberg et al. 2012). There was also a strong partitioning of the Chr Y haplogroups when considering the Q3 (African) Qataris, both when considering Q1 (Bedouin) versus Q3 (African) (AMOVA *P* < 1 × 10^−5^) and Q2 (Persian-South Asian) versus Q3 (African) (AMOVA *P* < 0.028). The Q3 (African) had largely African haplogroups, a result consistent with the known recent African admixture of this subpopulation (Omberg et al. 2012).

The mtDNA haplogroups were less partitioned among the Qataris, although they still showed significant partitioning between each pair of subpopulations (AMOVA Q1 versus Q2 *P* < 0.035, Q1 versus Q3 *P* < 1 × 10^−5^, Q2 versus Q3 *P* < 0.017) and among all three considered simultaneously (AMOVA *P* < 1 × 10^−5^) (Supplemental Table VII). The mtDNA haplogroups also included more worldwide geographic diversity overall, indicating a different male versus female pattern of intermarriage among these subpopulations (Sandridge et al. 2010). Together the Chr Y and mtDNA haplogroups indicate that the Q1 (Bedouin), Q2 (Persian-South Asian), and Q3 (African) ancestry groups represent genetic subpopulations that not only reflect known migration history (Hunter-Zinck et al. 2010; Omberg et al. 2012) but that also represent units defined by a patrilocal society with strong historical barriers to intermarriage (Esposito 2001; Cavalli-Sforza and Feldman 2003), in which gene flow has been dominated by female movement (i.e., admixture occurring through females marrying into the relatively isolated subpopulations), as well as female influxes from other geographic areas.

### X-linked and autosomal diversity

To further analyze the relative male and female contributions to the genetics of the Qatari Q1 (Bedouin), Q2 (Persian-South Asian), and Q3 (African) subpopulations, we analyzed genome-wide ratios of X-linked and autosomal (X/A) diversity and X/A diversity ratios for genome intervals >0.18 cM from genes (Supplemental Table VIII; Supplemental Fig. 6). For both of these ratios, the Q1 (Bedouin) and Q2 (Persian-South Asian) were lower than for African populations but were higher than for Europeans and Asians. This points to a higher effective population size of females in the Q1 (Bedouin) and Q2 (Persian-South Asian), possibly a consequence of the out-of-Africa migrations, which were believed to be biased toward migration of males over females (Gottipati et al. 2011; Arbiza et al. 2014). The Q3 (African) Qataris had X/A diversity ratios that were higher, even when compared to African populations. This may be driven by a smaller male effective population size; a possible consequence of a polygamous culture and the ancestry of the Q3 (African) subpopulation that was a result of the historical slave trade into the region from Africa (Omberg et al. 2012).

We also analyzed the relative ratios of X-linked and autosomal (X/A) diversity in nongenic regions of the female Q1 (Bedouin), Q2 (Persian-South Asian), and Q3 (African) genomes compared to females in African populations of the 1000 Genomes Project (Supplemental Table IX). The relative X/A ratios of both the Q1 (Bedouin) and Q2 (Persian-South Asian) to African populations were slightly higher than when comparing European to African populations (Gottipati et al. 2011; Arbiza et al. 2014). This could indicate a slightly less extreme set of bottleneck events encountered since the out-of-Africa migrations by the direct ancestors of the Q1 (Bedouin) and Q2 (Persian-South Asian) compared to the bottlenecks encountered by the direct ancestors of Europeans. The relative X/A diversity ratios of Q3 (African) to African populations were closer to one, consistent with the known African admixture of this subpopulation (Omberg et al. 2012).

### Pairwise sequential Markov coalescent analysis

**Figure 3.**
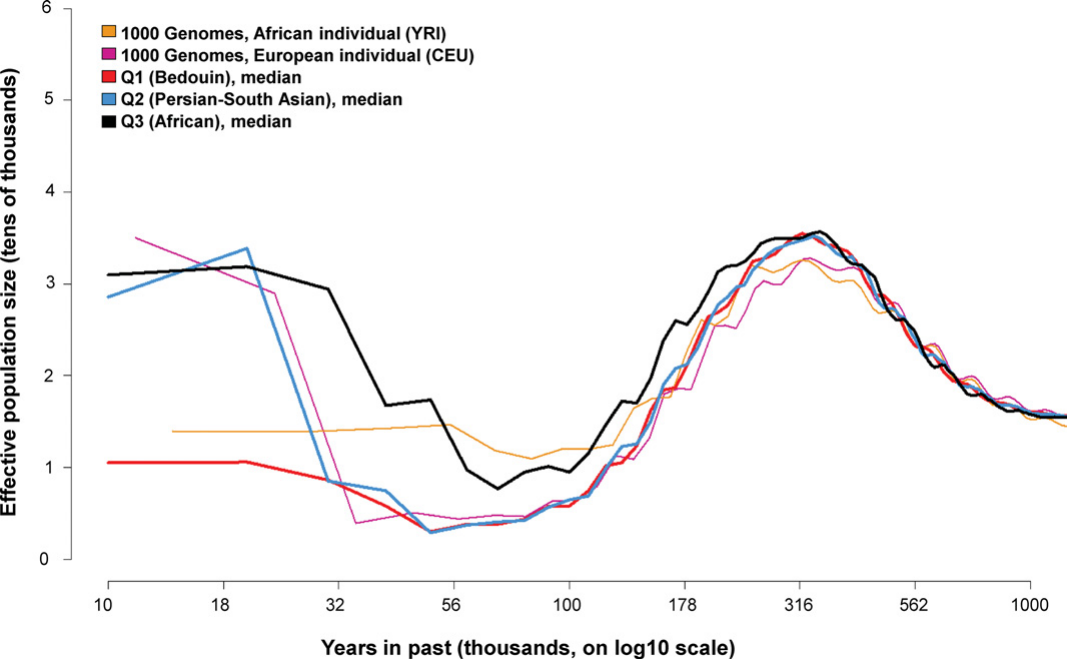
Ancient bottlenecks in the 96 Q1 (Bedouin), Q2 (Persian-South Asian), or Q3 (African) Qatari genomes (56 Q1, 20 Q2, 20 Q3) determined by pairwise sequential Markov coalescent analysis (Li and Durbin 2011). Shown is the plot of the median effective population size (*y*-axis) across individuals in a subpopulation versus years in the past (log scale *x*-axis) for the samples in the three major Qatari subpopulations: Q1 (Bedouin) in red, Q2 (Persian-South Asian) in azure, Q3 (African) in black. A single individual of European ancestry (NA12879, violet) and a single individual of African ancestry (NA19239, orange) from the 1000 Genomes Project deep-coverage pilot (The 1000 Genomes Project Consortium 2010) are shown for comparison.

We next analyzed the full complement of autosomal polymorphisms for signals of ancient bottlenecks by applying the pairwise sequential Markov coalescent (PSMC) (Fig. 3; Li and Durbin 2011). This analysis showed that the Q1 (Bedouin) and Q2 (Persian-South Asian) had clear hallmarks of a bottleneck event, with effective population size hitting a trough in the range of 100,000 to 30,000 yr ago with a minimum at ∼60,000 yr ago. This same pattern is observed for a European individual from the 1000 Genomes Project and is consistent with what has been observed in other non-African human genomes using the pairwise sequential Markov coalescent, as well as related methods (Gronau et al. 2011; Fu et al. 2014; Schiffels and Durbin 2014). These data, therefore, point to the ancestors of Q1 (Bedouin) and Q2 (Persian-South Asian) as having migrated out of Africa at the same time as the ancestors of other non-African populations (Henn et al. 2012). Although PSMC estimates in the more recent past tend to have larger confidence intervals (Li and Durbin 2011), the Q1 (Bedouin) do appear to have a lower population size than the Q2 (Persian-South Asian) in the region <30,000 yr ago, consistent with high levels of inbreeding in the Q1 (Bedouin) (Hunter-Zinck et al. 2010; Sandridge et al. 2010; Mezzavilla et al. 2015). For the Q3 (African), the median effective population size was more similar to an African individual from the 1000 Genomes Project in the range 100,000 to 30,000 yr ago, consistent with Sub-Saharan African ancestry that is relatively recent (Omberg et al. 2012).

### Admixture analysis

The signal of an ancient bottleneck in the Q1 (Bedouin) is not unexpected given previous analyses of genomic admixture that found <1% African ancestry in this subpopulation (Omberg et al. 2012) and studies of worldwide population structure, which have inferred that the Q1 (Bedouin) genomes have the greatest proportion of Arab genetic ancestry, even when compared to Bedouins from outside Qatar and to Arabs in surrounding countries, including Yemen and Saudi Arabia (Hodgson et al. 2014; Shriner et al. 2014). To confirm a similarly minute amount of African admixture for the Q1 (Bedouin) in our sample, we applied three methodologies: (1) an ADMIXTURE (Alexander et al. 2009) analysis of the genome-wide ancestry proportions in the 104 Qataris, the 1000 Genomes Project (The 1000 Genomes Project Consortium 2012), and Human Origins samples (Lazaridis et al. 2014); (2) an ALDER (Loh et al. 2013) analysis of the proportion and timing of African ancestry in these same populations; and (3) a SupportMix (Omberg et al. 2012) analysis of the population assignments of local genomic segments of the 96 Q1 (Bedouin), Q2 (Persian-South Asian), or Q3 (African) Qatari genomes.

The ADMIXTURE analysis identified *K* = 12 ancestral populations as having the lowest cross-validation error (Supplemental Fig. 7A). At this level of resolution, the Q1 (Bedouin) had a high average (84%) proportion of ancestry that was also present in the Human Origins Bedouin B population at a high average proportion (93%) (Supplemental Fig. 7B,C), in which this same ancestry was also shared with Saudis, and at lower levels among other Middle Eastern populations. This ancestry therefore appears to be the signal of an indigenous Arab ancestral population. The Bedouin A population also shared this ancestry but at a lower average proportion (45%) and appeared to be more admixed overall. The Q2 (Persian-South Asian) shared a large proportion (45% on average) of ancestry that dominates in Iranians (46% on average), consistent with a Persian ancestral population (Omberg et al. 2012). The Q3 (African) shared the majority of ancestry with African populations as expected and were considerably admixed overall, again consistent with the known history of this subpopulation (Supplemental Fig. 7A; Omberg et al. 2012).

The ALDER analysis determined the relative percentage of African (Yoruba) ancestry in the Q1 (Bedouin) (2.6% ± 1.37) and Q2 (Persian-South Asian) (5.0% ± 1.41) at levels on par with estimates for other populations sampled in the region (Supplemental Fig. 8; Supplemental Table X), including Human Origins Bedouin and Saudi. This confirmed that recent African admixture is limited to the Q3 (African) subpopulation (37.6% ± 0.9), in which this estimate is on par with African American populations. An estimate of the timing of African admixture placed the number of generations for Q1 (Bedouin) (15.2) and Q2 (Persian-South Asian) (14.0) slightly higher than Q3 (African) (9.3), consistent with the Q1 (Bedouin) and Q2 (Persian-South Asian) reflecting more distant African admixture events and with the Q3 (African) reflecting the historical timing of the African slave trade in the region (Omberg et al. 2012).

The SupportMix analysis used six of the 1000 Genomes populations (two European, two Asian, and two African) (see Supplemental Methods for details) as ancestral proxy reference panels and produced a set of “best guess” admixture assignments based on highest similarity to these genomes. Although these 1000 Genomes populations do not include appropriate local populations most closely related to the Qataris needed for assessment of the true admixture composition of the genomes, the ancestry track length distribution of haplotypes assigned to African populations (Yoruba or Luhuya) provides a qualitative indicator of whether the subpopulations experienced recent admixture with African populations. As expected, the track lengths of the Q1 (Bedouin) and Q2 (Persian-South Asian) assigned to African 1000 Genomes populations were far shorter than those for Q3 (African) (Supplemental Fig. 9), again confirming that recent African admixture is limited to the Q3 (African) subpopulation.

### Neanderthal ancestry

**Figure 4.**
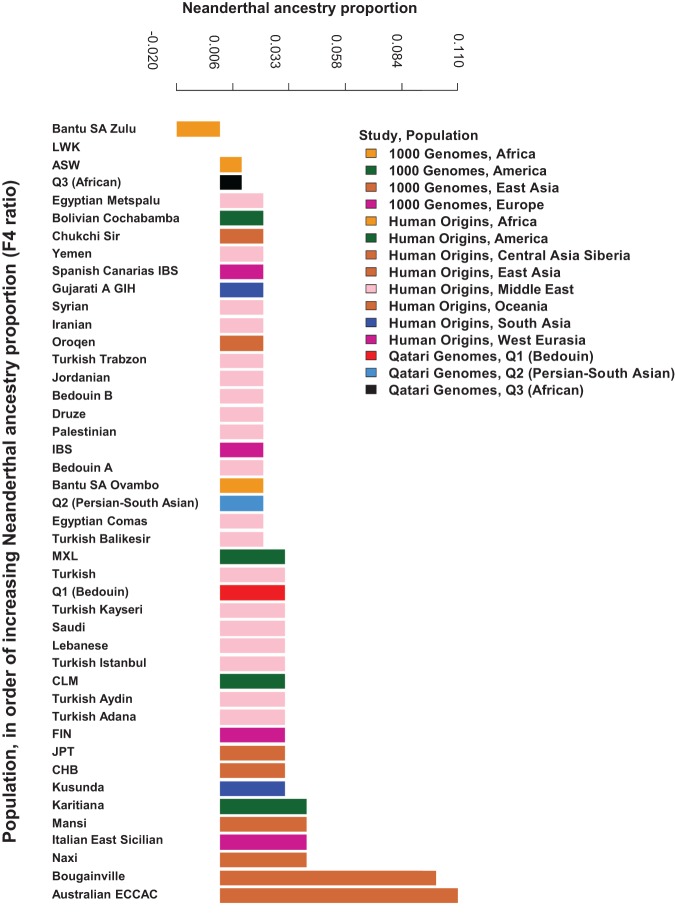
Neanderthal ancestry in world populations. *F*_4_ ratio estimation as implemented in ADMIXTOOLS 3.0 (Patterson et al. 2012) was used to calculate the Neanderthal ancestry proportion for each population in the combined data set of Qatari genomes, the 1000 Genomes Project, and Human Origins. The *F*_4_ ratio estimates α, the proportion of Neanderthal ancestry in a population. Shown are the results for populations of interest, including highest and lowest scoring populations from each region (the 1000 Genomes Project, Africa; the 1000 Genomes Project, America; the 1000 Genomes Project, East Asia, the 1000 Genomes Project, Europe, Human Origins, Africa; Human Origins, America; Human Origins, Central Asia/Siberia; Human Origins, East Asia; Human Origins, Oceania; Human Origins, South Asia; Human Origins, West Eurasia), Middle Eastern populations (Human Origins), Q1 (Bedouin), Q2 (Persian-South Asian) and Q3 (African). Populations are color-coded by region, and a distinct color is used for each Qatari population. A full set of results is presented in Supplemental Figure 10 and Supplemental Table XI. The population codes are as in the 1000 Genomes Project (The 1000 Genomes Project Consortium 2012).

We next analyzed Neanderthal admixture contributions to the ancestry of Q1 (Bedouin) compared to the Q2 (Persian-South Asian) and Q3 (African) Qataris, the 1000 Genomes populations, and the populations of the Human Origins samples using the *F*_4_ ratio and Patterson's *D*-statistic (Fig. 4; Supplemental Fig. 10, Supplemental Table XI; Patterson et al. 2012). The results for both methods were highly correlated (Supplemental Fig. 10A). The Q1 (Bedouin; *F*_4_ ratio = 0.026, *D*-statistic = 0.000) had more Neanderthal admixture than all African populations, including Q3 (African; *F*_4_ ratio range = −0.017 to 0.024, *D*-statistic range = −0.031 to −0.003). The Q1 (Bedouin) also had Neanderthal admixture at levels comparable to Q2 (Persian-South Asian; *F*_4_ ratio = 0.024, *D*-statistic = −0.003) and to other Middle Eastern populations, including other Bedouin populations (Human Origins Bedouin A *F*_4_ ratio = 0.022, *D*-statistic = −0.003 and Bedouin B *F*_4_ ratio = 0.024, *D*-statistic = −0.003) and Saudi (*F*_4_ ratio = 0.026, *D*-statistic = −0.001). Interestingly, the Q1 (Bedouin) did not tend to have higher Neanderthal admixture levels when considering populations outside of the Middle East, where the bulk of European populations had higher Neanderthal admixture (*F*_4_ ratio range = 0.018 to 0.041, *D*-statistic range = 0.003 to 0.010). Yet, the percentage of Neandethal admixture with the Q1 (Bedouin) was higher than expected if it could be entirely explained by later admixture events between the Q1 (Bedouin) and Europeans (observed *F*_4_ ratio = 0.026 versus expected *F*_4_ ratio = 0.00247).

The higher Neanderthal ancestry in the Q1 (Bedouin) Qatari compared to African populations places the divergence of ancestral Arabs after the out-of-Africa bottleneck. Given the current evidence of the geographic range of Neanderthal populations stretching from Europe and the Mediterranean through Northern and Central Asia (Fu et al. 2014; Hershkovitz et al. 2015), the lower Neanderthal Ancestry in the Q1 (Bedouin) Qatari compared to populations within the ancestral Neanderthal range is also consistent with an early divergence of the ancestors of indigenous Arabs from other lineages that populated Asia and Europe. Yet, since the Neanderthal admixture in the Q1 (Bedouin) cannot be entirely explained by admixture with Europeans, this indicates there was some admixture between Neanderthals and ancestors of the Q1 (Bedouin) in the region of the Arabian Peninsula.

### TreeMix analysis

**Figure 5.**
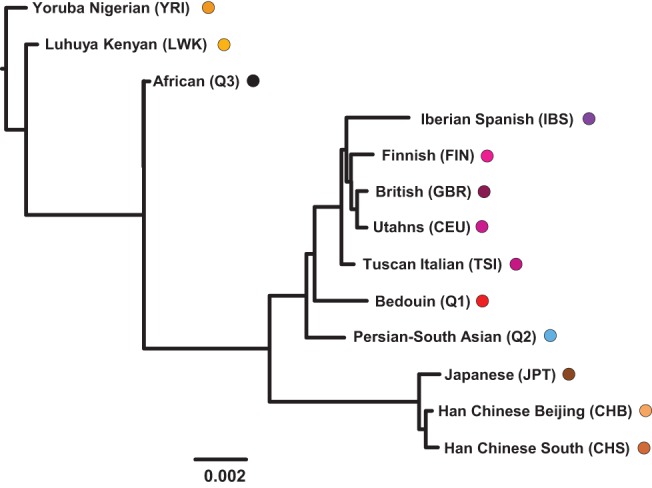
TreeMix (Pickrell and Pritchard 2012) hierarchical clustering analysis of the Q1 (Bedouin), Q2 (Persian-South Asian), and Q3 (African) and the 1000 Genomes Project samples. Shown is a maximum-likelihood tree of population splits inferred without subsequent migration events, in which branch lengths estimate divergence between populations (Europeans in shades of purple: CEU, FIN, GBR, IBS, TSI; East Asians in shades of brown: CHB, CHS, JPT; Africans in shades of orange: LWK, YRI, with the Q1 [Bedouin] in red, Q2 [Persian-South Asian] in azure, and Q3 [African] in black). When allowing from one to five migration events in separate TreeMix analyses, none of the admixture loops connected the Q1 (Bedouin) with any African populations (Supplemental Fig. 10), consistent with the Q1 (Bedouin) having no recent African admixture.

We also analyzed the autosomes of the combined 96 Q1 (Bedouin), Q2 (Perisan-South Asian) or Q3 (African) Qataris, and non-admixed populations of the 1000 Genomes Project using the population split and mixture inference method TreeMix (Pickrell and Pritchard 2012) to assess the relative genetic similarity of populations based on high-density, genome-wide allele frequencies. The analysis returned an overall tree for the 1000 Genomes populations that mirrored those found previously (Shriner et al. 2014) with the addition of the Q1 (Bedouin) and Q2 (Persian-South Asian) clustering on the branch that includes Europeans (Pérez-Miranda et al. 2006) and the Q3 (African) clustering with African populations (Fig. 5). When migrations were allowed in the analysis, no migration events were observed between the Q1 (Bedouin) and African populations, even when allowing as many as five migration events (Supplemental Fig. 11). These results are also consistent with what is known of the migration history of the Arabian Peninsula, including migration both to and from Europe during ancient and more recent eras of civilization, where this resulted in detectable admixture from European populations in both the Q1 (Bedouin) and Q2 (Persian-South Asian) (Omberg et al. 2012).

### Proportion of shared alleles neighbor-joining analysis

As the principal component analysis and the TreeMix population-level clusterings depend on allele frequencies, the clustering of the Q1 (Bedouin) on a common branch with European populations could be driven by the haplotypes introduced by migrants, which would be expected to shift the allele frequencies of these populations toward each other. As such, these clusterings based on allele frequencies do not necessarily argue against significant and deep ancestry of the Q1 (Bedouin) on the Arabian Peninsula, as indicated by the levels of Neanderthal admixture in this subpopulation. Additionally, these population-level clusterings are disproportionately influenced by common segregating alleles (Pickrell and Pritchard 2012), while rare alleles can be more informative about deeper shared ancestry (Mathieson and McVean 2014) as the identity by state of a rare variant can more accurately reflect identity by descent (Hochreiter 2013).

**Figure 6.**
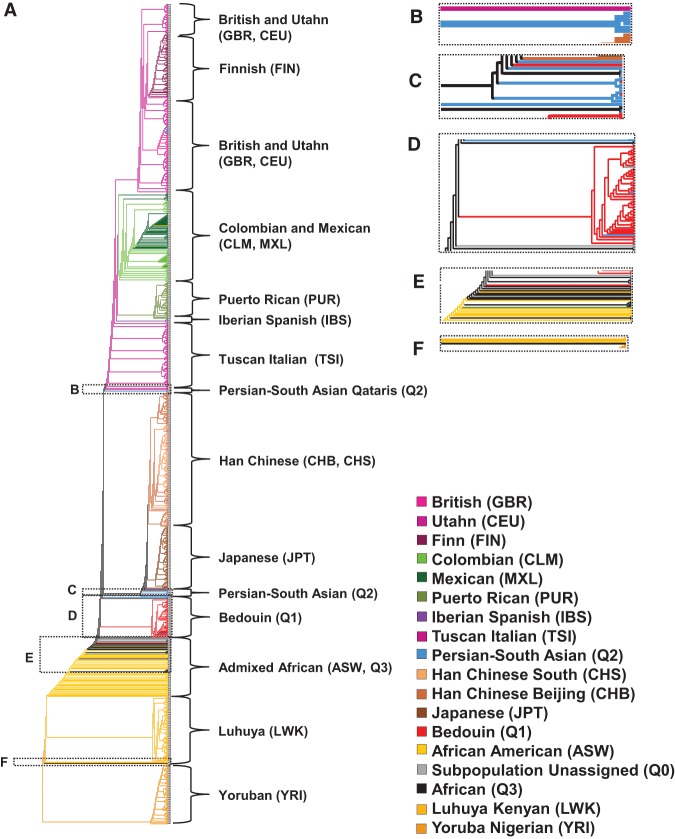
Neighbor-joining tree hierarchical clustering analysis of the combined Qatari genomes and the 1000 Genomes Project Phase 1 samples based on pairwise proportion of shared alleles calculated across the entire autosome. (*A*) The entire neighbor-joining tree with each of the branches leading to individuals in the 1000 Genomes samples color-coded by continent (Europeans in shades of purple: CEU, FIN, GBR, IBS, TSI; Asians in shades of brown: CHB, CHS, JPT; Africans in shades of orange: LWK, YRI, ASW; Americans in shades of green: CLM, MXL, PUR) and with the Q1 (Bedouin) in red, Q2 (Persian-South Asian) in azure, Q3 (African) in black, and Q0 (Subpopulation Unassigned) in gray. (*B*) Detail of the three (15%) Q2 (Persian-South Asian) that cluster with Europeans. (*C*) Detail of the 11 (55%) Q2 (Persian-South Asian) individuals, with three (5%) Q1 (Bedouin), one (5%) Q3 (African), and one (13%) Q0 (Subpopulation Unassigned) that cluster as an outgroup to Asians. (*D*) Detail of the 50 (89%) Q1 individuals, with three (15%) Q2 (Persian-South Asian), one (5%) Q3 (African), and two (25%) Q0 (Subpopulation Unassigned), that cluster outside the Africans and African Ancestry in Southwest US and that also cluster as an outgroup to all other non-African populations, indicating that they are the most distant ancestors of all non-Africans. (*E*) Detail showing the three (15%) Q1 (Bedouin), three (15%) Q2 (Persian-South Asian), 12 (60%) Q3 (African), and four (50%) Q0 (Subpopulation Unassigned) that do not form large clusters but are all located within the admixed cluster. (*F*) Detail of the one (5%) Q3 (African) that clusters between Yoruba (YRI) and Luhya (LWK).

In contrast to population-level clustering, a pairwise clustering of individual genomes based on shared variants provides a relative measure for comparing total shared ancestry between individuals. Also, when applied to a common set of genome-wide, high-density markers that include the low-minor allele frequency alleles of the 1000 Genomes Project, such pairwise clustering also provides an appropriate weight to rare alleles. We therefore performed a proportion of shared alleles (Mountain and Cavalli-Sforza 1997) analysis on the combined samples in the 104 Qatari and the 1000 Genomes samples, in which pairwise proportion of shared alleles was calculated for the 11,711,386 autosomal, biallelic SNPs segregating in both the 104 Qatari and the 1000 Genomes samples. A robust version of the neighbor-joining algorithm was used to perform a pairwise clustering of the samples (Fig. 6A–F; Criscuolo and Gascuel 2008), in which bootstrap support values were calculated for the observed trees using 100 random samplings of the SNPs.

The neighbor-joining analysis revealed that 50 of the 56 Q1 (Bedouin), along with three Q2 (Persian-South Asian), one Q3 (African), and two Q0 (Subpopulation Unassigned) Qataris, clustered outside African lineages and were also the most extreme outgroup that are basal to all non-African populations lacking recent African admixture (Fig. 6D). Strong bootstrap support was observed for this cluster (70 of 100 iterations), and for its presence as an outgroup to the Eurasian cluster (68 of 100 iterations), comparable to the support for the Japanese cluster (60 of 100 iterations) and for the East Asians as an outgroup to Europeans and Americans (81 of 100 iterations). The Q1 (Bedouin) therefore fit the criteria of having ancient migration from Africa and being most distantly related to all other non-Africans in total ancestry.

A total of 11 Q2 (Persian-South Asian), three Q1 (Bedouin), one Q3 (African), and one Q0 (Subpopulation Unassigned) defined an Asian outgroup more closely related to Asians than the main Q1 (Bedouin) outgroup (Fig. 6C), likely driven by the ancestry of the the Q2 (Persian-South Asian) subpopulation traceable to Persia and South Asia (Omberg et al. 2012) and indicating these individuals are most distantly related to other Asians present in this cluster. A total of 12 Q3 (African), three Q1 (Bedouin), three Q2 (Persian-South Asian), and four Q0 (Subpopulation Unassigned) cluster as long individual branches or small clusters between the major Q1 (Bedouin) cluster and the admixed individuals of African ancestry from Southwest US (ASW), potentially representing individuals with a higher proportion of African admixture. As expected from the analyses of population genetic similarity and prior neighbor-joining analysis of admixed populations (Kopelman et al. 2013), the Q3 (African) and African Americans do not form large clusters, but rather appear as multiple individual branches close to the indigenous African populations, most similar to their African admixture source (Fig. 6E,F). A set of three Q2 (Persian-South Asian) clustered as an outgroup to the Tuscan Southern European (TSI) branch (Fig. 6B), which is not unexpected given admixture with European populations (Omberg et al. 2012; Pickrell et al. 2014).

## Discussion

The hypothesis that the first Eurasian populations were established on the Arabian Peninsula and that contemporary indigenous Arabs are direct descendants of this ancient population is supported by two major conclusions derived from the combined evidence of this study. First, the analysis results for X/A diversity, the pairwise sequential Markov coalescent, genome-wide admixture, timing of African admixture, local admixture deconvolution, Neanderthal admixture, and application of TreeMix, support the inference that the Q1 (Bedouin) can trace the bulk of their ancestry back to the out-of-Africa migration events. Second, the combination of lower levels of Neanderthal admixture in the Q1 (Bedouin) than European/Asian populations and the outgroup position of the Q1 (Bedouin) compared to non-Africans in the pairwaise similarity clustering of high-density variants measured genome-wide, place the Q1 (Bedouin) as being the most distant relatives of other contemporary non-Africans. Given that the Q1 (Bedouin) have the greatest proportion of Arab genetic ancestry measured in contemporary populations (Hodgson et al. 2014; Shriner et al. 2014) and are among the best genetic representatives of the autochthonous population on the Arabian Peninsula, these two conclusions therefore point to the Bedouins being direct descendants of the earliest split after the out-of-Africa migration events that established a basal Eurasian population (Lazaridis et al. 2014). This is also consistent with the majority of Q1 (Bedouin) being able to trace a significant portion of their autosomal ancestry through lineages that never left the peninsula after the out-of-Africa migration events since such deep ancestry would not be expected if the entire Arabian Peninsula population had been reestablished from Africa or a non-African population at a later point.

Given the complex history of migration patterns to and from European populations, and the complicated patterns of isolation and intra- and inter-marriage of the indigenous Bedouin populations (Hunter-Zinck et al. 2010; Sandridge et al. 2010), it is not surprising that among the Q1 (Bedouin) are individuals who retain an autosomal signal of being the most distant relatives of non-Africans, while population-level clustering based on migration-shifted allele frequencies places the Q1 (Bedouin) closer to Europeans. The basal position of the Q1 (Bedouin) also has interesting implications for theories about the frequency, timing, and path of major migration waves that established populations in Asia and Europe (Shi et al. 2008; Lazaridis et al. 2014; Shriner et al. 2014). A few isolated Asian populations were previously suspected to be descendants of a separate out-of-Africa migration wave based on Y Chromosome data (Hammer et al. 1998; Shi et al. 2008). Yet, distinct out-of-Africa migration events or separate migration waves emanating from the Arabian Peninsula into Europe and West Asia would be expected to place Bedouins/Europeans and Asians on separate branches of a pairwise clustering tree, distinct from our finding that places the Q1 (Bedouin) as direct descendants of the earliest lineage that split from the ancient non-African population.

A demographic scenario consistent with the evidence presented here is that the population ancestral to the Q1 (Bedouin) migrated out of Africa, and a subset of this population remained in the peninsula until the present day, while a second subset of this population migrated onward and colonized Eurasia. This migration scenario implies the signal of the same bottleneck would be present in all non-African populations, which has been observed thus far in coalescent analysis of contemporary non-African populations (Gronau et al. 2011; Fu et al. 2014; Schiffels and Durbin 2014) and for an anatomically modern human who lived 45,000 yr ago (Fu et al. 2014). This is also consistent with the recent discovery of another anatomically modern human who lived 55,000 yr ago just northeast of the Arabian Peninsula that had morphological features similar to European peoples (Hershkovitz et al. 2015), where this individual could have been a descendant of the basal Eurasian population that remained on the peninsula. Under this migration scenario, although other waves of migration may have occurred, the descendants of these alternative waves either left no descendants or were integrated into the dominant populations.

Beyond the importance for disentangling human migration history, an early split of Eurasian lineages in the Arabian Peninsula has implications for the study of disease genetics for indigenous people in the region. For example, for a disease such as type 2 diabetes that has a prevalence of >18% in the Qatari population, associated genetic variants would not a priori be expected to be the same as those discovered in Europeans, when considering that indigenous Arabs are able to trace a significant portion of their ancestry back to ancient lineages on the Arabian Peninsula. More generally, this suggests that for any genome-wide association study (GWAS) or rare variant association study (RVAS) of diabetes or other complex diseases in Qatar, inference of deep ancestry in the Arabian Peninsula, using rare variation sampled by genome or exome sequencing, is critical for identifying new disease risk genes. Given the dearth of next generation sequencing studies conducted in Middle Eastern and Arab populations, these results indicate that a considerable number of variants that make important contributions to disease risk in these populations are yet to be discovered.

This study is the first analysis of Arabian Peninsula migration making use of deeply sequenced genomes from a sample of unrelated inhabitants of the peninsula. Although there have been many analyses of Chr Y and mtDNA sampled from Arab individuals (Abu-Amero et al. 2007, 2008, 2009; Rowold et al. 2007), and there have been previous surveys of genetic variation of people within the peninsula and immediately surrounding regions conducted with genotyping arrays (Behar et al. 2010; Hunter-Zinck et al. 2010; Alsmadi et al. 2013; Markus et al. 2014; Shriner et al. 2014) and deep exome sequencing (Rodriguez-Flores et al. 2012, 2014; Alsmadi et al. 2014), and by individual high-coverage genomes (Alsmadi et al. 2014; John et al. 2015), the sample of rare and common genetic variation throughout the genome in our sample provides a far more complete picture of how both ancient and recent migration events have contributed to the genetics of the modern peoples of the Arabian Peninsula. For understanding how human migration history has determined the structure of modern genomes, our identification of a cluster of Q1 (Bedouin) as the most distant ancestors of non-Africans is of considerable interest, particularly given the suspected route of migration out of Africa and into the surrounding continents. The possibility that the Q1 (Bedouin) are descendants of the first Eurasians provides an additional piece of the puzzle concerning ancient migration routes and the establishment of ancient non-African populations.

## Methods

### Ethics statement

Human subjects were recruited, and written informed consent was obtained at Hamad Medical Corporation (HMC) and HMC Primary Health Care Centers Doha, Qatar, under protocols approved by the Institutional Review Boards of Hamad Medical Corporation and Weill Cornell Medical College in Qatar.

### Inclusion criteria

Qatar is a peninsula nation on the eastern edge of the Arabian Peninsula (Supplemental Fig. 1). The population of Qatar includes more than 2 million inhabitants, comprised of ∼300,000 nationals with roots in Qatar predating the discovery of oil and gas and establishment of an independent nation in 1970 and the more than 1.7 million immigrants who mostly arrived in the past decade (Qatar Statistics Authority 2013, http://www.qsa.gov.qa/QatarCensus/Pdf/Population above 15 by educational attainment, nationality, age, sex and marital status.pdf). As selection criteria, we required that subjects be third-generation Qataris and all ancestors were Qatari citizens born in Qatar, as assessed by questionnaires. Recent immigrants or residents of Qatar who traced their recent ancestry to other geographic regions were excluded.

Natives of the Arabian Peninsula can be divided into at least three genetic subpopulations that reflect the historical migration patterns in the region: Q1 (Bedouin), Q2 (Persian-South Asian), and Q3 (African) (Hunter-Zinck et al. 2010; Omberg et al. 2012; Rodriguez-Flores et al. 2012). A panel of 48 SNPs was genotyped by TaqMan (Life Technologies) sufficient for classification into one of the three subpopulations based on >70% ancestry in one cluster in a STRUCTURE analysis with *k* = 3 used to identify individuals that could unambiguously be placed in one of these three groups (Supplemental Fig. 2; Pritchard et al. 2000; Rodriguez-Flores et al. 2012). Our primary focus was the Q1 (Bedouin) genetic subpopulation because of its deepest ancestry in Arabia (Ferdinand et al. 1993), so we selected 60 Q1 (Bedouin) individuals to include in the sample. We additionally selected 20 Q2 (Persian-South Asian) and 20 Q3 (African) to use as controls in the analysis, and an additional eight Q0 (Subpopulation Unassigned) individuals that could not be confidently placed in one of these subpopulations, defined as not having >70% ancestry in any of the three groups as determined by STRUCTURE. The total sample therefore included 108 individuals with an even distribution of males and females (see Supplemental Methods; Supplemental Table I).

### Illumina deep sequencing of the genomes

In order to characterize the spectrum of genetic variation, each of the 108 Qatari genomes were sequenced to a median depth of 37× (minimum 30×) through the Illumina Genome Network (see Supplemental Methods for details).

### Relatedness among Qataris

Given the high rate of consanguineous marriage previously reported in the Qatari population (Hunter-Zinck et al. 2010; Mezzavilla et al. 2015), we sought to quantify the relatedness between individuals in our sample and to exclude closely related individuals that could potentially confound population genetics analysis methods that assume the input sample is unrelated. In order to conduct the relatedness analysis, autosomal SNPs in 108 Qatari genomes (described above) were filtered using PLINK 1.9 (Chang et al. 2015), and relatedness between the 108 Qatari genomes was assessed using kinship coefficients estimated by KING-robust (Manichaikul et al. 2010) and PREST-plus (McPeek and Sun 2000) (see Supplemental Methods). Both methods found the same five first-degree and second-degree relationships, in which these relationships were then confirmed by investigative reassessment of medical records. One individual from each of the five pairs of relatives was then excluded from the study. Three of the pairs of relatives formed a trio; hence, two individuals were excluded from the trio, and one individual was excluded from each of the other two pairs, resulting in exclusion of four relatives in total.

### Integration with the 1000 Genomes Project Phase 1

An integrated SNP call set was produced for ancestry analysis for a total of 1200 genomes, combining the 108 Qatari genomes with the 1092 genomes from the 1000 Genomes Project Phase 1 (1000 Genomes) (The 1000 Genomes Project Consortium 2012) (see Supplemental Methods). The integrated call set included 11,711,411 autosomal biallelic SNPs. The transition:transversion ratio of this final set was 2.2, close to values previously observed in the 1000 Genomes Project (The 1000 Genomes Project Consortium 2012). Based on the concordance and quality measures, the calls generated from our pipeline were considered to be high quality, and these were used for all further aspects of this study. After exclusion of four related Qataris (Supplemental Table III), the final integrated call set included 11,711,386 autosomal biallelic SNPs in 1196 genomes.

### Integration with Human Origins data set

The 1000 Genomes Project Phase 1 is an excellent resource for rare variant discovery; however, it is limited in terms of the breadth of global populations sampled. Unfortunately, at the time of writing, no global resource of sequenced genomes existed; hence, the next best alternative for comparison of the Qataris to populations around the world is the “Human Origins Fully Public Dataset” (referred to here as “Human Origins” [HO]), which includes genotype data for 1917 indivduals from Africa, West Eurasia (including Middle East), South Asia, East Asia, Central Asia/Siberia, and America. In particular, the West Eurasian, African, and South Asian data sets include populations sampled in countries close to Qatar, where detection of shared ancestry is of interest in this study. The data set also includes data from archaic genomes, such as Altai Neanderthal, Denisova, and chimpanzee, which are of interest in this study for quantification of Neanderthal ancestry. The Human Origins data set includes a number of samples also present in the 1000 Genomes Project (Supplemental Table IV), and for these samples, the Human Origins overlap data is kept.

In order to conduct population genetic analysis on a combined data set of the 104 Qatari genomes (QG, *n* = 104), the 1000 Genomes Project Phase 1 (1000G-HO, *n* = 1028 after exclusion of duplicates), and Human Origins Fully Public Dataset (HO, *n* = 1862 after exclusion of archaic genomes, ancient genomes, and other genomes not relevant to this study) (Supplemental Table V), a set of sites overlapping between the integrated Qatari genomes plus the 1000 Genomes Project minus Human Origins, and the Human Origins data set were identified. Of 600,841 SNPs in the Human Origins data set and 11,711,386 SNPs in the Qatari genomes plus the 1000 Genomes Project data set, 388,805 SNPs overlapped. Further filtering was conducted on the data set, pruning SNPs based on linkage disequilibrium using PLINK (Purcell et al. 2007), “--indep-pairwise 200 25 0.4,” matching parameters used previously (Lazaridis et al. 2014). After linkage disequilibrium-pruning, the final data set for analysis included 197,714 SNPs segregating in the three data sets (QG, 1000G-HO, and HO).

### Inbreeding coefficient

In order to place the high reported consanguinity in Qatar in a global context, the inbreeding coefficient was calculated using PLINK 1.9 (Chang et al. 2015) for Q1 (Bedouin), Q2 (Persian-South Asian), and Q3 (African) Qataris, the 1000 Genomes Project minus Human Origins overlap, and Human Origins populations (see Supplemental Methods).

### Principal component analysis

A PCA (Price et al. 2006) was carried out for the combined 104 Qatari genomes, the 1000 Genomes Project minus Human Origins overlap, and Human Origins samples using the 197,714 SNPs in the integrated data set (filtering criteria described above). Using the results of this large-scale analysis, visual assessment of clustering and population overlap was used to confirm expected relationships between the analyzed populations. Four distinct plots of a single PCA run were constructed: one comparing the Qatari genomes to the 1000 Genomes populations (Supplemental Fig. 5A), one comparing Qataris to the 1000 Genomes and Human Origins Samples including two visualizations of the full data set (Fig. 1A, color-coded by regional meta-populations; Supplemental Fig. 5B, color-coded by detailed population), and one comparing Qataris to Middle Eastern populations from the Human Origins data set (Fig. 1B). For the latter, in order to compare Qataris to Middle Eastern populations with potential for recent shared Bedouin ancestry with Qataris sampled by the Human Origins data set, populations from the Middle East previously labeled in Lazaridis et al. (2014) as “West Eurasia,” were relabeled as “Middle East,” including Bedouin A, Bedouin B, Druze, Egyptian Comas, Egyptian Metspalu, Iranian, Jordanian, Lebanese, Palestinian, Saudi, Syrian, Turkish, Turkish Adana, Turkish Aydin, Turkish Balikesir, Turkish Istanbul, Turkish Kayseri, Turkish Trabzon, and Yemen.

### Y and mitochondria haplogroup assignment

In order to determine the prevalence of known Chr Y and mtDNA haplogroups in Qatar, SNP genotypes were generated simultaneously for the 108 Qatari genomes using an updated version of GATK (v3.1.1) (DePristo et al. 2011) that supports haploid chromosome calling (*n* = 53 Chr Y, *n* = 108 mtDNA). For one of the genomes, the sample was originally thought to be male but is most likely female due to low call rates on Chr Y. This sample was excluded from Chr Y analysis and X/A diversity analysis, but was included in autosomal and mtDNA analysis. Mean coverage of mapped reads was 11× in Chr Y and 3892× in mtDNA. After exclusion of related and Q0 (Subpopulation Unassigned) (admixed) Qataris, the remaining samples included 47 Chr Y and 96 mtDNA.

Haplogroup assignments for the Chr Y and mtDNA were made using previously characterized variants. For Chr Y, these assignments were made using YFitter (Jostins et al. 2014) by using variants limited to known SNPs cataloged by the International Society of Genetic Genealogy (Jobling and Tyler-Smith 2003) within a 10-Mb interval of the Y Chromosome that is known to be amenable to analysis based on short read sequencing (Skaletsky et al. 2003; Poznik et al. 2013). For mtDNA, these assignments were made using HaploGrep (Kloss-Brandstätter et al. 2011) by using the set of known haplogroup-specific variants in the PhyloTree (van Oven and Kayser 2009) database.

In order to quantify the differences between mtDNA and Chr Y in terms of diversity of the haplogroups identified, the proportion of variance among and within populations was quantified for Chr Y and mtDNA using the AMOVA function in Arlequin (Supplemental Methods; Excoffier et al. 1992; Excoffier and Lischer 2010). The analysis was repeated eight times, including separate analysis of Chr Y and mtDNA, for three-way comparison of the populations, as well as all possible two-way comparisons (Q1/Q2, Q1/Q3, Q2/Q3). The proportion of variance among and within populations was tabulated, as well as the estimated *F*_st_ and *P*-value for both.

### Comparison of X Chromosome to autosomal diversity

The ratio of X-linked to autosomal nucleotide diversity (X/A) for different populations was computed following the approach in Gottipati et al. (2011) and Arbiza et al. (2014) (Supplemental Methods).

### Coalescent analysis

To infer the extent and timing of bottlenecks, the pairwise sequential Markov coalescent (PSMC) (Li and Durbin 2011) was applied to the 96 Q1 (Bedouin), Q2 (Persian-South Asian), or Q3 (African) Qatari genomes. A plot of effective population size versus years in the past was generated for each of the genome using instructions from the PSMC manual (Li and Durbin 2011; see Supplemental Methods). For comparison, the same PSMC pipeline was run on BAM files of Illumina deep sequencing reads mapped to the GRCh37 human reference genome for an individual of European ancestry (NA12878, Utah resident with Northern and Western European ancestry, CEU) and an individual of African ancestry (NA19239, Yoruba in Ibadan, Nigeria, YRI) sequenced as part of the 1000 Genomes Pilot (The 1000 Genomes Project Consortium 2010). The resulting PSMC plots for these two individuals were shifted slightly, such that they align with Qatari PSMC plots at distant (>200,000 yr ago) timescales (Fu et al. 2014).

### Genome-wide admixture analysis

In order to learn more about the ancestry of the sampled Qataris, a genome-wide admixture analysis was conducted on the combined data set of 104 Qatari genomes, the 1000 Genomes Project minus Human Origins overlap, and Human Origins using ADMIXTURE (Supplemental Methods; Alexander et al. 2009). The cross-validation error was calculated for a range of expected number of ancestral populations (*K*), and the *K* with the lowest cross-validation error was used to quantify ancestry, in this case *K* = 12.

### African admixture proportion and timing

In order to estimate the proportion and timing of African admixture in Qatari populations, the genomes of Qataris and world populations were analyzed using ALDER 1.2 (Supplemental Methods; Loh et al. 2013).

### Local admixture analysis

An admixture deconvolution analysis was performed on the 96 Q1 (Bedouin), Q2 (Persian-South Asian), or Q3 (African) Qatari genomes using the 11,711,386 autosomal SNPs segregating in both the 1000 Genomes Project and Qatari genomes using SupportMix (Supplemental Fig. 9; Supplemental Methods; Omberg et al. 2012).

### Neanderthal ancestry

In order to compare the proportion of Neanderthal admixture in Q1 (Bedouin) Qataris with that of other populations in the 1000 Genomes Project (The 1000 Genomes Project Consortium 2012) and Human Origins (Lazaridis et al. 2014), the *F*_4_ ratio (Patterson et al. 2012) and Patterson's *D*-statistic (Patterson et al. 2012) were estimated using the qpF4ratio and qpDstat programs, respectively, from the ADMIXTOOLS 3.0 package (Supplemental Methods; Patterson et al. 2012).

We additionally considered the expected *F*_4_ ratio for the Q1 (Bedouin) under the scenario of no admixture between Neanderthal and direct ancestors of Q1 (Bedouin), such that observed Neanderthal ancestry in Q1 (Bedouin) would be entirely due to European admixture. From the estimated components of the ADMIXTURE analysis with *K* = 12, the Southern European ancestry in the Q1 (Bedouin) is 8.2% on average, and the Northern European ancestry in Q1 (Bedouin) is 1.3% on average, totaling 9.5% of the genome. If the Q1 (Bedouin) had never mixed with Neanderthal prior to introduction of European admixture, assuming no selection against introgressed genomic intervals, we would therefore expect an *F*_4_ ratio in Q1 (Bedouin) to be on the order of 1/10 of those observed in European populations.

### TreeMix analysis

We performed a TreeMix analysis (Pickrell and Pritchard 2012) of the 96 Q1 (Bedouin), Q2 (Persian-South Asian), or Q3 (African) Qatari genomes and the 1000 Genomes Project excluding admixed populations (Puerto Rican, Mexican, Colombian, and African Ancestry in Southwest US) (Supplemental Methods).

### Neighbor-joining tree clustering

In order to determine if any of the Qatari genomes were the most distant ancestors of all non-African populations, neighbor-joining trees were constructed for the 104 Qatari genomes and the 1000 Genomes Project using the 11,711,386 autosomal SNPs segregating in both data sets. For each pair of genomes, the proportion of shared alleles (PSA) (Mountain and Cavalli-Sforza 1997), or 1 minus the proportion of the genome identical by state (IBS), was calculated using the “--distance -square -1-ibs” function in PLINK 1.9 (Purcell et al. 2007; Chang et al. 2015), which outputs a 1196×1196 matrix of distances (1 minus IBS distance or PSA). A neighbor-joining (NJ) tree was constructed using a recently updated version of the original NJ (Saitou and Nei 1987) algorithm called NJS (Criscuolo and Gascuel 2008) that is better at handling missing values, as implemented in the APE package in R (Paradis et al. 2004; R Core Team 2014). Overall, this approach is computationally tractable for millions of markers genotyped in thousands of genomes and produces similar topologies to maximum-likelihood clustering methods but requires only a fraction of the compute time, where the trade-off is a sacrifice in the accuracy of branch lengths (Tateno et al. 1994). The algorithm takes the distance matrix as input and outputs a tree. In order to confirm the robustness to sample ordering, the order of samples in the matrix was shuffled and reclustered 100 times, in which all reclusterings recovered the same tree. In order to produce bootstrap support values for the tree, 100 reclusterings of the tree were generated based on random sampling of SNPs. For each bootstrap iteration, 11,711,386 random (with replacement) SNPs were selected using a Python script (www.python.org), and then the PSA distance matrix and NJ tree were recalculated using these SNPs. Bootstrap support was calculated using the Python package SumTrees (Sukumaran and Holder 2010).

For visualization, the tree was rooted at the most recent common ancestor (MRCA) node of the largest cluster of the 1000 Genomes Yoruba (YRI) genomes in the tree. A color version of the tree was produced using TreeGraph 2 (Stöver and Müller 2010) by manually coloring the branches leading to each node. A single color is assigned to each population, with populations from the same continent having similar colors: Europeans in shades of purple, Asians in shades of brown, Americans in shades of green, Africans in shades of orange, Q1 (Bedouin) in red, Q2 (Persian-South Asian) in blue, Q3 (Sub-Saharan African) in black, and Q0 (Subpopulation Unassigned) in gray. When a cluster of nodes includes different populations, the terminal branches were given population-specific colors, whereas the shared higher-order branches for the cluster were given the color of the population in majority. For example, if 10 Q1 (Bedouin) and 1 Q0 (Subpopulation Unassigned) were in a cluster, the branches above where the nodes come together were colored red.

## Data access

The sequence data generated for this study in BAM format, as well as SNP genotypes in VCF format, have been submitted to the NCBI Sequence Read Archive (SRA; http://www.ncbi.nlm.nih.gov/sra/) under accession number SRP060765. Allele frequencies for known and novel genomic SNPs have been submitted to NCBI dbSNP (http://www.ncbi.nlm.nih.gov/SNP/) under submitter batch ID QG108_GENOMIC_SNPS_20151008 (http://www.ncbi.nlm.nih.gov/SNP/snp_viewBatch.cgi?sbid=1062298) and submitter handle WEILL_CORNELL_DGM. PLINK and VCF files of genotypes for variants analyzed in this study, both before and after integration with 1000 Genomes and Human Origins, are available on our website http://geneticmedicine.weill.cornell.edu/genome.html.

## Supplementary Material

Supplemental Material
