# Supplementary material for: Indigenous Arabs are descendants of the earliest split from ancient Eurasian populations
Source: Genome Res. 2016 Feb;26(2):151–62. doi: 10.1101/gr.191478.115 (PMC4728368; doi:10.1101/gr.191478.115)
Supplement: Supplemental Material [file supp_26_2_151__index.html]

Indigenous Arabs are descendants of the earliest split from ancient Eurasian populations — Indigenous Arabs are descendants of the earliest split from ancient Eurasian populations — Supplemental Material 

# Indigenous Arabs are descendants of the earliest split from ancient Eurasian populations

## Supplemental Material

**Files in this Data Supplement:**

- Supplemental Data.pdf
