# Supplementary material for: Indigenous Arabs are descendants of the earliest split from ancient Eurasian populations
Source: Genome Res. 2016 Feb;26(2):151–62. doi: 10.1101/gr.191478.115 (PMC4728368; doi:10.1101/gr.191478.115)

Supplemental Methods

Inclusion Criteria

In the total sample of 108 individuals, given the large percentage of adult Qataris with diabetes (18%) (International Diabetes Federation 2014), we attempted to include a representative percentage of diabetics in the sample. Our sample includes 15 male Q1(Bedouin), 15 female Q1 (Bedouin), 5 male Q2 (Persian-South Asian), 5 female Q2 (Persian-South Asian), 5 male Q3 (African), 5 female Q3 (African), 4 male Q0 (Subpopulation Unassigned) males, and 4 female Q0 (Subpopulation Unassigned). Beyond diabetes and conditions closely associated with diabetes (e.g., obesity), none of the diabetic or healthy individuals presented any other health issues or diseases, genetic or otherwise, as determined by inspection of their electronic medical record. Selection of the study sample was also designed to produce an even distribution of males and females (Supplemental Table I).

Illumina Deep Sequencing of the Genomes

Next-generation sequencing was conducted using a sequencing library preparation method that eliminates the need for size selection after shearing and PCR amplification before ligation of sequencing adapters. Sequencing was conducted at the Illumina Genome Services sequencing facility using the HiSeq 2500. Sufficient paired-end 100 bp reads were generated in order to produce a median of 112 GB of sequence data passing filters and aligned to the hg19/GRCh37 human reference genome with a median insert size of 301 bp, where at least 85% of bases with quality score ≥30 (Q30) passed filtering steps and were aligned. Among non-N bases in the reference genome, at least 98% were covered by at least one base in all 108 genomes.

The Illumina Genome Network generated variant calls for the autosomal chromosomes for each of the 108 Qatari genomes using the ELAND/CASAVA v1.9 pipeline (Bentley 2008).

To provide a variant call set when simultaneously considering the entire Qatari sample to compare to the 1092 genomes from 1000 Genomes Project Phase 1 (The 1000 Genomes Project Consortium 2012) (1000 Genomes), the Qatari genotypes were re-mapped and re-called using an in-house population genotyping pipeline (Rodriguez-Flores 2014). Reads were realigned to the 1000 Genomes Project version of the hg19/GRCh37 human reference genome using BWA 0.5.9 (Li and Durbin 2009) (maximum insert size 3 kb), and mapped reads were prepared for variant calling using GATK "Best practices" (DePristo 2011), including PCR duplicate removal using SAMtools (Li 2009), producing an average of 37* depth in autosomal chromosomes, with a mean of 98% of mappable sites covered per genome (Supplemental Table II).

In order to maximize computational efficiency and integrated call set quality, calling for the autosomes was restricted to the biallelic SNPs in the combined set of 22.9 million segregating autosomal SNPs observed at least once in the Qatari genomes by the CASAVA pipeline (Bentley 2008) and the 39.7 million autosomal SNPs in 1000 Genomes Phase 1 SNP sites (Phase 2 calls are not available genome-wide and Phase 3 calls are under embargo as of manuscript submission), including an overlap of 15.5 million SNPs observed in both call sets. SNP genotypes for Qatari genomes were simultaneously called using GATK as described in the GATK "best practices" recommended workflow (DePristo 2011). This produced an average of 4,242,255 SNPs per genome or 23,784,210 SNPs in total. The quality of our re-generated call set was evaluated by assessing concordance with the Illumina Genome Network calls as well as independent exome sequencing calls for 30 of the genomes (Rodriguez-Flores 2014). Concordance between the Qatari genomes for the CASAVA and GATK variant calls was >99.6% at depths ≥4* and concordance was >99.2% for exome calls (Bentley 2008; DePristo 2011).

Relatedness among Qataris.

In order to conduct the relatedness analysis, autosomal SNPs in 108 Qatari genomes were

filtered using PLINK 1.9 run with flags '--geno 0.05', '--hwe 0.05', and '--indep-pairwise 1000 25 0.25'. After filtering, 1,407,483 SNPs remained for relatedness analysis. The relatedness between the 108 Qatari genomes was assessed using kinship coefficients estimated by KING-robust (Manichaikul 2010) using the flag '--kinship' and PREST-plus (McPeek and Sun 2000) using the flag '--aped'. No known pedigree information was included in either programs' analysis. Thresholds to determine degree of relatedness for the KING-robust analysis (first-, second-, or third-degree, or unrelated) were: 0.354 > first-degree ≥ 0.177 > second-degree ≥ 0.1101 > third-degree ≥ 0.0442. These values were those recommended (Manichaikul 2010), except for our second-degree threshold value which was found via detection of known relatives in HapMap and 1000 Genomes analysis (The 1000 Genomes Project Consortium 2012).

Integration with 1000 Genomes Project Phase 1

In order to integrate the call sets, the autosomal VCF files were converted to PLINK (Purcell 2007) files containing all biallelic autosomal SNPs (22,958,844 in the Qatari genomes and 39,706,744 in 1000 Genomes) using PLINK 1.9 (Chang 2015). The missing genotype rate was calculated in each call set, sites with a missing genotype rate over 10% in either call set were excluded. Next, the minor allele frequency was calculated for the remaining SNPs, and a list of sites where the major and minor alleles match in both call sets was generated. The two call sets were then merged using PLINK, limited to the sites with matching major/minor allele. Finally, sites with Hardy-Weinberg equilibrium probability $<10^{-6}$ were excluded, as were SNPs within 10 bp of each other (potential indels), and the quality of the integrated call set was assessed by inspection of the log10 site frequency spectrum (not shown).

Inbreeding Coefficient

The reported rate of consanguineous marriage in the Qatari population is high, and in previous studies (Hunter-Zinck 2010; Mezzavilla 2015) a higher proportion of homozygosity is

observed in Q1 (Bedouin) compared to 1000 Genomes. For global context, the inbreeding coefficient was calculated using PLINK 1.9 (Chang 2015) for Q1 (Bedouin), Q2 (Persian-South Asian), and Q3 (African) Qataris, 1000 Genomes minus Human Origins overlap, and Human Origins populations. The inbreeding coefficient for each individual was calculated using the linkage disequilibrium-pruned set of 197,714 SNPs, and the mean and standard deviation for each population was calculated in R (R Core Team 2015). Populations were sorted in order of decreasing mean inbreeding coefficient, and plotted for comparison.

Y Chromosome and Mitochondria Haplogroup Assignment

In order to quantify the differences between Mitochondrial DNA (MtDNA) and Y Chromosome (ChrY) in terms of diversity of haplogroups identified, the proportion of variance among and within populations was quantified for ChrY and MtDNA using Arlequin (Excoffier 1992; Excoffier and Lischer 2010). Genotypes for all segregating sites in ChrY and MtDNA of unrelated non-admixed Qataris, including 56 Q1 (Bedouin) MtDNA, 20 Q2 (Persian-South Asian) MtDNA, 20 Q3 (African) MtDNA, 27 Q1 (Bedouin) ChrY, 10 Q2 (Persian-South Asian) ChrY, and 10 Q3 (African) ChrY, were converted to Arlequin input format using a Python script (Python Software Foundation 2015), and the proportion of variance was estimated using the AMOVA function in Arlequin, which reports the proportion of variance among and within Q1 (Bedouin), Q2 (Persian-South Asian), and Q3 populations (African), as well as F_{st} (Weir and Cockerham 1984). The analysis was repeated 8 times, including separate analysis of ChrY and MtDNA, for three-way comparison of the populations, as well as all possible 2-way comparisons (Q1/Q2, Q1/Q3, Q2/Q3).

Comparison of X Chromosome to Autosomal Diversity

The ratio of X-linked to autosomal nucleotide diversity (X/A) was computed following the approach in Gottipati et al (Gottipati 2011) and Arbiza et al (Arbiza 2014), where estimates

normalized by divergence to macaque for the X Chromosome (ChrX) and the autosomes, were used to obtain the ratio of X/A diversity using whole genome variant calls in 52 unrelated females in this study and from 567 females in the latest release of the 1000 Genomes Phase I over the exact same set of regions. Genotypes were generated for female ChrX using GATK 3.1, and the initial set of 814,568 ChrX SNPs and 22,958,844 autosomal SNPs were subsequently filtered down to genomic regions of interest for quantification of diversity in females. Regions were obtained by filtering to exclude genomic segments likely to be under the influence of selective constraints and several quality filters for inter-species or intra-population estimates of genetic variation. To build the final set of filters we compiled several tracks from UCSC Genome Browser (hg19) (Kent 2002). These consisted of the union of repetitive regions according to the Tandem Repeats Finder35 (simple-repeats track), regions with high CpG content (CpG-island track), centromeres and telomeres along with 2 Mb flanking regions, gaps in the human assembly, conserved noncoding elements (PhastConsElements44WayPlacental and PhastConsElements44WayPrimate tracks), pseudoautosomal regions on ChrX, segmental duplications, regions corresponding to various gene transcripts (union of UCSC, RefSeq, and GENCODE gene tracks), regions of poor human-macaque synteny (hg19 *vs* rheMac2 syntenic-net track) or gaps in their alignment (Mutliz44way alignment), and genomic regions prone to poor sequencing results as denoted by the strict call-ability masks released by the 1000 Genomes project. After applying filters, a total of 43,999,783 bp of X-linked and 695,776,796 bp of autosomal sequence were used to estimate X/A for in each population. Within these genomic intervals, a total of 133,713 ChrX SNPs and 3,284,862 autosomal SNPs segregating in both Qatari and 1000 Genomes females were kept for analysis.

Estimates of nucleotide diversity (the average number of pairwise differences per base between all haploid samples in a population) normalized by human-macaque divergence (the

proportion of differences between the human and macaque reference after Jukes-Cantor correction) were estimated over 100 kb loci obtained by grouping bases along chromosomes that remained after filtering. We refer to these normalized diversity estimates for individual populations in ChrX, the autosomes, or the ratio of X/A as absolute diversity estimates (Gottipati 2011; Arbiza 2014). Absolute X/A diversity in individual populations is influenced by several biases affecting ChrX and the autosomes differently, including the effect of selection on genic sites in linkage disequilibrium with intergenic regions (Gottipati 2011; Arbiza 2014), we also used genetic distance to the nearest gene (HapMap recombination rates, scaled by two-thirds for ChrX to yield sex-averaged rates, and RefSeq genes) to obtain a collection of regions that are far from genes and the least influenced by any such effect. To do this we partitioned all bases passing filters to each of seven bins spanning distances between 0.0 and 0.4 cM in such a manner that an equal fraction of bases in ChrX fit each bin and estimated absolute X/A diversity in all bases falling in the 7th and last bin (0.18 – 0.40cM) using the same procedures described above.

We also consider relative X/A diversity estimates, defined as the ratio of diversity estimates between a given pair of populations (e.g., relative X/A diversity). Standard errors of the mean for absolute and relative diversity estimates in ChrX, the autosomes, and X/A were obtained by a moving block bootstrap procedure (Liu and Singh 1992; Lahiri 2003; Keinan 2007), where 10,000 random data sets were produced by resampling with replacement from the full set of 100 kb loci, independently for ChrX and the autosomes, and used to compute standard errors for all estimates.

Coalescent Analysis

The pairwise sequential Markov coalescent (PSMC) (Li and Durbin 2011) was applied to the 96 Q1 (Bedouin), Q2 (Persian-South Asian), or Q3 (African) Qatari genomes. A plot of effective population size *vs* years in the past was generated for each of the genome using

instructions from the PSMC manual (Li and Durbin 2011), first generating genome-wide calls using SAMtools mpileup command (Li 2009) then applying the PSMC algorithm to infer demographic history for each sample. The parameters in the PSMC model include the mutation rate (μ) and generation time (g) of $\mu = 1.2 \times 10^{-8}$ and g=25 years (Roach 2010). Since PSMC outputs different x-axis coordinates for each individual genome, in order to calculate the average effective population size across multiple time points for each population, a spline was fitted to each PSMC output. The spline fitting function, as implemented in the R lattice package (lattice: Trellis Graphics for R 2015), outputs effective population size in 1000 year intervals from 1000 years ago to 1 million years ago. The median was calculated for Q1 (Bedouin), Q2 (Persian-South Asian), and Q3 (African) and plotted (Figure 3).

Genome-Wide Admixture Analysis

A genome-wide admixture analysis was conducted on the combined dataset of 104 Qatari genomes, 1000 Genomes minus Human Origins overlap, and Human Origins (see Supplemental Methods). Using the integrated and linkage disequilibrium-pruned dataset of 197,714 SNPs, the genome-wide ancestry proportions were calculated using ADMIXTURE (Alexander 2009). The ADMIXTURE algorithm takes as input a dataset and a K value which indicates the expected number of ancestral populations for the dataset, and outputs the proportion of ancestry in each ancestry population for each individual. The result is visualized in a plot generated using R, which color-codes each ancestral group. In order to determine the optimal K, the analysis was run for K=3 to K=18, and the cross validation error was calculated for each iteration.

Three visualizations of the data were produced. The first includes all genomes in the combined dataset, sorted by dataset (96 Q1, Q2 or Q3 Qatari genomes, 1000 Genomes minus Human Origins, and Human Origins) and then by region (Africa, America, Central Asia / Siberia, East Asia, South Asia, and West Eurasia) as defined in the supplement of Lazardis et al

2014 (Lazaridis 2014). In this analysis, the lowest cross validation error was observed for K=12, and at this level of resolution, an ancestry component that dominates in Q1 (Bedouin) Qataris, Saudi, and Bedouin B populations was observed. A second visualization includes populations defined here as Middle Eastern, as well as populations in the K=12 analysis with >10% admixture in the Bedouin B component that also dominates in Q1 (Bedouin) Qataris and Saudi. A third visualization looks at the K=12 results for both the full dataset and the Middle Eastern dataset (Supplemental Figure 7).

African Admixture Proportion and Timing

ALDER 1.2 (Loh 2013) was used to analyze the proportion and timing of African admixture in Qatari populations, the genomes of Qataris and world populations. For each Q1 (Bedouin), Q2 (Persian-South Asian), or Q3 (African) Qatari and Human Origins population, Yoruba was used as a reference panel, and the proportion and timing of African admixture was estimated with confidence intervals established using a block jackknife (Reich 2009). A result was obtained for a subset of the populations; the remainder resulted in ALDER runtime errors due to small sample size or high error estimates.

Local Admixture Analysis

We performed an admixture deconvolution analysis on the 96 Q1 (Bedouin), Q2 (Persian-South Asian), or Q3 (African) Qatari genomes using the 11,711,386 autosomal SNPs segregating in both 1000 Genomes and Qatari genomes. The Qatari genomes and 1000 Genomes autosomal haplotypes were phased using SHAPEIT2 using default parameter settings (Delaneau 2013) and SupportMix (Omberg 2012) was used to assign 2000 SNP autosomal segments of each Qatari using populations of the 1000 Genomes as “ancestral proxy reference panels”. Six of the 14 populations were used, including two European, two Asian, and two African. The European populations were CEU (Utah residents with Central and Western European ancestry) and TSI

(Tuscan Italian), the Asian populations were CHB (Han Chinese Beijing) and JPT (Japanese in Tokyo), and the African populations were YRI (Yoruba in Nigeria) and LWK (Luhuya in Kenya) (see Supplemental Table IV for population details). The input parameters of SupportMix included the phased reference populations (1000 Genomes), the generations to admixture (2,400, assuming generation time of 25 years and 60,000 years to admixture), and the ancestry inference window size (2,000 SNPs, approximately 0.5 cM). SupportMix divides each chromosome into intervals and infers the most similar population for each haplotype. Based on the results, a tract length distribution was constructed using a Python script that scans each haplotype, counting the length of each interval where the haplotype is assigned to the same continental population (African, European, or Asian).

The distribution of African ancestry tracts was tabulated for each Qatari population in R (R Core Team 2015) and plotted for visualization (Supplemental Figure 9). While in theory, identity by descent for an ancient African ancestry tract 2,400 generations ago would be <1 cM, with the poor sampling of populations closely related to Qatari in the 1000 Genomes "proxy reference panel," we expect more and larger tract assignments to African populations, such that the analysis provided a relative metric for assessing ancient *vs* recent African ancestry in the Qatari genomes.

Neanderthal Ancestry

In order to compare the proportion of Neanderthal admixture in Q1 (Bedouin) Qataris with that of other populations in 1000 Genomes (The 1000 Genomes Project Consortium 2012) and Human Origins (Lazaridis 2014), the qpF4ratio program in the AdmixTools 3.0 package (Patterson 2012) was used to calculate the F_4 ratio and the qpDstat program in the AdmixTools 3.0 package (Patterson 2012) was used to calculate the *D-statistic*.

The F_4 ratio estimates α, the proportion of Neanderthal ancestry in the focal population by detecting introgression based on incomplete lineage sorting as reflected in the allele frequencies (Reich 2009). The F_4 ratio is calculated for 5 populations, including one outgroup (O = chimpanzee), two archaic (A = Denisova, B = Neanderthal) and two contemporary (C = Yoruba, X = tested population). The model is that population B contributed α percent ancestry to population X (via a subset of B called B'), and population C contributed 1 – α (via a subset of C called C'). The F_4 ratio is obtained by dividing two f_4 statistics (f_4 (A,O;X,C) / f_4(A,O;B,C)). The f_4 statistic detects introgression based on incomplete lineage sorting, which is reflected in the allele frequencies (Reich 2009).

The *D-statistic* assesses counts of derived Neanderthal alleles *vs* ancestral (Chimpanzee) alleles in Q1 (Bedouin) and a compared population. The *D-statistic* was calculated for 4 populations (W = tested population, X = Q1, Y = Neanderthal, Z = Chimp), inspecting sites where Y and Z differ. The counts are the number of sites where the Y (derived) allele is observed in W and the Z (ancestral) allele is observed in X, and vice versa. An excess of population Y (Neanderthal) alleles in population W compared to Q1 (Bedouin) results in a higher *D-statistic* score, which translates to higher Neanderthal ancestry in population W compared to Q1 (Bedouin).

The F_4 ratio and the *D-statistic* relative to Q1 (Bedouin) was calculated for each population in the combined Qatari genome (QG), 1000 Genomes minus Human Origins overlap (1000G-HO), and Human Origins (HO) dataset for populations from Africa, West Eurasia, Central Asia and Siberia, East Asia, South Asia, Oceania, Middle East, and America. Neanderthal and Chimpanzee genotypes for this analysis were obtained from the Human Origins dataset. For populations that overlap between 1000 Genomes and Human Origins, the analysis was conducted twice, once for the 1000 Genomes (excluding duplicates in Human Origins) and a

second time for the Human Origins sample, where the 1000 Genomes populations are labeled using 3-letter codes (e.g. "YRI"), while Human Origins populations are labeled using full labels from the Lazardis et al. 2014 supplement (e.g. "Yoruba").

TreeMix Analysis

We performed a TreeMix analysis (Pickrell and Pritchard 2012) of the 96 Q1 (Bedouin), Q2 (Persian-South Asian), or Q3 (African) Qatari genomes and 1000 Genomes excluding admixed populations (PUR, MXL, CLM, and ASW; abbreviations defined in Supplemental Table IV). After exclusion of admixed populations, the allele count for 11,701,491 biallelic SNPs was calculated using PLINK (Chang 2015), and Python (Python Software Foundation 2015) was used to convert this file to a TreeMix input file with reference and alternate allele counts for each SNP in each population. The maximum likelihood trees reflecting population splits among the potentially mixing populations were produced using default settings, allowing from 0-5 migrations (mixtures), each in a separate analysis (Supplemental Figure 11). Trees in the supplement were plotted using FigTree 1.4 (Rambaut 2015), and residuals were plotted using the TreeMix R package R functions (Pickrell and Pritchard 2012). For each migration event observed, both the direction and percentage of admixture was recorded. In each successive run of TreeMix, the original tree was included as a reference, and the migration edges were inferred *de novo*.

Supplemental Table I. Summary of 3-generation Qataris Selected for Deep Genome Sequencing[1]

		Males		Female	
**Population**	**Total**	**Non-diabetic**	**Type 2 diabetic**	**Non-diabetic**	**Type 2 diabetic**
3-generation Qataris	108	27	27	27	27
Q1 (Bedouin)	60	15	15	15	15
Q2 (Persian-South Asian)	20	5	5	5	5
Q3 (African)	20	5	5	5	5
Q0 (Subpopulation Unassigned)	8	2	2	2	2

[1] Deep genome sequencing was conducted for 108 Qataris with over 3 generations of ancestry in Qatar, including equal numbers of males/females and diabetics/nondiabetics from the three major ancestry groups in Qatar. Shown are the numbers of genomes sequenced and subpopulation assignment based on 48 SNPs genotyped by TaqMan, where individuals were assigned to a subpopulation (Q1, Q2, or Q3) based on >70% ancestry in one group as determined by a STRUCTURE analysis (Pritchard 2000; Rodriguez-Flores 2012), and individuals having <70% ancestry in all groups placed in the Subpopulation Unassigned group (Q0).

Supplemental Table II. Variants Identified in the 108 Qatari Genomes[1]

Statistic	Chromosomes				
	Total (%)	Autosomal	Female ChrX[3]	Male ChrY	MtDNA
**Per individual[2]**					
Samples	108	108	54	53	108
Alleles	216	216	108	53	108
Mapped sequence (Gb)	112	111.8	5.6	0.11	0.065
Surveyed genome (Mb) (%)	2,754 (98)	2,593 (98)	151.1 (99)	10.4 (45)	0.018 (100)
Mean mapped depth	37	37	37	11	3,892
Mean biallelic SNPs per individual (% novel)	4,242,225 (5.7)	4,127,441 (5.7)	113,756 (28)	1,015 (22)	32 (32)
Mean Ti:Tv per individual	2.1	2.1	1.8	1.5	27[5]
**Population[4]**					
Number of SNPs (% novel)	23,784,210 (30)	22,958,844 (30)	814,568 (39)	10,037 (63)	761 (64)
Ti:Tv SNPs	2.2	2.1	1.8	1.6	18

1 108 Qatari genomes were sequenced (see Supplemental Table I) using paired-end 100 bp Illumina reads on a HiSeq 2500. Reads were mapped to hg19/GRCh37 using two separate pipelines. First, the CASAVA 1.9 pipeline (Bentley 2008) was used to discover potential variant sites in each individual genome. In the second phase, the GATK best practices workflow (DePristo 2011) was used to simultaneously call genotypes in all 108 Qatari genomes at all potentially variant sites identified by the CASAVA pipeline. Reads were remapped using BWA 0.5.9 (Li and Durbin 2009), PCR duplicate reads were removed using SAMtools (Li 2009), and variants were called from reads of mapping quality >10 and bases with quality >17 using GATK (DePristo 2011). GATK 0.2.6 was used for autosomes, while GATK 3.1 was used for ChrX, ChrY, and MtDNA in order to take advantage of the haploid chromosome calling algorithm implemented in this version. Autosomal genotyping was limited to potential variant sites identified by the CASAVA pipeline (Bentley 2008) and 1000 Genomes Project Phase 1 sites (The 1000 Genomes Project Consortium 2012), while ChrY genotyping was limited to 10 Mb of ChrY amenable to next-generation sequencing analysis, MtDNA genotyping included the complete Cambridge reference sequence (Anderson 1981), and ChrX genotyping included all non-N bases on the chromosome. A summary of mapping and genotyping results is presented, including per-genome means and population (n=108) totals.

2 Shown is the mean of each statistic per genome for the 108 Qatari genomes (with percentages in parentheses). Each result is shown for the complete genome: autosomal sites (Chr1 to Chr22), ChrX in females, the 10 Mb of ChrY amenable to next-generation sequencing analysis, and MtDNA in all individuals. One of the individuals originally considered male has no calls on ChrY, hence genotypes are included for only autosomes and MtDNA in this individual. From top-to-bottom is shown the number of individuals (n); number of alleles (2n for autosomes, n for ChrY and MtDNA); total gigabases mapped to the 1000 Genomes Project version of the hg19/GRCh37 reference genome by BWA 0.5.9; surveyed genome mean number of genome sites mapped with ≥1 read and % of mappable genome; mean depth of mapped reads among sites with at least one read mapped; mean number and % novel (not in DbSNP 135 nor 1000 Genomes Phase 1) of biallelic SNPs per genome; and transition-to-transversion (Ti:TV) ratio for biallelic SNPs. * Due to divide-by-zero errors in samples with no transversions, Ti:Tv ratios were calculated on n=74 samples with at least one transversion.

3 Statistics are shown for female ChrX only, as these were used for calculation of ChrX to autosome diversity (Gottipati 2011; Arbiza 2014).

4 Shown are the population totals for the 108 Qatari genomes (with percentage error in parentheses). From top to bottom are shown the total number of biallelic SNPs and percent novel; the Ti:Tv ratio of biallelic SNPs. Each result is shown for the total genome: autosomal chromosomes, ChrX in females, ChrY in males, and MtDNA.

Supplemental Table III. Identification of First-degree and Second-degree Relatives in 108 Qatari Genomes[1]

Relatives						Relationship degree			Inference							
1st Relative (excluded)			2nd Relative				Confirmed		Observed IBS counts			Kinship Coefficient		Estimated IBD (PREST)		
ID	Pop	Gender	ID	Pop	Gender	Inferred	Degree	Relationship	IBS=0	IBS=1	IBS=2	KING	PREST	IBD=0	IBD=1	IBD=2
DGMQ-31105	Q1	M	DGMQ-31177	Q1	F	1	1	Siblings	0.0025	0.0824	0.9153	0.2348	0.2375	0.2734	0.5032	0.2234
DGMQ-31416	Q1	M	DGMQ-32061	Q1	F	1	1	Siblings	0.0020	0.0812	0.9170	0.2629	0.2527	0.253	0.4831	0.2639
DGMQ-31105	Q1	M	DGMQ-31123	Q1	M	2	2	Uncle	0.0060	0.1085	0.8862	0.1237	0.0931	0.8138	0	0.1862
DGMQ-31177	Q1	F	DGMQ-31123	Q1	M	2	2	Aunt	0.0056	0.1105	0.8846	0.1328	0.0905	0.819	0	0.181
DGMQ-31513	Q1	F	DGMQ-31717	Q1	F	2	1	Sisters	0.0054	0.1123	0.8829	0.1435	0.1014	0.7973	0	0.2027

[1] Although the 108 genomes selected for sequencing were sampled at random from the Qatari population, a high rate of consanguineous marriage is documented in the population (Hunter-Zinck 2010; Sandridge 2010; Mezzavilla 2015), and hence there is a high risk of obtaining related Qataris in a random sample, in particular with the Q1 (Bedouin) population. The relatedness in the 108 Qataris was assessed using the kinship coefficients estimated by KING-robust (Manichaikul 2010) using the flag '--kinship' and by PREST-plus (McPeek and Sun 2000) using the flag '--aped'. The genome data was first filtered via PLINK 1.9's (Chang 2015) '--geno 0.05', '--hwe 0.05', and '--indep-pairwise 1000 25 0.25' flags, leaving 1,407,483 SNPs from the initial 22.9 million autosomal SNPs for analysis. No known pedigree information was included in either programs' analysis. Both methods found the same five first- and second-degree relationships using Manichaikul et al.'s recommended cutoff values of 0.177 for first-degree. For second-degree relationships, a value of 0.1101 was used as the threshold [selected based on parallel analysis of known relationships in HapMap and 1000 Genomes data (The 1000 Genomes Project Consortium 2012; Stevens 2012)]. One of the second-degree relationships was reported subsequent to sample selection to be a full-siblingship, but according to KING's and PREST-plus's estimated kinship coefficients (0.1435 and 0.1014, respectively) is most likely a second-degree relationship, likely a half-siblingship. Shown are the ID, population, and gender of the 1st and 2nd relative in the pair, the inferred and confirmed relationship degree, the confirmed actual relationship, observed IBS proportions, inferred kinship coefficients using KING-robust and PREST-plus, and the estimated IBD proportions inferred by PREST-plus.

Supplemental Table IV. 1000 Genomes Project Phase 1 Samples Included in Analyses[1]

Group	Code	Description	Total	Male	Female
1000 Genomes Phase 1	ALL		1092	525	567
African	AFR		246	115	131
	LWK	Luhya in Webuye, Kenya	97	48	49
	YRI	Yoruba in Ibadan, Nigeria	88	43	45
	ASW	African Ancestry in Southwest US	61	24	37
American	AMR		181	88	93
	CLM	Colombian in Medellin, Colombia	60	29	31
	MXL	Mexican Ancestry in Los Angeles, California	66	31	35
	PUR	Puerto Rican in Puerto Rico	55	28	27
Asian	ASN		286	144	142
	CHB	Han Chinese in Beijing, China	97	44	53
	CHS	Southern Han Chinese, China	100	50	50
	JPT	Japanese in Tokyo, Japan	89	50	39
European	EUR		379	178	201
	CEU	Utah residents with Northern and Western European ancestry	85	45	40
	FIN	Finnish in Finland	93	35	58
	GBR	British in England and Scotland	89	41	48
	IBS	Iberian populations in Spain	14	7	7
	TSI	Toscani in Italy	98	50	48

[1] The 1000 Genomes Project Phase 1(The 1000 Genomes Project Consortium 2012) samples analyzed in combination with the Qatari samples, prior to integration with Human Origins data. Analyses included principal component analysis (Supplemental Figure 5B), X-linked to autosomal diversity (Supplemental Figure 6), coalescent analysis (Figure 3), local admixture analysis (Supplemental Figure 9), TreeMix (Pickrell and Pritchard 2012) (Figure 5, Supplemental Figure 11) and pairwise neighbor-joining cluster analysis (Figure 6). Shown are the group, population code, description of populations sampled, and the total number of males and females in each.

Supplemental Table V. Populations from Human Origins, 1000 Genomes Phase 1, and Qatar included in Analyses[1]

Source[2]	Region[3]	Population[3]	Sample size[4] (N)
		Total	2994
1000G-HO		Total 1000 Genomes Phase 1 not in Human Origins (Lazaridis 2014)	1028
HO		Total Human Origins samples (Lazaridis 2014)	1862
QG		Total Qatari genomes	104
1000G-HO	Africa	ASW	61
1000G-HO	Africa	LWK	95
1000G-HO	Africa	YRI	57
1000G-HO	America	CLM	60
1000G-HO	America	MXL	66
1000G-HO	America	PUR	55
1000G-HO	East Asia	CHB	97
1000G-HO	East Asia	CHS	100
1000G-HO	East Asia	JPT	89
1000G-HO	Europe	CEU	85
1000G-HO	Europe	FIN	86
1000G-HO	Europe	GBR	76
1000G-HO	Europe	IBS	3
1000G-HO	Europe	TSI	98
HO	Africa	AA Denver	12
HO	Africa	Algerian	7
HO	Africa	Bantu SA Herero	2
HO	Africa	Bantu SA Ovambo	1
HO	Africa	Bantu SA Pedi	1
HO	Africa	Bantu SA S Sotho	1
HO	Africa	Bantu SA Tswana	2
HO	Africa	Bantu SA Zulu	1
HO	Africa	BantuKenya	6
HO	Africa	BiakaPygmy	20
HO	Africa	Datog	3
HO	Africa	Esan Nigeria ESN	8
HO	Africa	Gambian GWD	6
HO	Africa	Hadza Henn	5
HO	Africa	Ju hoan North	5
HO	Africa	Khomani	11
HO	Africa	Kikuyu	4
HO	Africa	Luhya Kenya LWK	8
HO	Africa	Luo	8
HO	Africa	Mandenka	17
HO	Africa	Masai Ayodo	2
	Africa	Masai Kinyawa MKK	10

Supplemental Table V. Populations from Human Origins, 1000 Genomes Phase 1, and Qatar included in Analyses[1] (continued, page 2)

Source[2]	Region[3]	Population[3]	Sample size[4] (N)
HO	Africa	MbutiPygmy	10
HO	Africa	Mende Sierra Leone MSL	8
HO	Africa	Mozabite	21
HO	Africa	Saharawi	6
HO	Africa	Somali	13
HO	Africa	Tunisian	8
HO	Africa	Yoruba	70
HO	America	Bolivian Cochabamba	1
HO	America	Bolivian LaPaz	3
HO	America	Bolivian Pando	3
HO	America	Karitiana	12
HO	America	Mayan	18
HO	America	Mixe	10
HO	America	Mixtec	10
HO	America	Piapoco	4
HO	America	Pima	14
HO	America	Quechua Coriell	5
HO	America	Surui	8
HO	America	Zapotec	10
HO	Central Asia Siberia	Aleut	7
HO	Central Asia Siberia	Altaian	7
HO	Central Asia Siberia	Chukchi	20
HO	Central Asia Siberia	Chukchi Reindeer	1
HO	Central Asia Siberia	Chukchi Sir	2
HO	Central Asia Siberia	Dolgan	3
HO	Central Asia Siberia	Eskimo Chaplin	4
HO	Central Asia Siberia	Eskimo Naukan	13
HO	Central Asia Siberia	Eskimo Sireniki	5
HO	Central Asia Siberia	Even	10
HO	Central Asia Siberia	Itelmen	6
HO	Central Asia Siberia	Kalmyk	10
HO	Central Asia Siberia	Koryak	9
HO	Central Asia Siberia	Kyrgyz	9
HO	Central Asia Siberia	Mansi	8
HO	Central Asia Siberia	Mongola	6
HO	Central Asia Siberia	Nganasan	11
HO	Central Asia Siberia	Selkup	10
HO	Central Asia Siberia	Tajik Pomiri	8
HO	Central Asia Siberia	Tlingit	4
HO	Central Asia Siberia	Tubalar	22
HO	Central Asia Siberia	Turkmen	7
HO	Central Asia Siberia	Tuvinian	10
HO	Central Asia Siberia	Ulchi	25
HO	Central Asia Siberia	Uzbek	10
HO	Central Asia Siberia	Yakut	20
HO	Central Asia Siberia	Yukagir Forest	5
HO	Central Asia Siberia	Yukagir Tundra	14
HO	East Asia	Ami Coriell	10
HO	East Asia	Atayal Coriell	9
HO	East Asia	Cambodian	8
HO	East Asia	Dai	10
HO	East Asia	Daur	9
HO	East Asia	Han	33
HO	East Asia	Han NChina	10

Supplemental Table V. Populations from Human Origins, 1000 Genomes Phase 1, and Qatar included in Analyses[1] (continued, page 3)

Source[2]	Region[3]	Population[3]	Sample size[4] (N)
HO	East Asia	Hezhen	8
HO	East Asia	Japanese	29
HO	East Asia	Kinh Vietnam KHV	8
HO	East Asia	Korean	6
HO	East Asia	Lahu	8
HO	East Asia	Miao	10
HO	East Asia	Naxi	9
HO	East Asia	Oroqen	9
HO	East Asia	She	10
HO	East Asia	Thai	10
HO	East Asia	Tu	10
HO	East Asia	Tujia	10
HO	East Asia	Uygur	10
HO	East Asia	Xibo	7
HO	East Asia	Yi	10
HO	Middle East	Bedouin A	25
HO	Middle East	Bedouin B	19
HO	Middle East	Druze	39
HO	Middle East	Egyptian Comas	11
HO	Middle East	Egyptian Metspalu	7
HO	Middle East	Iranian	8
HO	Middle East	Jordanian	9
HO	Middle East	Lebanese	8
HO	Middle East	Palestinian	38
HO	Middle East	Saudi	8
HO	Middle East	Syrian	8
HO	Middle East	Turkish	4
HO	Middle East	Turkish Adana	10
HO	Middle East	Turkish Aydin	7
HO	Middle East	Turkish Balikesir	6
HO	Middle East	Turkish Istanbul	10
HO	Middle East	Turkish Kayseri	10
HO	Middle East	Turkish Trabzon	9
HO	Middle East	Yemen	6
HO	Oceania	Australian ECCAC	3
HO	Oceania	Bougainville	10
HO	Oceania	Papuan	14
HO	South Asia	Balochi	20
HO	South Asia	Bengali Bangladesh BEB	7
HO	South Asia	Brahui	21
HO	South Asia	Burusho	23
HO	South Asia	Gujarati A GIH	5
HO	South Asia	Gujarati B GIH	5
HO	South Asia	Gujarati C GIH	5
HO	South Asia	Gujarati D GIH	5
HO	South Asia	Hazara	14
HO	South Asia	Kalash	18
HO	South Asia	Kusunda	10
HO	South Asia	Makrani	20
HO	South Asia	Pathan	19
HO	South Asia	Punjabi Lahore PJL	8
HO	South Asia	Sindhi	18
HO	West Eurasia	Abkhasian	9
HO	West Eurasia	Adygei	17

Supplemental Table V. Populations from Human Origins, 1000 Genomes Phase 1, and Qatar included in Analyses[1] (continued, page 4)

Source[2]	Region[3]	Population[3]	Sample size[4] (N)
HO	West Eurasia	Albanian	6
HO	West Eurasia	Armenian	10
HO	West Eurasia	Balkar	10
HO	West Eurasia	Basque French	20
HO	West Eurasia	Basque Spanish	9
HO	West Eurasia	Belarusian	10
HO	West Eurasia	Bulgarian	10
HO	West Eurasia	Chechen	9
HO	West Eurasia	Chuvash	10
HO	West Eurasia	Croatian	10
HO	West Eurasia	Cypriot	8
HO	West Eurasia	Czech	10
HO	West Eurasia	English Cornwall GBR	5
HO	West Eurasia	English Kent GBR	5
HO	West Eurasia	Estonian	10
HO	West Eurasia	Finnish FIN	7
HO	West Eurasia	French	25
HO	West Eurasia	French South	7
HO	West Eurasia	Georgian Megrels	10
HO	West Eurasia	Greek Comas	14
HO	West Eurasia	Greek Coriell	6
HO	West Eurasia	Hungarian Coriell	10
HO	West Eurasia	Hungarian Metspalu	10
HO	West Eurasia	Icelandic	12
HO	West Eurasia	Italian Bergamo	12
HO	West Eurasia	Italian East Sicilian	5
HO	West Eurasia	Italian South	1
HO	West Eurasia	Italian Tuscan	8
HO	West Eurasia	Italian West Sicilian	6
HO	West Eurasia	Kumyk	8
HO	West Eurasia	Lezgin	9
HO	West Eurasia	Lithuanian	10
HO	West Eurasia	Maltese	8
HO	West Eurasia	Mordovian	10
HO	West Eurasia	Nogai	9
HO	West Eurasia	North Ossetian	10
HO	West Eurasia	Norwegian	11
HO	West Eurasia	Orcadian	13
HO	West Eurasia	Russian	22
HO	West Eurasia	Saami WGA	1
HO	West Eurasia	Sardinian	27
HO	West Eurasia	Scottish Argyll Bute GBR	4
HO	West Eurasia	Spanish Andalucia IBS	4
HO	West Eurasia	Spanish Aragon IBS	6
HO	West Eurasia	Spanish Baleares IBS	4
HO	West Eurasia	Spanish Canarias IBS	2
HO	West Eurasia	Spanish Cantabria IBS	5
HO	West Eurasia	Spanish Castilla la Mancha IBS	5
HO	West Eurasia	Spanish Castilla y Leon IBS	5
HO	West Eurasia	Spanish Cataluna IBS	5
HO	West Eurasia	Spanish Extremadura IBS	5
HO	West Eurasia	Spanish Galicia IBS	5
HO	West Eurasia	Spanish Murcia IBS	4
HO	West Eurasia	Spanish Pais Vasco IBS	5

Supplemental Table V. Populations from Human Origins, 1000 Genomes Phase 1, and Qatar included in Analyses[1] (continued, page 5)

Source[2]	Region[3]	Population[3]	Sample size[4] (N)
HO	West Eurasia	Spanish Valencia IBS	5
HO	West Eurasia	Ukrainian East	6
HO	West Eurasia	Ukrainian West	3
QG	Middle East	Q0 (Subpopulation Unassigned)	8
QG	Middle East	Q1 (Bedouin)	54
QG	Middle East	Q2 (Persian-South Asian)	20
QG	Middle East	Q3 (African)	20

[1] Due to the limited geographic diversity of the 1000 Genomes Project Phase 1 sample (The 1000 Genomes Project Consortium 2012), populations from the Human Origins sample (Lazaridis 2014) were analyzed in cases where diversity of geographic sampling is more important than deep sampling of rare alleles by genome sequencing: principal components analysis (Figure 1 and Supplemental Figure 5A), inbreeding coefficient (Supplemental Figure 4), genome-wide admixture analysis (Supplemental Figure 7), proportion of African admixture (Supplemental Figure 8), and Neanderthal ancestry analysis (Figure 4 and Supplemental Figure 10).

[2] 1000 Genomes Project Phase 1 data was downloaded from the 1000 Genomes Project website (1000G-HO) (The 1000 Genomes Project Consortium 2012). The Human Origins dataset was downloaded from the David E. Reich laboratory website (HO) (Lazaridis 2014). In cases where samples overlapped, the Human Origins data was kept and the 1000 Genomes duplicate was removed, hence “1000G-HO”. Genotype data for the two studies (1000G-HO, HO) were integrated with genotypes for Qatari genomes sequenced in this study (QG), limited to sites segregating in all three datasets [197,714 SNPs after linkage disequilibrium pruning using PLINK 1.9 (Chang 2015)].

[3] For Human Origins samples, the region and population ID of most samples are as in the Lazardis et al. 2014 supplemental materials. Human Origins populations from the Middle East of interest for comparison to Qatari genomes in this study were given a separate regional label, originally they were labeled as part of the broader “West Eurasian” group. The Middle Eastern populations include Druze, Palestinian, Bedouin A, Bedouin B, Egyptian Comas, Turkish Adana, Turkish Istanbul, Turkish Kayseri, Jordanian, Turkish Trabzon, Iranian, Lebanese, Saudi, Syrian, Egyptian Metspalu, Turkish Aydin, Turkish Balikesir, Yemen, Turkish, and Qataris (Q1, Q2, Q3). For 1000 Genomes individuals already present in the Human Origins dataset, the region was assigned as in Lazardis 2014. For the remaining 1000 Genomes individuals, the population label is a 3-letter code as used in the 1000 Genomes Phase 1 paper (The 1000 Genomes Project Consortium 2012). For example, the “Yoruba” population in this study represents the Human Origins data, while the “YRI” population represents the 1000 Genomes sample not duplicated in Human Origins.

[4] For Human Origins samples, the sample size is as in the Lazardis 2014 supplemental materials. In cases where Human Origins and 1000 Genomes samples overlapped, the Human Origins data was kept and the 1000 Genomes duplicate was removed, hence 1000 Genomes population sample sizes are reduced compared to Supplemental Table IV. The Qataris include 104 total genomes, where n=4 Q1 (Bedouin) of the total 108 sequenced were excluded based on a first-degree or second-degree relationship to another individual in the sample (Supplemental Table III).

Supplemental Table VI. Inbreeding Coefficients of Populations[1]

Population	Study	Region	Inbreeding coefficient	
			Mean	St Dev
Surui	HO	America	0.5300	0.0194
Karitiana	HO	America	0.5019	0.0218
Piapoco	HO	America	0.4655	0.0176
Pima	HO	America	0.4599	0.0247
Mixe	HO	America	0.4523	0.0136
Mixtec	HO	America	0.4127	0.0161
Zapotec	HO	America	0.4097	0.0189
Eskimo Naukan	HO	Central Asia Siberia	0.4087	0.0096
Bolivian LaPaz	HO	America	0.4060	0.0790
Itelmen	HO	Central Asia Siberia	0.4047	0.0065
Koryak	HO	Central Asia Siberia	0.4013	0.0232
Eskimo Chaplin	HO	Central Asia Siberia	0.4001	0.0134
Quechua Coriell	HO	America	0.3938	0.0255
Nganasan	HO	Central Asia Siberia	0.3919	0.0126
Chukchi	HO	Central Asia Siberia	0.3911	0.0121
Bolivian Pando	HO	America	0.3865	0.0315
Chukchi Sir	HO	Central Asia Siberia	0.3856	0.0079
Eskimo Sireniki	HO	Central Asia Siberia	0.3845	0.0261
Mayan	HO	America	0.3844	0.0400
Atayal Coriell	HO	East Asia	0.3834	0.0145
Ami Coriell	HO	East Asia	0.3653	0.0095
Yukagir Tundra	HO	Central Asia Siberia	0.3603	0.0211
Ulchi	HO	Central Asia Siberia	0.3500	0.0208
She	HO	East Asia	0.3402	0.0081
Yakut	HO	Central Asia Siberia	0.3393	0.0083
Tujia	HO	East Asia	0.3381	0.0191
Lahu	HO	East Asia	0.3381	0.0136
Oroqen	HO	East Asia	0.3377	0.0162
Hezhen	HO	East Asia	0.3375	0.0090
Miao	HO	East Asia	0.3369	0.0048
JPT	1000G-HO	EAS	0.3346	0.0096
Japanese	HO	East Asia	0.3344	0.0102
Korean	HO	East Asia	0.3316	0.0044
CHB	1000G-HO	EAS	0.3314	0.0069

Supplemental Table VI. Inbreeding Coefficients of Populations[1] (continued, page 2)

Population	Study	Region	Inbreeding coefficient	
			Mean	St Dev
Han	HO	East Asia	0.3313	0.0044
CHS	1000G-HO	EAS	0.3312	0.0056
Daur	HO	East Asia	0.3307	0.0067
Dai	HO	East Asia	0.3300	0.0068
Xibo	HO	East Asia	0.3281	0.0049
Han N China	HO	East Asia	0.3264	0.0045
Naxi	HO	East Asia	0.3253	0.0047
Yi	HO	East Asia	0.3239	0.0168
Mongola	HO	Central Asia Siberia	0.3227	0.0078
Tu	HO	East Asia	0.3158	0.0065
Selkup	HO	Central Asia Siberia	0.3134	0.0284
Kinh Vietnam KHV	HO	East Asia	0.3096	0.0216
Tuvinian	HO	Central Asia Siberia	0.3061	0.0194
Mansi	HO	Central Asia Siberia	0.3037	0.0079
Papuan	HO	Oceania	0.3020	0.0108
Tubalar	HO	Central Asia Siberia	0.3001	0.0098
Kalmyk	HO	Central Asia Siberia	0.2989	0.0095
Tlingit	HO	Central Asia Siberia	0.2963	0.0281
Dolgan	HO	Central Asia Siberia	0.2896	0.0625
Altaian	HO	Central Asia Siberia	0.2870	0.0138
Thai	HO	East Asia	0.2869	0.0119
Even	HO	Central Asia Siberia	0.2864	0.0532
Aleut	HO	Central Asia Siberia	0.2852	0.0324
Kyrgyz	HO	Central Asia Siberia	0.2850	0.0133
Cambodian	HO	East Asia	0.2805	0.0157
Kusunda	HO	South Asia	0.2740	0.0630
Bougainville	HO	Oceania	0.2732	0.0051
Australian ECCAC	HO	Oceania	0.2649	0.0327
Kalash	HO	South Asia	0.2638	0.0205
Turkish Balikesir	HO	Middle East	0.2625	0.0208
FIN	1000G-HO	EUR	0.2615	0.0072
Hazara	HO	South Asia	0.2598	0.0143
Basque Spanish	HO	West Eurasia	0.2596	0.0111
Finnish FIN	HO	West Eurasia	0.2586	0.0086
Estonian	HO	West Eurasia	0.2585	0.0062

Supplemental Table VI. Inbreeding Coefficients of Populations[1] (continued, page 3)

Population	Study	Region	Inbreeding coefficient	
			Mean	St Dev
Lithuanian	HO	West Eurasia	0.2568	0.0162
Chuvash	HO	West Eurasia	0.2566	0.0069
Russian	HO	West Eurasia	0.2563	0.0041
Icelandic	HO	West Eurasia	0.2550	0.0061
Turkish Trabzon	HO	Middle East	0.2549	0.0296
IBS	1000G-HO	EUR	0.2543	0.0060
Uygur	HO	East Asia	0.2542	0.0127
Balochi	HO	South Asia	0.2529	0.0410
Mordovian	HO	West Eurasia	0.2512	0.0082
Basque French	HO	West Eurasia	0.2511	0.0124
Spanish Pais Vasco IBS	HO	West Eurasia	0.2508	0.0069
Turkmen	HO	Central Asia Siberia	0.2502	0.0233
Orcadian	HO	West Eurasia	0.2500	0.0110
Belarusian	HO	West Eurasia	0.2497	0.0049
French South	HO	West Eurasia	0.2487	0.0090
GBR	1000G-HO	EUR	0.2482	0.0076
English Cornwall GBR	HO	West Eurasia	0.2470	0.0051
Scottish Argyll Bute GBR	HO	West Eurasia	0.2468	0.0096
Sardinian	HO	West Eurasia	0.2459	0.0104
Norwegian	HO	West Eurasia	0.2454	0.0088
Turkish Kayseri	HO	Middle East	0.2452	0.0168
CEU	1000G-HO	EUR	0.2447	0.0076
Ukrainian East	HO	West Eurasia	0.2437	0.0045
Czech	HO	West Eurasia	0.2436	0.0059
Tajik Pomiri	HO	Central Asia Siberia	0.2430	0.0211
Hungarian Coriell	HO	West Eurasia	0.2430	0.0063
English Kent GBR	HO	West Eurasia	0.2428	0.0049
North Ossetian	HO	West Eurasia	0.2423	0.0044
Hungarian Metspalu	HO	West Eurasia	0.2414	0.0045
Adygei	HO	West Eurasia	0.2411	0.0082
Croatian	HO	West Eurasia	0.2402	0.0089
Balkar	HO	West Eurasia	0.2392	0.0071
Turkish Istanbul	HO	Middle East	0.2389	0.0193
Italian Bergamo	HO	West Eurasia	0.2373	0.0121
Pathan	HO	South Asia	0.2369	0.0238
Albanian	HO	West Eurasia	0.2365	0.0144
Greek Comas	HO	West Eurasia	0.2355	0.0095
French	HO	West Eurasia	0.2350	0.0103
Spanish Aragon IBS	HO	West Eurasia	0.2349	0.0026
Sindhi	HO	South Asia	0.2347	0.0322
Georgian Megrels	HO	West Eurasia	0.2346	0.0093

Supplemental Table VI. Inbreeding Coefficients of Populations[1] (continued, page 4)

			Inbreeding coefficient	
Population	Study	Region	Mean	St Dev
Italian Tuscan	HO	West Eurasia	0.2339	0.0087
Greek Coriell	HO	West Eurasia	0.2331	0.0186
Spanish Cantabria IBS	HO	West Eurasia	0.2329	0.0117
Spanish Cataluna IBS	HO	West Eurasia	0.2328	0.0094
Chechen	HO	West Eurasia	0.2326	0.0197
Kumyk	HO	West Eurasia	0.2326	0.0129
TSI	1000G-HO	EUR	0.2323	0.0074
Uzbek	HO	Central Asia Siberia	0.2323	0.0381
Nogai	HO	West Eurasia	0.2310	0.0193
Abkhasian	HO	West Eurasia	0.2304	0.0233
Burusho	HO	South Asia	0.2294	0.0138
MXL	1000G-HO	AMR	0.2291	0.0632
Yukagir Forest	HO	Central Asia Siberia	0.2273	0.0458
Spanish Castilla la Mancha IBS	HO	West Eurasia	0.2272	0.0108
Spanish Valencia IBS	HO	West Eurasia	0.2260	0.0054
Turkish	HO	Middle East	0.2259	0.0035
Lezgin	HO	West Eurasia	0.2250	0.0346
Brahui	HO	South Asia	0.2249	0.0346
Bulgarian	HO	West Eurasia	0.2245	0.0195
Turkish Aydin	HO	Middle East	0.2243	0.0084
Turkish Adana	HO	Middle East	0.2234	0.0220
Armenian	HO	West Eurasia	0.2230	0.0159
Spanish Baleares IBS	HO	West Eurasia	0.2229	0.0104
Gujarati D GIH	HO	South Asia	0.2226	0.0097
Gujarati B GIH	HO	South Asia	0.2223	0.0153
Ukrainian West	HO	West Eurasia	0.2217	0.0489
Gujarati C GIH	HO	South Asia	0.2215	0.0061
Cypriot	HO	West Eurasia	0.2212	0.0115
Gujarati A GIH	HO	South Asia	0.2211	0.0050
Punjabi Lahore PJL	HO	South Asia	0.2192	0.0043
Iranian	HO	Middle East	0.2153	0.0219
Spanish Castilla y Leon IBS	HO	West Eurasia	0.2150	0.0092
Druze	HO	Middle East	0.2149	0.0224
Spanish Murcia IBS	HO	West Eurasia	0.2128	0.0077
Bengali Bangladesh BEB	HO	South Asia	0.2122	0.0108
Makrani	HO	South Asia	0.2102	0.0487
Spanish Galicia IBS	HO	West Eurasia	0.2081	0.0101
Italian WestSicilian	HO	West Eurasia	0.2075	0.0159
Spanish Andalucia IBS	HO	West Eurasia	0.2060	0.0047
Italian EastSicilian	HO	West Eurasia	0.2057	0.0086
Spanish Extremadura IBS	HO	West Eurasia	0.2036	0.0146

Supplemental Table VI. Inbreeding Coefficients of Populations[1] (continued, page 5)

Population	Study	Region	Inbreeding coefficient	
			Mean	St Dev
Lebanese	HO	Middle East	0.1936	0.0742
Bedouin B	HO	Middle East	0.1917	0.0325
Saudi	HO	Middle East	0.1911	0.0408
Maltese	HO	West Eurasia	0.1870	0.0098
Spanish Canarias IBS	HO	West Eurasia	0.1862	0.0197
Syrian	HO	Middle East	0.1792	0.0268
Jordanian	HO	Middle East	0.1569	0.0533
Q1 (Bedouin)	QG	Middle East	0.1548	0.1068
CLM	1000G-HO	AMR	0.1487	0.1189
Palestinian	HO	Middle East	0.1425	0.0305
Bedouin A	HO	Middle East	0.1208	0.0625
Q2 (Persian-South Asian)	QG	Middle East	0.1084	0.1465
PUR	1000G-HO	AMR	0.0731	0.1169
Egyptian Comas	HO	Middle East	0.0327	0.0234
Egyptian Metspalu	HO	Middle East	0.0146	0.0307
Tunisian	HO	Africa	-0.0013	0.0417
Saharawi	HO	Africa	-0.0037	0.0402
Yemen	HO	Middle East	-0.0100	0.1623
Mozabite	HO	Africa	-0.0200	0.0321
Algerian	HO	Africa	-0.0305	0.0256
Somali	HO	Africa	-0.4190	0.0466
Datog	HO	Africa	-0.6450	0.0426
Masai Kinyawa MKK	HO	Africa	-0.6526	0.0293
Masai Ayodo	HO	Africa	-0.7084	0.0015
ASW	1000G-HO	AFR	-0.7117	0.1876
Q3 (African)	QG	Middle East	-0.7323	0.3754
AA Denver	HO	Africa	-0.7672	0.1144
Kikuyu	HO	Africa	-0.7976	0.0215
Hadza Henn	HO	Africa	-0.8014	0.1104
Mandenka	HO	Africa	-0.8519	0.0195
Gambian GWD	HO	Africa	-0.8656	0.0246
Luhya Kenya LWK	HO	Africa	-0.9001	0.0218
BantuKenya	HO	Africa	-0.9037	0.0130
Luo	HO	Africa	-0.9138	0.0193
Esan Nigeria ESN	HO	Africa	-0.9158	0.0253
Yoruba	HO	Africa	-0.9285	0.0241
LWK	1000G-HO	AFR	-0.9286	0.0246
YRI	1000G-HO	AFR	-0.9373	0.0201
Mende Sierra Leone MSL	HO	Africa	-0.9459	0.0239
Bantu SA Herero	HO	Africa	-1.0107	0.0773
Biaka Pygmy	HO	Africa	-1.1687	0.0325
Mbuti Pygmy	HO	Africa	-1.2050	0.0289

Supplemental Table VI. Inbreeding Coefficients of Populations[1] (continued, page 6)

Population	Study	Region	Inbreeding coefficient	
			Mean	St Dev
Bantu SA Tswana	HO	Africa	-1.2774	0.1270
Khomani	HO	Africa	-1.5816	0.1529
Ju hoan North	HO	Africa	-1.9050	0.0863

[1] In order to compare inbreeding in Qatar to other world populations, the inbreeding coefficient (fhat) was calculated using PLINK 1.9 (Chang 2015) for each individual in the combined 96 Q1 (Bedouin), Q2 (Persian-South Asian), or Q3 (African) Qatari, populations from the Human Origins (Lazaridis 2014) datasets, and populations from the 1000 Genomes Project Phase 1 (The 1000 Genomes Project Consortium 2012) excluding individuals in the Human Origins dataset. The population mean and standard deviation (St Dev) for each population was calculated using R (lattice: Trellis Graphics for R 2015). Plink outputs 3 estimates of inbreeding, named "fhat1", "fhat2", and "fhat3", in this analysis the "fhat2" estimate was used. Shown are the region, population, inbreeding coefficient mean, and standard deviation for each Qatari (QG), 1000 Genomes minus Human Origins samples (1000G-HO), and Human Origins (HO) populations with sufficient sample size.

Supplemental Table VII. AMOVA Results[1]

Comparison	Statistic	MtDNA	ChrY
Q1,Q2,Q3	Variance Among	4.6	11.74
Q1,Q2,Q3	Variance Within	95.4	88.26
Q1,Q2,Q3	F_{st}	0.04605	0.11736
Q1,Q2,Q3	P-value	<1e-5	<1e-5
Q1,Q2	Variance Among	2.11	6.25
Q1,Q2	Variance Within	97.89	93.75
Q1,Q2	F_{st}	0.02106	0.06252
Q1,Q2	P-value	0.03421	0.0176
Q1,Q3	Variance Among	7.41	18.57
Q1,Q3	Variance Within	92.59	81.43
Q1,Q3	F_{st}	0.07409	0.1857
Q1,Q3	P-value	<1e-5	<1e-5
Q2,Q3	Variance Among	3.96	8.12
Q2,Q3	Variance Within	96.04	91.88
Q2,Q3	F_{st}	0.03958	0.08118
Q2,Q3	P-value	0.01662	0.02737

[1] In order to confirm qualitative differences in the distribution of haplogroups between ChrY and MtDNA across Qatari populations, an analysis of molecular variance (AMOVA) (Excoffier 1992) was conducted using Arlequin (Excoffier and Lischer 2010). For each of 47 ChrY sequences and 96 MtDNA sequences in Q1 (Bedouin), Q2 (Perisan-South Asian), and Q3 (African) populations, the VCF file of genotypes for segregating sites (see Supplemental Table II) was converted into Arlequin input format using a Python script and then imported into Arlequin for analysis. The AMOVA variance components analysis and F_{st} were calculated for a three-way comparison of the Qatari subpopulations and for all 2-way comparisons. Shown are the comparison, the statistic name, and the statistic result in MtDNA and ChrY.

Supplemental Table VIII. X/A Diversity[1]

	Genome-wide			Far from genes (7th bin)		
**Population**	**A**	**X**	**X/A**	**A**	**X**	**X/A**
**Q1 (Bedouin)**	0.012688 (0.000048)	0.008776 (0.000158)	0.691717 (0.012727)	0.013115 (0.000107)	0.010880 (0.000419)	0.829604 (0.032812)
**Q2 (Persian-South Asian)**	0.012996 (0.000050)	0.008967 (0.000172)	0.689991 (0.013468)	0.013456 (0.000109)	0.011138 (0.000422)	0.827715 (0.032190)
**Q3 (African)**	0.015470 (0.000050)	0.012254 (0.000178)	0.792088 (0.011778)	0.015970 (0.000108)	0.014617 (0.000456)	0.915260 (0.029245)
**QG**	0.013524 (0.000048)	0.009815 (0.000159)	0.725777 (0.012050)	0.013981 (0.000104)	0.012017 (0.000411)	0.859469 (0.030203)
**YRI**	0.016012 (0.000048)	0.012219 (0.000173)	0.763118 (0.011047)	0.016505 (0.000103)	0.014166 (0.000401)	0.858246 (0.024923)
**CEU**	0.011984 (0.000047)	0.007643 (0.000159)	0.637753 (0.013527)	0.012426 (0.000105)	0.009577 (0.000414)	0.770754 (0.034074)
**CHB**	0.011217 (0.000047)	0.006757 (0.000163)	0.602420 (0.014766)	0.011587 (0.000103)	0.008365 (0.000428)	0.721938 (0.037635)
**JPT**	0.011226 (0.000047)	0.006628 (0.000167)	0.590412 (0.015087)	0.011619 (0.000103)	0.007833 (0.000442)	0.674156 (0.038679)
**TSI**	0.012079 (0.000047)	0.007704 (0.000160)	0.637825 (0.013477)	0.012523 (0.000107)	0.009686 (0.000417)	0.773457 (0.034022)
**LWK**	0.016153 (0.000048)	0.012437 (0.000172)	0.769934 (0.010852)	0.016652 (0.000105)	0.014359 (0.000411)	0.862297 (0.025306)
**IBS**	0.012145 (0.000050)	0.007658 (0.000168)	0.630543 (0.014057)	0.012533 (0.000112)	0.009429 (0.000406)	0.752313 (0.033175)
**GBR**	0.012002 (0.000047)	0.007641 (0.000158)	0.636613 (0.013380)	0.012428 (0.000107)	0.009693 (0.000419)	0.779893 (0.034461)
**FIN**	0.011962 (0.000047)	0.007597 (0.000159)	0.635096 (0.013501)	0.012378 (0.000104)	0.009441 (0.000395)	0.762694 (0.032644)
**PUR**	0.013177 (0.000048)	0.009208 (0.000156)	0.698758 (0.012083)	0.013625 (0.000104)	0.011037 (0.000390)	0.810050 (0.029372)
**MXL**	0.012328 (0.000047)	0.007873 (0.000152)	0.638616 (0.012524)	0.012717 (0.000102)	0.009497 (0.000427)	0.746767 (0.034239)
**CLM**	0.012918 (0.000047)	0.008491 (0.000158)	0.657302 (0.012487)	0.013396 (0.000102)	0.010112 (0.000411)	0.754860 (0.031302)
**CHS**	0.011185 (0.000047)	0.006788 (0.000164)	0.606882 (0.014920)	0.011577 (0.000103)	0.008302 (0.000448)	0.717144 (0.039324)
**ASW**	0.015905 (0.000049)	0.012189 (0.000166)	0.766357 (0.010678)	0.016397 (0.000105)	0.014161 (0.000391)	0.863653 (0.024529)

[1] A total of 43,999,783 bp of X-linked and 695,776,796 bp of autosomal sequence were used to estimate X/A diversity in each population, after application of filters detailed in the Supplemental Methods. SNP variants within these regions included 133,713 ChrX SNPs and 3,284,862 autosomal SNPs segregating in both Qatari and 1000 Genomes (The 1000 Genomes Project Consortium 2012) females. Estimates of nucleotide diversity (the average number of pairwise differences per base between all haploid samples in a population) normalized by human-macaque divergence (the proportion of differences between the human and macaque reference after Jukes-Cantor correction) were estimated over 100 kbp loci obtained by grouping bases along chromosomes that remained after filtering. Shown are the normalized (absolute) diversity estimates (with standard error in parentheses) for individual populations in the autosomes, ChrX, and the ratio X/A (Gottipati 2011; Arbiza 2014). Population codes for 1000 Genomes are described in Supplemental Table IV.

Supplemental Table IX. Relative ratio X/A Diversity[1]

Population #1	Population # 2	Ratio	Relative X/A	P value
**Q1 (Bedouin)**	LWK	Q1/LWK	0.8984 (0.0116)	*
	YRI	Q1/YRI	0.9064 (0.0118)	*
	ASW	Q1/ASW	0.9026 (0.0103)	*
**Q2 (Persian-South Asian)**	LWK	Q2/LWK	0.8962 (0.0123)	*
	YRI	Q2/YRI	0.9042 (0.0125)	*
	ASW	Q2/ASW	0.9004 (0.0110)	*
**Q3 (African)**	LWK	LWK/Q3	0.9720 (0.0064)	*
	YRI	YRI/Q3	0.9634 (0.0065)	*
	ASW	ASW/Q3	0.9675 (0.0056)	*

[1] Shown are the relative X-linked to autosomal (X/A) diversity ratios (Gottipati 2011; Arbiza 2014) of Qatari and African populations. Relative X/A diversity is X/A in one group divided by X/A in the other. Ratios are always shown for the population with the lower diversity. The p value is for a two-tailed two sample z-test between the distributions of absolute X/A in each of the populations compared obtained from 10,000 moving block bootstrap samples. Shown are the populations in the comparison, ratio calculated, observed ratio (standard error in parentheses) and p value (* indicates $p<0.0001$).

Supplemental Table X. ALDER Results for Supplemental Figure 8[1]

			African admixture			
			Generations ago		Percent	
Study	Region	Population	#	Error (+/-)	%	Error (+/-)
Human Origins	Africa	AA Denver	20.91	6.92	60.50	2.30
Qatari genomes	Middle East	Q3 (African)	9.32	0.69	37.60	0.90
Human Origins	Africa	Biaka Pygmy	30.84	3.55	20.50	1.70
Human Origins	Africa	Khomani	11.22	1.67	17.80	0.80
Human Origins	Africa	Mandenka	13.85	6.13	16.00	4.70
Human Origins	Africa	Hadza Henn	9.40	2.92	15.40	1.90
Human Origins	Africa	Mbuti Pygmy	28.26	3.88	14.40	1.10
Qatari genomes	Middle East	Q0 (Subpopulation Unassigned)	12.34	1.75	11.10	0.80
Human Origins	Middle East	Egyptian Metspalu	26.03	4.64	8.20	0.50
Human Origins	Middle East	Yemen	12.43	3.25	7.60	0.60
Human Origins	Africa	Mozabite	23.26	3.07	7.50	0.50
Human Origins	Middle East	Egyptian Comas	32.27	5.10	6.70	0.70
Human Origins	Africa	Masai Kinyawa MKK	7.77	3.18	6.60	1.50
Human Origins	Africa	Ju-hoan North	49.44	11.99	6.60	1.00
Human Origins	Africa	Tunisian	17.64	4.74	6.10	0.80
Human Origins	Africa	Saharawi	10.34	4.61	5.60	1.20
Human Origins	Middle East	Bedouin A	28.68	2.58	5.40	0.30
Human Origins	Africa	Algerian	63.77	16.69	5.00	1.40
Qatari genomes	Middle East	Q2 (Persian-South Asian)	13.99	1.41	5.00	0.30
Human Origins	West Eurasia	Jordanian	35.10	6.70	4.40	0.50
Human Origins	Africa	Somali	17.62	5.50	4.20	0.80
Human Origins	Middle East	Syrian	32.34	7.34	4.10	0.50
Human Origins	Middle East	Palestinian	30.68	2.22	3.90	0.30
Human Origins	Middle East	Bedouin B	25.49	3.16	3.60	0.20
Human Origins	America	Quechua Coriell	10.02	2.54	3.30	0.40
Human Origins	South Asia	Makrani	22.78	3.09	3.30	0.30
Human Origins	America	Mayan	11.41	0.81	3.20	0.20
Human Origins	Middle East	Saudi	37.18	8.35	2.70	0.40
Qatari genomes	Middle East	Q1 (Bedouin)	15.16	1.37	2.60	0.10
Human Origins	Middle East	Lebanese	30.50	6.27	2.40	0.50
Human Origins	Oceania	Bougainville	40.84	8.74	2.10	0.40
Human Origins	West Eurasia	Maltese	36.69	10.53	2.00	0.40
Human Origins	America	Mixtec	13.55	2.08	2.00	0.30
Human Origins	America	Surui	11.90	2.16	1.70	0.30
Human Origins	Central Asia Siberia	Selkup	23.25	7.04	1.50	0.20
Human Origins	America	Pima	7.88	1.66	1.50	0.20
Human Origins	America	Zapotec	14.15	2.76	1.50	0.20
Human Origins	Central Asia Siberia	Aleut	16.89	4.67	1.40	0.40
Human Origins	South Asia	Brahui	24.58	4.70	1.40	0.20
Human Origins	Middle East	Druze	36.51	5.90	1.40	0.20
Human Origins	West Eurasia	Lezgin	240.91	85.81	1.30	1.00
Human Origins	Central Asia Siberia	Kyrgyz	37.46	17.12	1.30	0.40
Human Origins	Central Asia Siberia	Yakut	15.58	2.98	1.30	0.10
Human Origins	West Eurasia	Spanish Extremadura IBS	29.53	14.26	1.20	0.50
Human Origins	West Eurasia	Turkish Adana	37.05	14.10	1.20	0.30
Human Origins	South Asia	Kalash	26.97	7.65	1.20	0.20
Human Origins	Central Asia Siberia	Nganasan	34.76	6.40	1.20	0.20
Human Origins	America	Karitiana	12.87	1.67	1.20	0.10
Human Origins	South Asia	Balochi	18.36	6.03	1.10	0.20
Human Origins	South Asia	Kusunda	12.74	2.71	1.00	0.30

Supplemental Table X. ALDER results for Supplemental Figure 8[1] (continued, page 2)

			African admixture			
			Generations ago		Percent	
Study	Region	Population	#	Error (+/-)	%	Error (+/-)
Human Origins	Middle East	Iranian	15.28	5.33	1.00	0.20
Human Origins	East Asia	Lahu	16.25	5.35	1.00	0.20
Human Origins	Central Asia Siberia	Tubalar	23.87	4.02	1.00	0.10
Human Origins	East Asia	Atayal Coriell	14.87	5.49	0.90	0.20
Human Origins	Central Asia Siberia	Kalmyk	11.32	3.34	0.90	0.20
Human Origins	Central Asia Siberia	Yukagir Tundra	18.90	5.53	0.80	0.10
Human Origins	Central Asia Siberia	Chukchi	42.11	16.77	0.70	0.20
Human Origins	Central Asia Siberia	Koryak	17.14	7.02	0.70	0.10
Human Origins	Central Asia Siberia	Tuvinian	17.17	4.50	0.70	0.10
Human Origins	Central Asia Siberia	Eskimo Naukan	18.18	4.72	0.70	0.10
Human Origins	West Eurasia	Sardinian	40.55	12.27	0.60	0.20
Human Origins	America	Mixe	12.00	4.65	0.60	0.10
Human Origins	East Asia	Oroqen	27.71	12.09	0.50	0.20
Human Origins	East Asia	Tu	22.77	9.27	0.50	0.10
Human Origins	East Asia	Daur	16.86	5.65	0.50	0.10
Human Origins	East Asia	Xibo	30.58	10.83	0.40	0.20
Human Origins	West Eurasia	Abkhasian	9.57	4.08	0.40	0.10
Human Origins	Central Asia Siberia	Ulchi	16.49	3.81	0.40	0.10
Human Origins	South Asia	Pathan	44.05	19.46	0.30	0.10
Human Origins	West Eurasia	Spanish Baleares IBS	46.74	22.79	0.00	0.00

[1] In order to estimate the proportion and timing of African admixture in Qatari populations, the genomes of the 104 Qataris and the Human Origins (Lazaridis 2014) populations were analyzed using ALDER 1.2 (Loh 2013). For each population in Supplemental Table V, Yoruba is used as a reference panel, and the timing and percent of African admixture is estimated, with confidence intervals established using a block jackknife (Reich 2009). Shown are the populations where a result was obtained, based on sufficiently large population sample and low error of estimates. Populations are sorted from top to bottom by decreasing proportion of estimated African admixture. Shown are the regional origin, the population, generations to African admixture with standard error, and proportion of African admixture with standard error.

Supplemental Table XI. Neanderthal Ancestry[1]

Study	Region	Population[2]	*D-statistic*	F_4 ratio
Human Origins	Africa	Bantu SA Zulu	-0.031	-0.017
Human Origins	Africa	Bantu SA Pedi	-0.025	-0.013
Human Origins	Africa	Hadza Henn	-0.032	-0.010
Human Origins	Africa	Khomani	-0.021	-0.009
Human Origins	Africa	Biaka Pygmy	-0.028	-0.008
1000 Genomes	Africa	LWK	-0.027	-0.004
Human Origins	Africa	Gambian GWD	-0.026	-0.004
1000 Genomes	Africa	YRI	-0.027	-0.003
Human Origins	Africa	Mende Sierra Leone MSL	-0.025	-0.003
Human Origins	Africa	Ju hoan North	-0.017	-0.003
Human Origins	Africa	Bantu SA Tswana	-0.025	-0.003
Human Origins	Africa	Luhya Kenya LWK	-0.026	-0.002
Human Origins	Africa	Luo	-0.025	0.000
Human Origins	Africa	AA Denver	-0.023	0.002
Human Origins	Africa	Masai Kinyawa MKK	-0.022	0.002
Human Origins	Africa	Mandenka	-0.025	0.003
Human Origins	Africa	Bantu Kenya	-0.024	0.005
Human Origins	Africa	Bantu SA Herero	-0.024	0.005
Human Origins	Africa	Kikuyu	-0.023	0.005
Human Origins	Africa	Datog	-0.023	0.006
Human Origins	Africa	Mbuti Pygmy	-0.023	0.007
Human Origins	Africa	Esan Nigeria ESN	-0.022	0.008
1000 Genomes	Africa	ASW	-0.019	0.008
Human Origins	Africa	Bantu SA S Sotho	-0.020	0.009
Human Origins	Africa	Masai Ayodo	-0.022	0.009
Human Origins	Africa	Somali	-0.018	0.010
Qatari genomes	Q3 African	Q3 African	-0.016	0.013
Human Origins	Africa	Algerian	-0.005	0.014
Human Origins	Middle East	Egyptian Metspalu	-0.004	0.015
Human Origins	America	Bolivian Cochabamba	0.001	0.015
Human Origins	Central Asia Siberia	Chukchi Sir	0.002	0.015
Human Origins	Africa	Tunisian	-0.004	0.016
Human Origins	Central Asia Siberia	Chukchi Reindeer	0.004	0.016
Human Origins	America	Mixe	0.005	0.017
Human Origins	Middle East	Yemen	-0.006	0.018
Human Origins	West Eurasia	Spanish Canarias IBS	0.004	0.018
Human Origins	South Asia	Gujarati A GIH	-0.001	0.019
Human Origins	Middle East	Syrian	-0.003	0.020
Human Origins	America	Zapotec	0.005	0.020
Human Origins	South Asia	Gujarati B GIH	-0.001	0.021
Human Origins	Middle East	Iranian	0.001	0.021
Human Origins	West Eurasia	Italian South	0.005	0.021
Human Origins	East Asia	Oroqen	0.002	0.021
Human Origins	Middle East	Turkish Trabzon	0.001	0.021
Human Origins	South Asia	Balochi	0.001	0.022
Human Origins	Africa	Mozabite	-0.003	0.022
Human Origins	Middle East	Jordanian	-0.001	0.022

Supplemental Table XI. Neanderthal Ancestry[1] (continued, page 2)

Study	Region	Population[2]	*D-statistic*	F_4 ratio
Human Origins	South Asia	Makrani	0.002	0.022
Human Origins	Middle East	Bedouin B	-0.003	0.022
Human Origins	Middle East	Druze	-0.003	0.022
Human Origins	Africa	Saharawi	-0.005	0.023
Human Origins	West Eurasia	Croatian	0.005	0.023
Human Origins	Middle East	Palestinian	-0.001	0.023
Human Origins	America	Quechua Coriell	0.005	0.023
1000 Genomes	Europe	IBS	0.007	0.023
Human Origins	West Eurasia	Spanish Cantabria IBS	0.001	0.023
Human Origins	Middle East	Bedouin A	-0.003	0.024
Human Origins	West Eurasia	Greek Comas	0.006	0.024
Human Origins	West Eurasia	Kumyk	0.003	0.024
Human Origins	South Asia	Bengali Bangladesh BEB	0.004	0.024
Human Origins	Africa	Bantu SA Ovambo	-0.018	0.024
Qatari genomes	Q2 (Persian)	Q2 (Persian-South Asian)	-0.003	0.024
Human Origins	East Asia	Atayal Coriell	0.006	0.024
Human Origins	Central Asia Siberia	Koryak	0.003	0.024
Human Origins	Central Asia Siberia	Dolgan	0.005	0.024
Human Origins	South Asia	Brahui	0.002	0.024
Human Origins	West Eurasia	Spanish Baleares IBS	0.003	0.024
Human Origins	America	Piapoco	0.001	0.025
Human Origins	America	Mayan	0.005	0.025
Human Origins	Middle East	Egyptian Comas	-0.005	0.025
Human Origins	Middle East	Turkish Balikesir	0.004	0.025
Human Origins	West Eurasia	Spanish Galicia IBS	0.004	0.025
Human Origins	South Asia	Punjabi Lahore PJL	0.002	0.025
Human Origins	West Eurasia	Bulgarian	0.006	0.025
Human Origins	South Asia	Burusho	0.003	0.025
Human Origins	East Asia	Ami Coriell	0.006	0.025
Human Origins	Central Asia Siberia	Yukagir Forest	0.008	0.026
Human Origins	West Eurasia	Sardinian	0.006	0.026
1000 Genomes	America	MXL	0.004	0.026
Human Origins	Central Asia Siberia	Uzbek	0.004	0.026
Human Origins	West Eurasia	Armenian	0.004	0.026
Human Origins	West Eurasia	Hungarian Coriell	0.005	0.026
Human Origins	Middle East	Turkish	0.007	0.026
Qatari genomes	Q1 (Bedouin)	Q1 (Bedouin)	0.000	0.026
Human Origins	Middle East	Turkish Kayseri	0.003	0.026
Human Origins	Middle East	Saudi	-0.001	0.026
Human Origins	West Eurasia	Adygei	0.005	0.026
Human Origins	West Eurasia	French South	0.008	0.026
Human Origins	America	Pima	0.004	0.026
Human Origins	East Asia	Han NChina	0.008	0.026
Human Origins	Central Asia Siberia	Itelmen	0.005	0.027
Human Origins	East Asia	Uygur	0.004	0.027
1000 Genomes	America	PUR	0.000	0.027
Human Origins	Central Asia Siberia	Eskimo Chaplin	0.007	0.027
Human Origins	West Eurasia	Abkhasian	0.003	0.027
Human Origins	Central Asia Siberia	Altaian	0.006	0.027

Supplemental Table XI. Neanderthal Ancestry[1] (continued, page 3)

Study	Region	Population[2]	*D-statistic*	F_4 ratio
Human Origins	Central Asia Siberia	Tubalar	0.005	0.027
Human Origins	Central Asia Siberia	Mongola	0.001	0.027
Human Origins	America	Surui	0.009	0.027
Human Origins	Central Asia Siberia	Chukchi	0.005	0.027
Human Origins	South Asia	Kalash	0.004	0.028
Human Origins	South Asia	Gujarati D GIH	0.007	0.028
Human Origins	Central Asia Siberia	Aleut	0.007	0.028
Human Origins	West Eurasia	Spanish Pais Vasco IBS	0.009	0.028
Human Origins	Central Asia Siberia	Kalmyk	0.006	0.028
Human Origins	West Eurasia	Italian West Sicilian	0.001	0.028
Human Origins	Middle East	Lebanese	-0.004	0.028
Human Origins	West Eurasia	Nogai	0.002	0.028
Human Origins	West Eurasia	Icelandic	0.005	0.028
Human Origins	East Asia	Korean	0.007	0.028
Human Origins	West Eurasia	Italian Bergamo	0.003	0.029
Human Origins	West Eurasia	North Ossetian	0.004	0.029
Human Origins	Middle East	Turkish Istanbul	0.001	0.029
Human Origins	West Eurasia	Belarusian	0.009	0.029
Human Origins	West Eurasia	Finnish FIN	0.005	0.029
Human Origins	Central Asia Siberia	Even	0.004	0.029
Human Origins	West Eurasia	Cypriot	0.001	0.029
Human Origins	East Asia	Cambodian	0.004	0.029
1000 Genomes	America	CLM	0.004	0.029
Human Origins	West Eurasia	Hungarian Metspalu	0.007	0.029
Human Origins	Middle East	Turkish Aydin	0.000	0.029
Human Origins	West Eurasia	English Kent GBR	0.004	0.029
Human Origins	Middle East	Turkish Adana	0.006	0.029
Human Origins	America	Mixtec	0.006	0.029
Human Origins	West Eurasia	Spanish Cataluna IBS	0.003	0.030
1000 Genomes	Europe	GBR	0.006	0.030
Human Origins	Central Asia Siberia	Ulchi	0.006	0.030
Human Origins	West Eurasia	Georgian Megrels	0.004	0.030
Human Origins	East Asia	Tujia	0.007	0.030
Human Origins	America	Bolivian La Paz	0.007	0.030
Human Origins	West Eurasia	Basque French	0.004	0.030
Human Origins	West Eurasia	Lezgin	0.001	0.030
Human Origins	West Eurasia	French	0.006	0.030
Human Origins	Central Asia Siberia	Yukagir Tundra	0.007	0.030
Human Origins	East Asia	Yi	0.008	0.030
Human Origins	Central Asia Siberia	Yakut	0.005	0.030
Human Origins	South Asia	Hazara	0.005	0.030
Human Origins	West Eurasia	Ukrainian West	0.007	0.030
Human Origins	West Eurasia	Saami WGA	0.007	0.030
Human Origins	Central Asia Siberia	Eskimo Naukan	0.004	0.030
Human Origins	East Asia	Hezhen	0.005	0.030
Human Origins	South Asia	Pathan	0.004	0.031
Human Origins	West Eurasia	Spanish Murcia IBS	0.005	0.031
Human Origins	East Asia	Lahu	0.004	0.031
Human Origins	Central Asia Siberia	Eskimo Sireniki	0.002	0.031

Supplemental Table XI. Neanderthal Ancestry[1] (continued, page 4)

Study	Region	Population[2]	*D-statistic*	F_4 ratio
Human Origins	East Asia	Xibo	0.006	0.031
1000 Genomes	Europe	TSI	0.005	0.031
Human Origins	West Eurasia	Maltese	0.003	0.031
Human Origins	Central Asia Siberia	Tajik Pomiri	0.003	0.031
Human Origins	Central Asia Siberia	Turkmen	0.003	0.031
Human Origins	America	Bolivian Pando	0.009	0.031
Human Origins	East Asia	Tu	0.005	0.031
1000 Genomes	Europe	CEU	0.006	0.031
Human Origins	West Eurasia	Spanish Valencia IBS	0.007	0.031
Human Origins	West Eurasia	Greek Coriell	0.003	0.031
Human Origins	West Eurasia	Spanish Castilla y Leon IBS	0.002	0.032
Human Origins	West Eurasia	Russian	0.009	0.032
Human Origins	West Eurasia	Norwegian	0.004	0.032
Human Origins	West Eurasia	Basque Spanish	0.007	0.032
Human Origins	West Eurasia	Scottish Argyll Bute GBR	0.010	0.032
Human Origins	Central Asia Siberia	Nganasan	0.004	0.032
Human Origins	West Eurasia	Orcadian	0.006	0.032
1000 Genomes	Europe	FIN	0.007	0.032
Human Origins	East Asia	Kinh Vietnam KHV	0.007	0.033
Human Origins	West Eurasia	Balkar	0.007	0.033
Human Origins	West Eurasia	Spanish Aragon IBS	0.007	0.033
Human Origins	Central Asia Siberia	Selkup	0.003	0.033
1000 Genomes	East Asia	JPT	0.007	0.033
Human Origins	West Eurasia	English Cornwall GBR	0.005	0.033
Human Origins	West Eurasia	Lithuanian	0.005	0.033
Human Origins	South Asia	Gujarati C GIH	0.008	0.034
Human Origins	West Eurasia	Estonian	0.009	0.034
Human Origins	West Eurasia	Chechen	0.006	0.034
Human Origins	East Asia	She	0.007	0.034
Human Origins	East Asia	Dai	0.005	0.034
1000 Genomes	East Asia	CHS	0.007	0.034
Human Origins	South Asia	Sindhi	0.005	0.034
Human Origins	East Asia	Thai	0.008	0.034
1000 Genomes	East Asia	CHB	0.008	0.034
Human Origins	East Asia	Japanese	0.008	0.034
Human Origins	West Eurasia	Chuvash	0.008	0.034
Human Origins	East Asia	Han	0.007	0.034
Human Origins	South Asia	Kusunda	0.008	0.034
Human Origins	West Eurasia	Spanish Extremadura IBS	0.005	0.034
Human Origins	Central Asia Siberia	Kyrgyz	0.005	0.034
Human Origins	Central Asia Siberia	Tlingit	0.005	0.035
Human Origins	Central Asia Siberia	Tuvinian	0.007	0.036
Human Origins	West Eurasia	Czech	0.008	0.036
Human Origins	West Eurasia	Spanish Castilla la Mancha IBS	0.008	0.036
Human Origins	America	Karitiana	0.010	0.036
Human Origins	West Eurasia	Mordovian	0.008	0.036
Human Origins	East Asia	Daur	0.005	0.036
Human Origins	West Eurasia	Spanish Andalucia IBS	0.003	0.037
Human Origins	East Asia	Miao	0.010	0.037

Supplemental Table XI. Neanderthal Ancestry[1] (continued, page 5)

Study	Region	Population[2]	*D-statistic*	F_4 ratio
Human Origins	West Eurasia	Ukrainian East	0.011	0.037
Human Origins	Central Asia Siberia	Mansi	0.005	0.037
Human Origins	West Eurasia	Italian Tuscan	0.009	0.038
Human Origins	West Eurasia	Albanian	0.006	0.039
Human Origins	West Eurasia	Italian East Sicilian	0.006	0.041
Human Origins	East Asia	Naxi	0.004	0.041
Human Origins	Oceania	Bougainville	0.018	0.097
Human Origins	Oceania	Papuan	0.014	0.106
Human Origins	Oceania	Australian ECCAC	0.020	0.115

[1] Neanderthal ancestry was calculated in world populations from the combined dataset of Qatari genomes, 1000 Genomes, and Human Origins using two methods, Patterson's *D-statistic* and F_4 ratio estimation, both implemented in the AdmixTools 3.0 package (Patterson 2012). While the *D-statistic* estimates relative Neanderthal ancestry compared to another population (in this case Q1), the F_4 ratio estimates α, the Neanderthal ancestry proportion in a population. Shown are the results for both statistics for all populations in the combined dataset, including 1000 Genomes, Africa; 1000 Genomes, America; 1000 Genomes, East Asia, 1000 Genomes, Europe, Human Origins, Africa; Human Origins, America; Human Origins, Central Asia/Siberia; Human Origins, East Asia; Human Origins, Oceania; Human Origins, South Asia; Human Origins, West Eurasia), Middle Eastern populations (Human Origins), Q1 (Bedouin), Q2 (Persian-South Asian) and Q3 (African). Results are sorted in order of increasing values of Neanderthal ancestry (F_4 ratio).

[2] In certain cases individuals from the 1000 Genomes Project (The 1000 Genomes Project Consortium 2012) are present in the Human Origins (Lazaridis 2014) dataset. For these populations, a separate *D-statistic* and F_4 ratio is calculated for the individuals from 1000 Genomes of the same population not present in the Human Origins dataset. For example, in this table "Yoruba" indicates Yoruba individuals present in the Human Origins dataset, including individuals duplicated in 1000 Genomes. The remaining 1000 Genomes Yoruba are in the "YRI" population sample. For all 1000 Genomes populations, when the population label is a 3-letter abbreviation (such as "YRI"), this indicates the 1000 Genomes sample not present in Human Origins.

References

Abecasis, G.R., Auton, A., Brooks, L.D., DePristo, M.A., Durbin, R.M., Handsaker, R.E., Kang, H.M., Marth, G.T., and McVean, G.A. 2012. An integrated map of genetic variation from 1,092 human genomes. *Nature* **491**:56-65

Alexander, D.H., Novembre, J., and Lange, K. 2009. Fast model-based estimation of ancestry in unrelated individuals. *Genome Res* **19**:1655-1664

Anderson, S., Bankier, A.T., Barrell, B.G., de Bruijn, M.H., Coulson, A.R., Drouin, J., Eperon, I.C., Nierlich, D.P., Roe, B.A., Sanger, F., et al. 1981. Sequence and organization of the human mitochondrial genome. *Nature* **290**:457-465

Arbiza, L., Gottipati, S., Siepel, A., and Keinan, A. 2014. Contrasting X-linked and autosomal diversity across 14 human populations. *Am J Hum Genet* **94**:827-844

Bentley, D.R., Balasubramanian, S., Swerdlow, H.P., Smith, G.P., Milton, J., Brown, C.G., Hall, K.P., Evers, D.J., Barnes, C.L., Bignell, H.R., et al. 2008. Accurate whole human genome sequencing using reversible terminator chemistry. *Nature* **456**:53-59

Chang, C.C., Chow, C.C., Tellier, L.C., Vattikuti, S., Purcell, S.M., and Lee, J.J. 2015. Second-generation PLINK: rising to the challenge of larger and richer datasets. *Gigascience.* **4**:7

Delaneau, O., Zagury, J.F., and Marchini, J. 2013. Improved whole-chromosome phasing for disease and population genetic studies. *Nat.Methods* **10**:5-6

DePristo, M.A., Banks, E., Poplin, R., Garimella, K.V., Maguire, J.R., Hartl, C., Philippakis, A.A., del, A.G., Rivas, M.A., Hanna, M., et al. 2011. A framework for variation discovery and genotyping using next-generation DNA sequencing data. *Nat.Genet.* **43**:491-498

Excoffier, L. and Lischer, H.E. 2010. Arlequin suite ver 3.5: a new series of programs to perform population genetics analyses under Linux and Windows. *Mol Ecol.Resour.* **10**:564-567

Excoffier, L., Smouse, P.E., and Quattro, J.M. 1992. Analysis of molecular variance inferred from metric distances among DNA haplotypes: application to human mitochondrial DNA restriction data. *Genetics* **131**:479-491

Gottipati, S., Arbiza, L., Siepel, A., Clark, A.G., and Keinan, A. 2011. Analyses of X-linked and autosomal genetic variation in population-scale whole genome sequencing. *Nat Genet* **43**:741-743

Hunter-Zinck, H., Musharoff, S., Salit, J., Al-Ali, K.A., Chouchane, L., Gohar, A., Matthews, R., Butler, M.W., Fuller, J., Hackett, N.R., et al. 2010. Population genetic structure of the people of Qatar. *Am.J.Hum.Genet.* **87**:17-25

International Diabetes Federationhttp://www.idf.org/sites/default/files/DA-regional-factsheets-2014_FINAL.pdf; IDF Diabetes Atlas 6th edition.

Keinan, A., Mullikin, J.C., Patterson, N., and Reich, D. 2007. Measurement of the human allele frequency spectrum demonstrates greater genetic drift in East Asians than in Europeans. *Nat Genet* **39**:1251-1255

Kent, W.J., Sugnet, C.W., Furey, T.S., Roskin, K.M., Pringle, T.H., Zahler, A.M., and Haussler, D. 2002. The human genome browser at UCSC. *Genome Res* **12**:996-1006

Lahiri, S.N. 2003. *Resampling methods for dependent data*. Springer, New York.

lattice: Trellis Graphics for R.

Lazaridis, I., Patterson, N., Mittnik, A., Renaud, G., Mallick, S., Kirsanow, K., Sudmant, P.H., Schraiber, J.G., Castellano, S., Lipson, M., et al. 2014. Ancient human genomes suggest three ancestral populations for present-day Europeans. *Nature* **513**:409-413

Li, H. and Durbin, R. 2009. Fast and accurate short read alignment with Burrows-Wheeler transform. *Bioinformatics.* **25**:1754-1760

Li, H. and Durbin, R. 2011. Inference of human population history from individual whole-genome sequences. *Nature* **475**:493-496

Li, H., Handsaker, B., Wysoker, A., Fennell, T., Ruan, J., Homer, N., Marth, G., Abecasis, G., and Durbin, R. 2009. The Sequence Alignment/Map format and SAMtools. *Bioinformatics.* **25**:2078-2079

Liu, R.Y. and Singh, K. 1992. Moving blocks jackknife and bootstrap capture weak dependence. In Exploring the limits of bootstrap (eds. R. LePage, L. Billard), pp. 225-248. John Wiley, New York.

Loh, P.R., Lipson, M., Patterson, N., Moorjani, P., Pickrell, J.K., Reich, D., and Berger, B. 2013. Inferring admixture histories of human populations using linkage disequilibrium. *Genetics* **193**:1233-1254

Manichaikul, A., Mychaleckyj, J.C., Rich, S.S., Daly, K., Sale, M., and Chen, W.M. 2010. Robust relationship inference in genome-wide association studies. *Bioinformatics.* **26**:2867-2873

McPeek, M.S. and Sun, L. 2000. Statistical tests for detection of misspecified relationships by use of genome-screen data. *Am J Hum Genet* **66**:1076-1094

Mezzavilla, M., Vozzi, D., Badii, R., Alkowari, M.K., Abdulhadi, K., Girotto, G., and Gasparini, P. 2015. Increased rate of deleterious variants in long runs of homozygosity of an inbred population from Qatar. *Hum Hered.* **79**:14-19

Omberg, L., Salit, J., Hackett, N., Fuller, J., Matthew, R., Chouchane, L., Rodriguez-Flores, J.L., Bustamante, C., Crystal, R.G., and Mezey, J.G. 2012. Inferring genome-wide patterns of admixture in Qataris using fifty-five ancestral populations. *BMC.Genet.* **13**:49

Patterson, N., Moorjani, P., Luo, Y., Mallick, S., Rohland, N., Zhan, Y., Genschoreck, T., Webster, T., and Reich, D. 2012. Ancient admixture in human history. *Genetics* **192**:1065-1093

Pickrell, J.K. and Pritchard, J.K. 2012. Inference of population splits and mixtures from genome-wide allele frequency data. *PLoS.Genet.* **8**:e1002967

Pritchard, J.K., Stephens, M., and Donnelly, P. 2000. Inference of population structure using multilocus genotype data. *Genetics* **155**:945-959

Purcell, S., Neale, B., Todd-Brown, K., Thomas, L., Ferreira, M.A., Bender, D., Maller, J., Sklar, P., de Bakker, P.I., Daly, M.J., et al. 2007. PLINK: a tool set for whole-genome association and population-based linkage analyses. *Am.J.Hum.Genet.* **81**:559-575

Python Software Foundation. www.python.org [last accessed 12/8/15]

R Core Team, R Foundation for Statistical Computing. Vienna, Austria. R: A Language and Environment for Statistical Computing. R-project.org [last accessed 12/8/15]

Rambaut, A. Molecular evolution, phylogenetics and epidemiology. http://tree.bio.ed.ac.uk/software/figtree/ [last accessed 12/8/15]

Reich, D., Thangaraj, K., Patterson, N., Price, A.L., and Singh, L. 2009. Reconstructing Indian population history. *Nature* **461**:489-494

Roach, J.C., Glusman, G., Smit, A.F., Huff, C.D., Hubley, R., Shannon, P.T., Rowen, L., Pant, K.P., Goodman, N., Bamshad, M., et al. 2010. Analysis of genetic inheritance in a family quartet by whole-genome sequencing. *Science* **328**:636-639

Rodriguez-Flores, J.L., Fakhro, K., Hackett, N.R., Salit, J., Fuller, J., gosto-Perez, F., Gharbiah, M., Malek, J.A., Zirie, M., Jayyousi, A., et al. 2014. Exome sequencing identifies potential risk variants for mendelian disorders at high prevalence in qatar. *Hum.Mutat.* **35**:105-116

Rodriguez-Flores, J.L., Fuller, J., Hackett, N.R., Salit, J., Malek, J.A., Al-Dous, E., Chouchane, L., Zirie, M., Jayoussi, A., Mahmoud, M.A., et al. 2012. Exome sequencing of only seven Qataris identifies potentially deleterious variants in the Qatari population. *PLoS.One.* **7**:e47614

Sandridge, A.L., Takeddin, J., Al-Kaabi, E., and Frances, Y. 2010. Consanguinity in Qatar: knowledge, attitude and practice in a population born between 1946 and 1991. *J Biosoc.Sci.* **42**:59-82

Stevens, E.L., Baugher, J.D., Shirley, M.D., Frelin, L.P., and Pevsner, J. 2012. Unexpected relationships and inbreeding in HapMap phase III populations. *PLoS.One.* **7**:e49575

The 1000 Genomes Project Consortium. 2012. An integrated map of genetic variation from 1,092 human genomes. *Nature* **491**:56-65

Weir, B.S. and Cockerham, C.C. 1984. Estimating F-Statistics for the analysis of population structure. *Evolution* **38**:1358-1370

Supplemental Figure Legends

Supplemental Figure 1. Map of the Arabian Peninsula, where the insert shows the nation of Qatar.

Supplemental Figure 2. STRUCTURE analysis (Pritchard 2000; Rodriguez-Flores 2012) of 100 of the original 108 Qataris that can be placed in the Q1 (Bedouin), Q2 (Persian-South Asian), or Q3 (African) subpopulations using the 48 TaqMan SNPs used for sample selection.

Supplemental Figure 3. Cryptic relatedness in the sample of 108 Qataris. Relatedness was calculated for all pairs. Each square denotes the estimated degree of relationship between two individuals in the dataset quantified using KING-robust's (Manichaikul 2010) estimated kinship coefficients for each pair of individuals. Identical individuals in red, first-degree relatives in yellow, second-degree relatives in green, third-degree relatives in blue, unrelated pairs in white. Shown is the upper triangle of the 108*108 matrix of pairwise comparisons, sorted by Qatari subpopulation: Q1 (Bedouin), Q2 (Persian-South Asian), Q3 (African), Q0 (Subpopulation Unassigned).

Supplemental Figure 4. Inbreeding coefficients. Shown is the inbreeding coefficient for 96 Q1 (Bedouin), Q2 (Persian-South Asian), or Q3 (African) Qatari (QG), Human Origins (HO), and 1000 Genomes Phase 1 (1000G-HO, duplicates in HO removed) populations, in decreasing order, as calculated by PLINK 1.9 (Chang 2015), split into three panels. At the top of each panel Q1 (Bedouin) (red), Q2 (Persian-South Asian) (dodger blue), and Q3 (African) (black) are plotted for reference. The region and name of each population is included (see Supplemental Table V for details). A. Top third of populations. B. Middle third of populations. C. Bottom third of populations.

Supplemental Figure 5. Principal components analysis (PCA) of Qatari, 1000 Genomes (minus Human Origins duplicates), and Human Origins samples. A. Shown is a plot of principal

components PC1 and PC2 of Qataris, 1000 Genomes, and Human Origins, using a pruned set of 197,714 SNPs. Shape indicates study (circle = Qatari genomes, square = 1000 Genomes, triangle = Human Origins), color coded by population. B. Shown is a plot of principal components PC1 and PC2 of Qataris and 1000 Genomes, using pruned set of 197,714 SNPs segregating in Qataris, 1000 Genomes, and Human Origins. Shape indicates study (circle = Qatari genomes, square = 1000 Genomes), and color coded by population. Population codes for 1000 Genomes (The 1000 Genomes Project Consortium 2012) are described in Supplemental Table IV.

Supplemental Figure 6. X/A Diversity. Normalized nucleotide diversity (Gottipati 2011; Arbiza 2014) was calculated both genome-wide and in loci >0.18cM from genes for the 96 Q1 (Bedouin), Q2 (Persian-South Asian), or Q3 (African) Qatari and 1000 Genomes populations (described in Supplemental Table IV), shown are the results grouped by region and population (admixed ASW, PUR, CLM, and MXL not shown). A. Autosomal (whole chromosome) B. ChrX (whole chromosome) C. X/A ratio (whole chromosome) D. Autosomal (>0.18cM from genes) E. ChrX (>0.18cM from genes) F. X/A ratio (>0.18cM from genes).

Supplemental Figure 7. Population structure. ADMIXTURE (Alexander 2009) analysis of population structure was run using a range of K from 3 to 18, with K=12 having the lowest cross-validation error (* in figure). Shown is the ancestry proportion for all runs. **A.** All populations in the combined the 104 Qatari genomes, 1000 Genomes (excluding Human Origins duplicates), and Human Origins dataset, labeled by study and region. **B.** Detail of populations from panel A having detectable ancestry in a “Bedouin” component that represents the majority of ancestry in Q1 (Bedouin), Bedouin B, and Saudi populations. **C.** Detail from panels A and B for K=12

Supplemental Figure 8. African admixture proportions. Shown in decreasing order is the percentage of African (Yoruba) admixture in world populations from Qatar and Human Origins, including populations where ALDER (Patterson 2012) did not produce an error.

Supplemental Figure 9. Tract length distributions of haplotypes assigned to African ancestry in a SupportMix (Omberg 2012) local admixture analysis. Shown is the frequency per individual haplotype (y-axis) of tract lengths assigned to 1000 Genomes African ancestry, either LWK (Luhuya in Kenya) or YRI (Yoruba in Nigeria), by SupportMix (Omberg 2012) measured in centimorgans (x-axis) as calculated using the 1000 Genomes genetic maps (Abecasis 2012) for the Q1 (Bedouin) in red, Q2 (Persian-South Asian) in dodger blue, and Q3 (African) in black when using African, Asian, or European populations of the 1000 Genomes Phase 1 as the putative ancestral groups. Given that the populations of the 1000 Genomes Phase 1 are not closely related to Middle Eastern populations, the amounts of African ancestry assignments were not consistent with previous analyses that used a more complete local sampling of the Middle East region using genotyping array data (Omberg 2012). However, the distribution provides a relative metric for assessing whether the Q1 (Bedouin) and Q2 (Persian-South Asian), which have not undergone recent admixture with African populations, show a distinctive pattern from the Q3 (African) subpopulation, where the latter are known to be recently admixed with Africans (Omberg 2012).

Supplemental Figure 10. Neanderthal ancestry in world populations. Neanderthal ancestry was calculated in world populations from the combined dataset of Qatari genomes, 1000 Genomes, and Human Origins using two methods that are highly correlated (a), F_4 ratio estimation (b) and Patterson's *D-statistic* (c), both implemented in the AdmixTools 3.0 package (Patterson 2012). The F_4 ratio estimates the Neanderthal ancestry proportion in a population, while the *D-statistic* estimates relative Neanderthal ancestry compared to another population, in this case Q1

(Bedouin). The F_4 ratio and *D-statistic* are highly correlated, as shown in panel (a), which plots the *D-statistic* (Y-axis) versus F_4 ratio (X-axis) estimates of Neanderthal ancestry. The legend in panel (a) is the same for all three panels. Shown are the results for all populations in the combined dataset, including 1000 Genomes, Africa; 1000 Genomes, America; 1000 Genomes, East Asia, 1000 Genomes, Europe, Human Origins, Africa; Human Origins, America; Human Origins, Central Asia/Siberia; Human Origins, East Asia; Human Origins, Oceania; Human Origins, South Asia; Human Origins, West Eurasia, Middle Eastern populations (Human Origins), Q1 (Bedouin), Q2 (Persian-South Asian) and Q3 (African). Results are sorted in order of increasing values of Neanderthal ancestry proportion (b) or Neanderthal ancestry relative to Q1 (Bedouin) (c). The data presented here is also available in Supplemental Table XI.

Supplemental Figure 11. TreeMix (Pickrell and Pritchard 2012) hierarchical clustering analysis of the 96 Q1 (Bedouin), Q2 (Persian-South Asian), or Q3 (African) Qatari and 1000 Genomes samples with different numbers of migration events. Shown are the maximum-likelihood trees plotted relative to the drift parameter when allowing different numbers of migration events in each analysis. **A.** The initial tree with no migration edges produced by TreeMix analysis of 11,701,491 SNPs in Qatari and 1000 Genomes populations, and inferred migration edges. Additional migration edges plotted **B.** One, **C.** Two, **D.** Three, **E.** Four, **F.** Five. TreeMix residuals also plotted for **G.** Zero, **H.** One, **I.** Two, **J.** Three, **K.** Four, **L.** Five migration edges.

Supplemental Figure 1

Arabian Peninsula

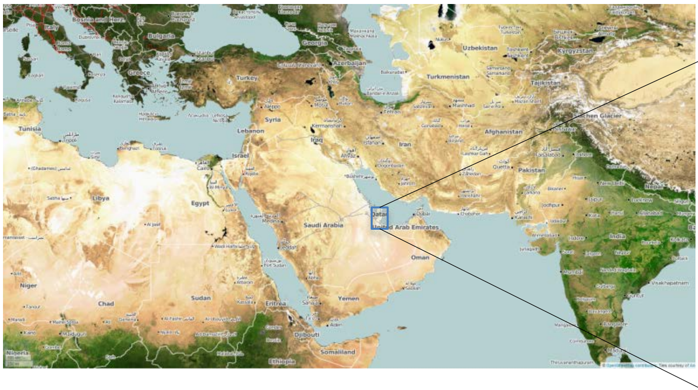

Reprinted with permission from OpenStreetMap

Qatar

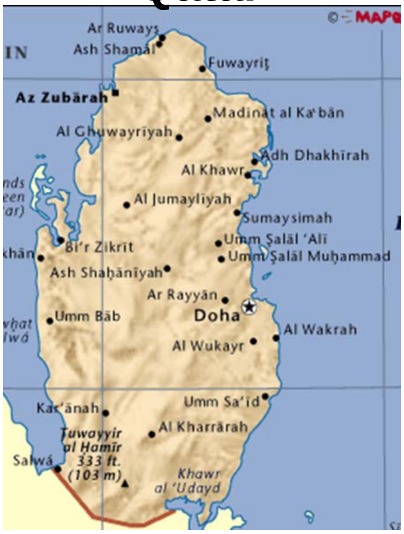

Reprinted with permission from OpenStreetMap

Supplemental Figure 2

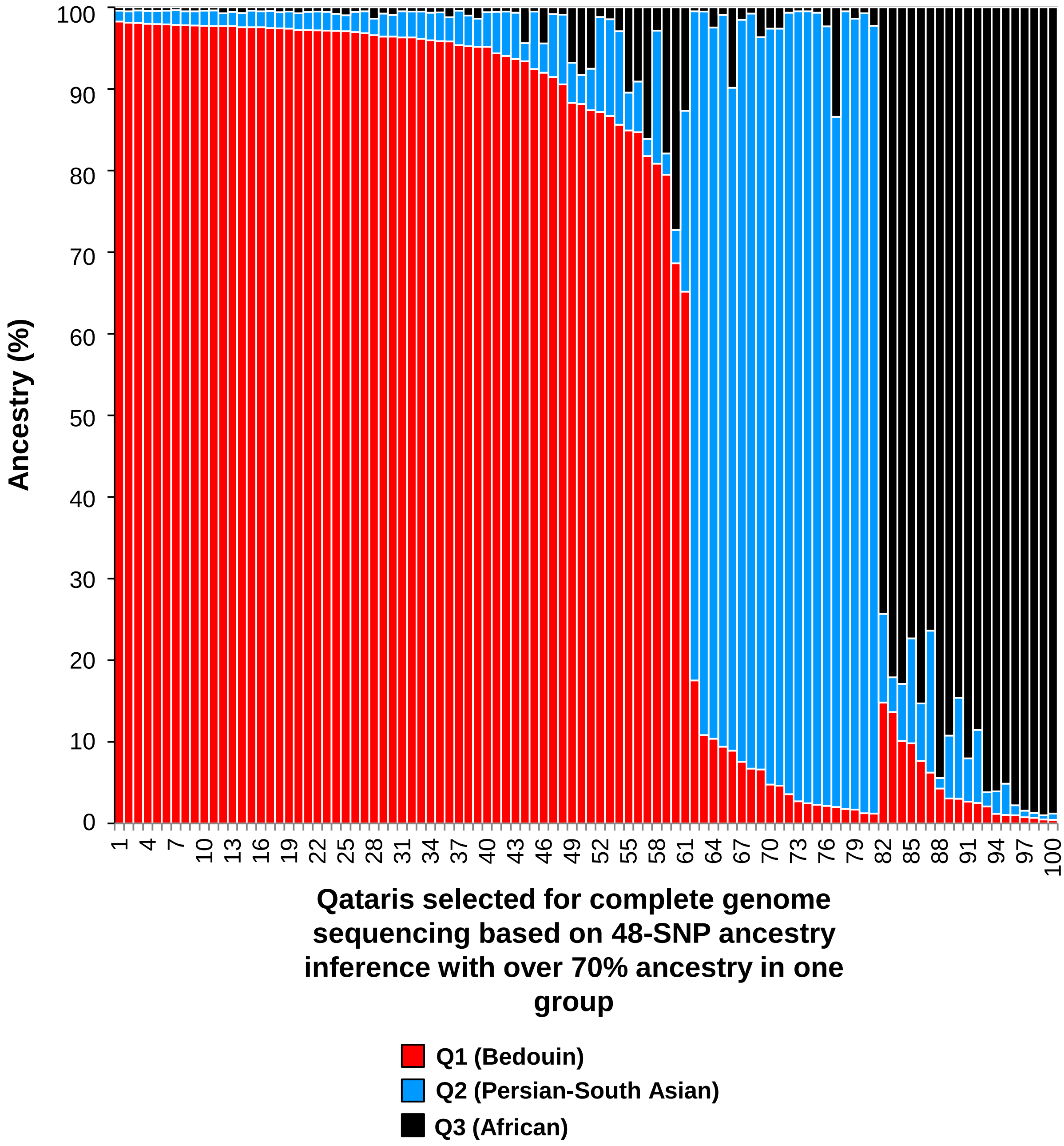

Supplemental Figure 3

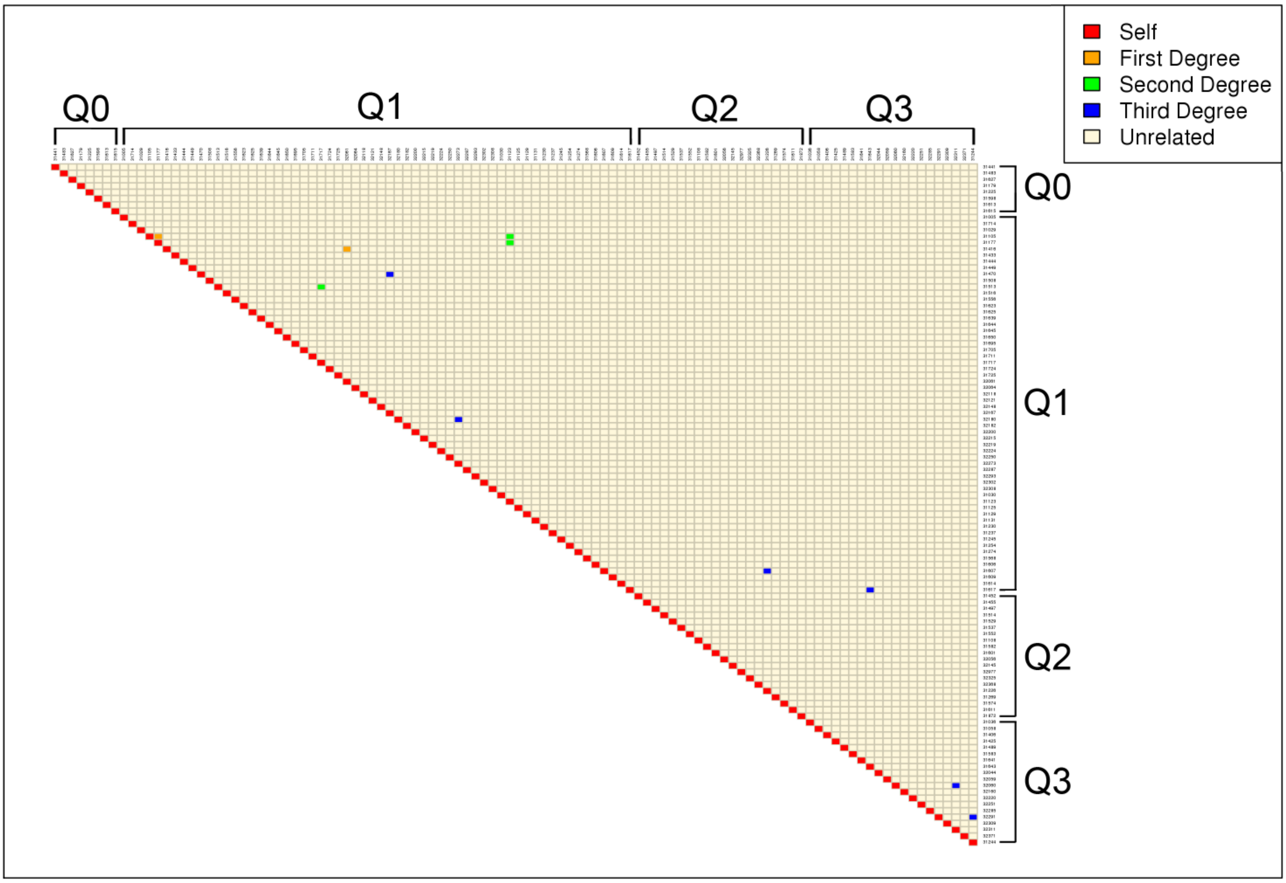

Q1 (Bedouin)
Q2 (Persian-South Asian)
Q3 (African)
Q0 (Subpopulation Unassigned)

Supplemental Figure 4

A.

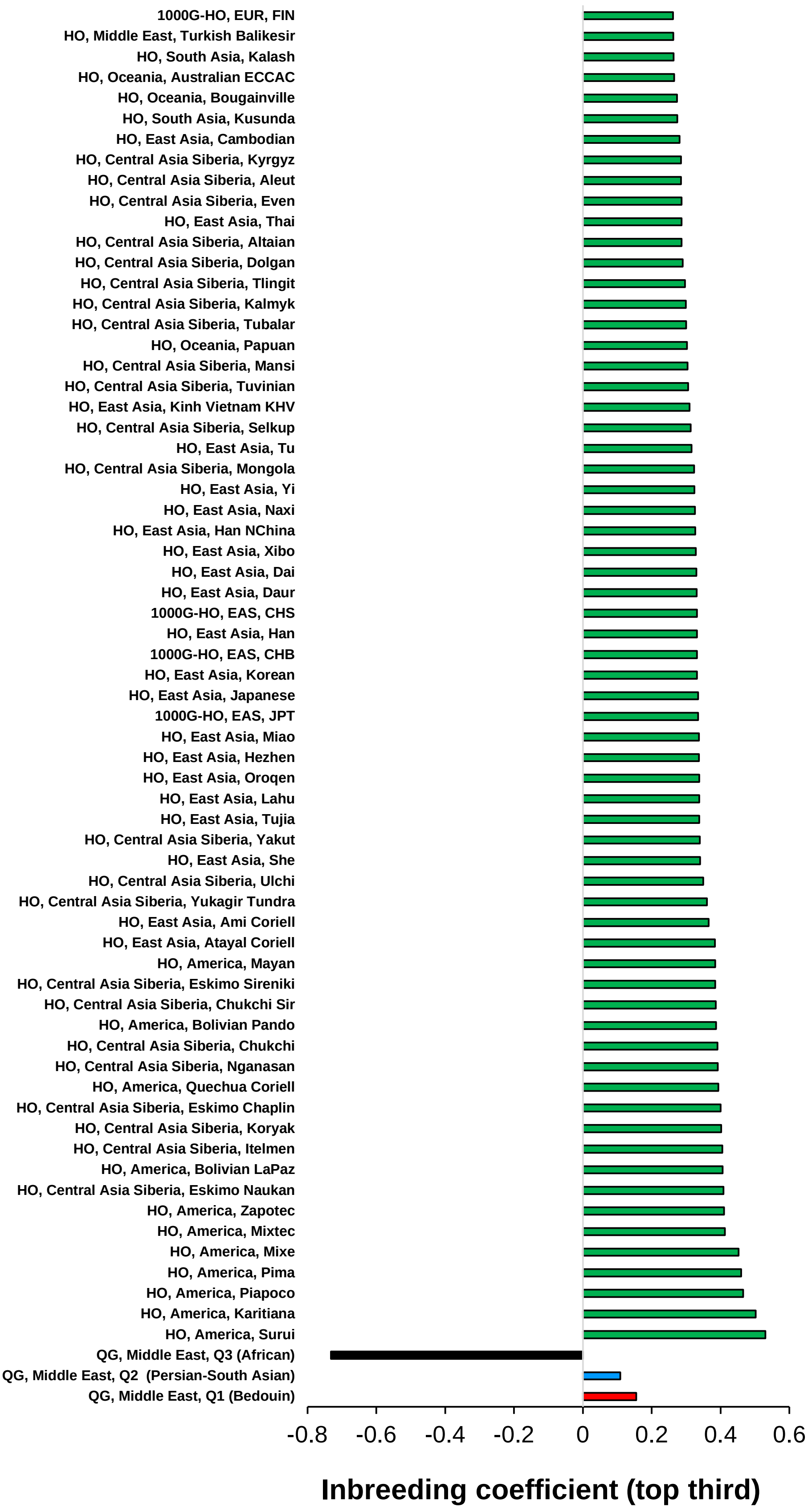

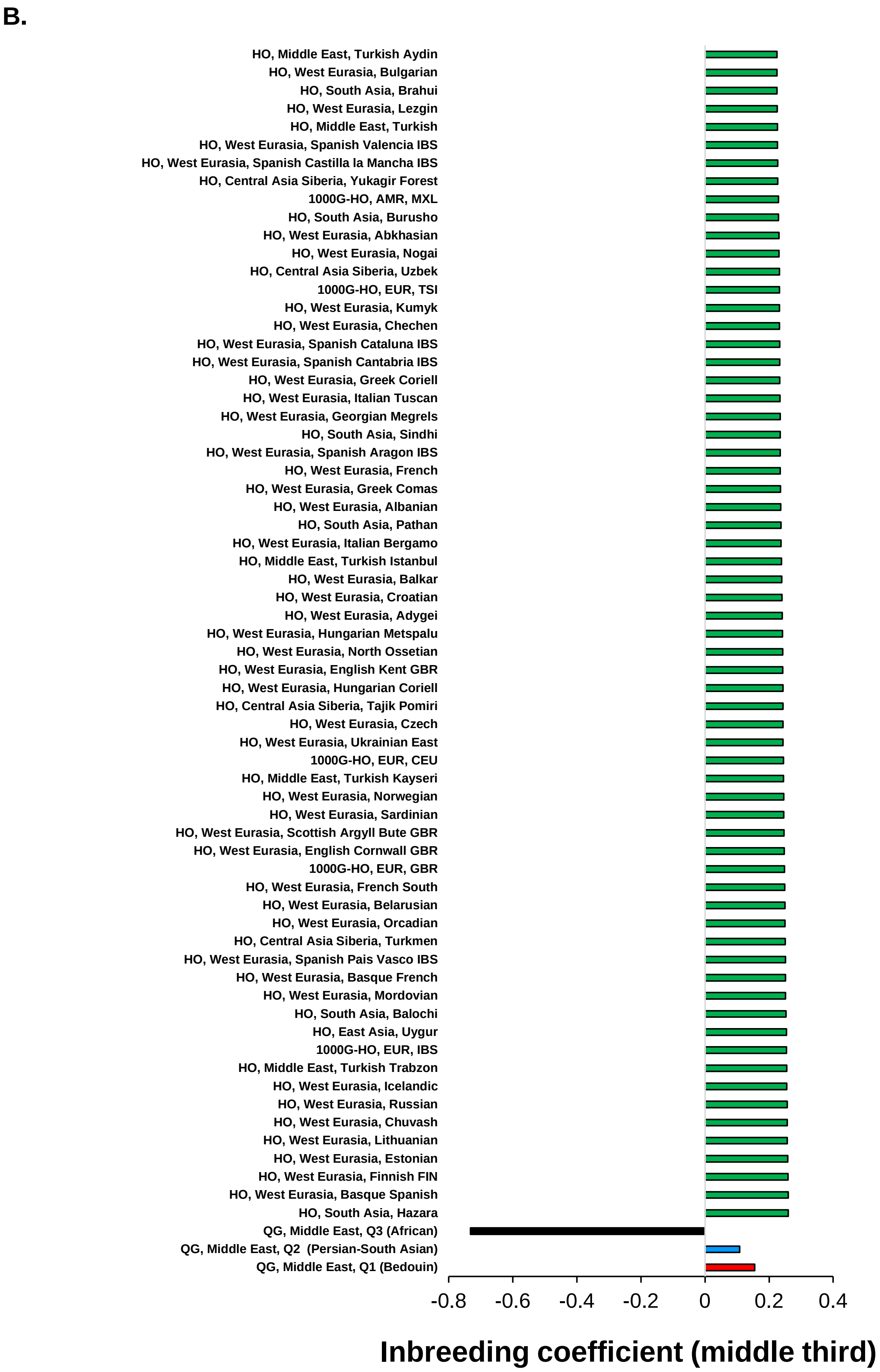
B.
HO, Middle East, Turkish Aydin
HO, West Eurasia, Bulgarian
HO, South Asia, Brahui
HO, West Eurasia, Lezgin
HO, Middle East, Turkish
HO, West Eurasia, Spanish Valencia IBS
HO, West Eurasia, Spanish Castilla la Mancha IBS
HO, Central Asia Siberia, Yukagir Forest
1000G-HO, AMR, MXL
HO, South Asia, Burusho
HO, West Eurasia, Abkhasian
HO, West Eurasia, Nogai
HO, Central Asia Siberia, Uzbek
1000G-HO, EUR, TSI
HO, West Eurasia, Kumyk
HO, West Eurasia, Chechen
HO, West Eurasia, Spanish Cataluna IBS
HO, West Eurasia, Spanish Cantabria IBS
HO, West Eurasia, Greek Coriell
HO, West Eurasia, Italian Tuscan
HO, West Eurasia, Georgian Megrels
HO, South Asia, Sindhi
HO, West Eurasia, Spanish Aragon IBS
HO, West Eurasia, French
HO, West Eurasia, Greek Comas
HO, West Eurasia, Albanian
HO, South Asia, Pathan
HO, West Eurasia, Italian Bergamo
HO, Middle East, Turkish Istanbul
HO, West Eurasia, Balkar
HO, West Eurasia, Croatian
HO, West Eurasia, Adygei
HO, West Eurasia, Hungarian Metspalu
HO, West Eurasia, North Ossetian
HO, West Eurasia, English Kent GBR
HO, West Eurasia, Hungarian Coriell
HO, Central Asia Siberia, Tajik Pomiri
HO, West Eurasia, Czech
HO, West Eurasia, Ukrainian East
1000G-HO, EUR, CEU
HO, Middle East, Turkish Kayseri
HO, West Eurasia, Norwegian
HO, West Eurasia, Sardinian
HO, West Eurasia, Scottish Argyll Bute GBR
HO, West Eurasia, English Cornwall GBR
1000G-HO, EUR, GBR
HO, West Eurasia, French South
HO, West Eurasia, Belarusian
HO, West Eurasia, Orcadian
HO, Central Asia Siberia, Turkmen
HO, West Eurasia, Spanish Pais Vasco IBS
HO, West Eurasia, Basque French
HO, West Eurasia, Mordovian
HO, South Asia, Balochi
HO, East Asia, Uygur
1000G-HO, EUR, IBS
HO, Middle East, Turkish Trabzon
HO, West Eurasia, Icelandic
HO, West Eurasia, Russian
HO, West Eurasia, Chuvash
HO, West Eurasia, Lithuanian
HO, West Eurasia, Estonian
HO, West Eurasia, Finnish FIN
HO, West Eurasia, Basque Spanish
HO, South Asia, Hazara
QG, Middle East, Q3 (African)
QG, Middle East, Q2 (Persian-South Asian)
QG, Middle East, Q1 (Bedouin)
-0.8
-0.6
-0.4
-0.2
0
0.2
0.4
Inbreeding coefficient (middle third)

C.

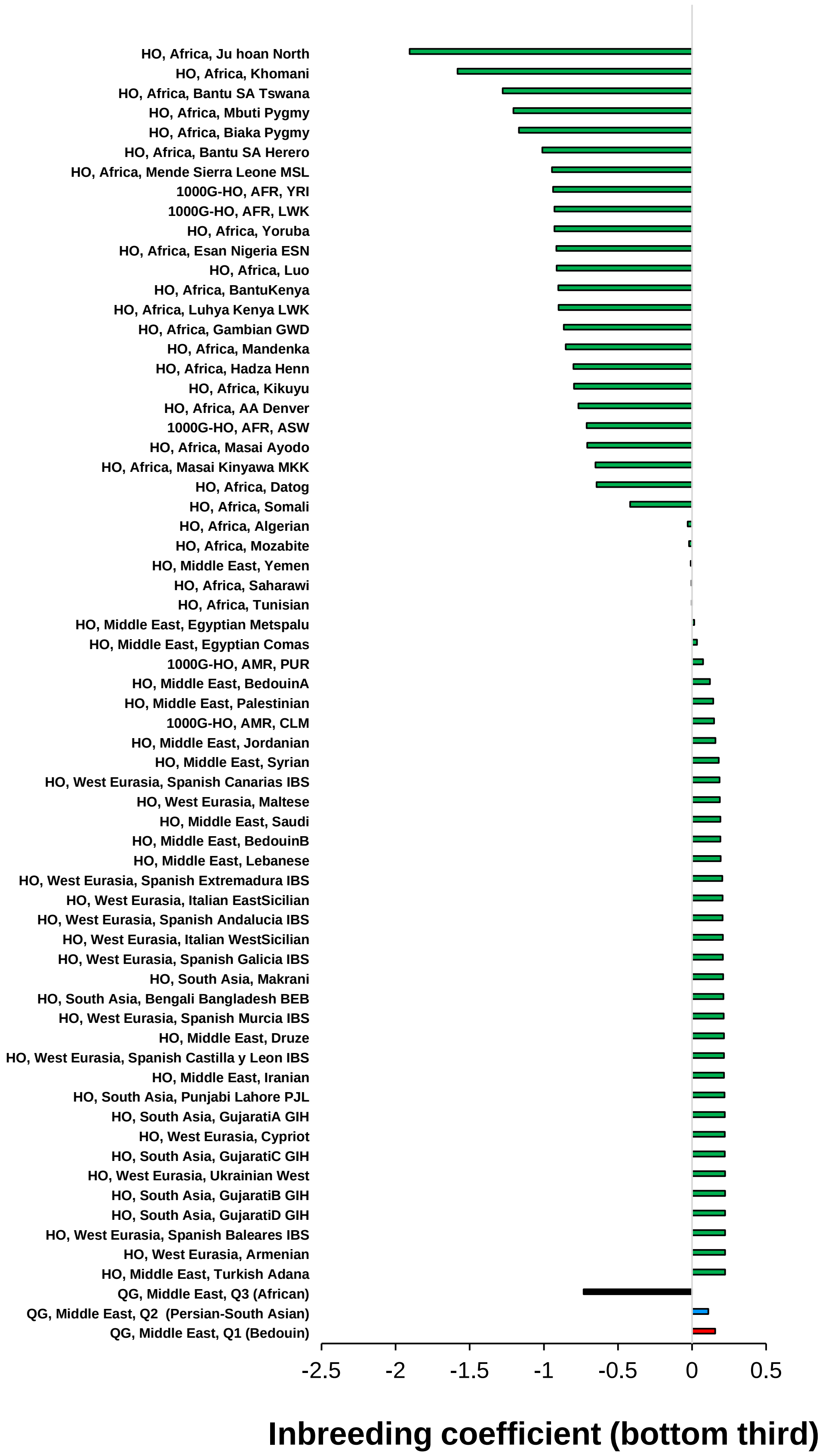
HO, Africa, Ju hoan North
HO, Africa, Khomani
HO, Africa, Bantu SA Tswana
HO, Africa, Mbuti Pygmy
HO, Africa, Biaka Pygmy
HO, Africa, Bantu SA Herero
HO, Africa, Mende Sierra Leone MSL
1000G-HO, AFR, YRI
1000G-HO, AFR, LWK
HO, Africa, Yoruba
HO, Africa, Esan Nigeria ESN
HO, Africa, Luo
HO, Africa, BantuKenya
HO, Africa, Luhya Kenya LWK
HO, Africa, Gambian GWD
HO, Africa, Mandenka
HO, Africa, Hadza Henn
HO, Africa, Kikuyu
HO, Africa, AA Denver
1000G-HO, AFR, ASW
HO, Africa, Masai Ayodo
HO, Africa, Masai Kinyawa MKK
HO, Africa, Datog
HO, Africa, Somali
HO, Africa, Algerian
HO, Africa, Mozabite
HO, Middle East, Yemen
HO, Africa, Saharawi
HO, Africa, Tunisian
HO, Middle East, Egyptian Metspalu
HO, Middle East, Egyptian Comas
1000G-HO, AMR, PUR
HO, Middle East, BedouinA
HO, Middle East, Palestinian
1000G-HO, AMR, CLM
HO, Middle East, Jordanian
HO, Middle East, Syrian
HO, West Eurasia, Spanish Canarias IBS
HO, West Eurasia, Maltese
HO, Middle East, Saudi
HO, Middle East, BedouinB
HO, Middle East, Lebanese
HO, West Eurasia, Spanish Extremadura IBS
HO, West Eurasia, Italian EastSicilian
HO, West Eurasia, Spanish Andalucia IBS
HO, West Eurasia, Italian WestSicilian
HO, West Eurasia, Spanish Galicia IBS
HO, South Asia, Makrani
HO, South Asia, Bengali Bangladesh BEB
HO, West Eurasia, Spanish Murcia IBS
HO, Middle East, Druze
HO, West Eurasia, Spanish Castilla y Leon IBS
HO, Middle East, Iranian
HO, South Asia, Punjabi Lahore PJL
HO, South Asia, GujaratiA GIH
HO, West Eurasia, Cypriot
HO, South Asia, GujaratiC GIH
HO, West Eurasia, Ukrainian West
HO, South Asia, GujaratiB GIH
HO, South Asia, GujaratiD GIH
HO, West Eurasia, Spanish Baleares IBS
HO, West Eurasia, Armenian
HO, Middle East, Turkish Adana
QG, Middle East, Q3 (African)
QG, Middle East, Q2 (Persian-South Asian)
QG, Middle East, Q1 (Bedouin)
-2.5
-2
-1.5
-1
-0.5
0
0.5
Inbreeding coefficient (bottom third)

Supplemental Figure 5

A.

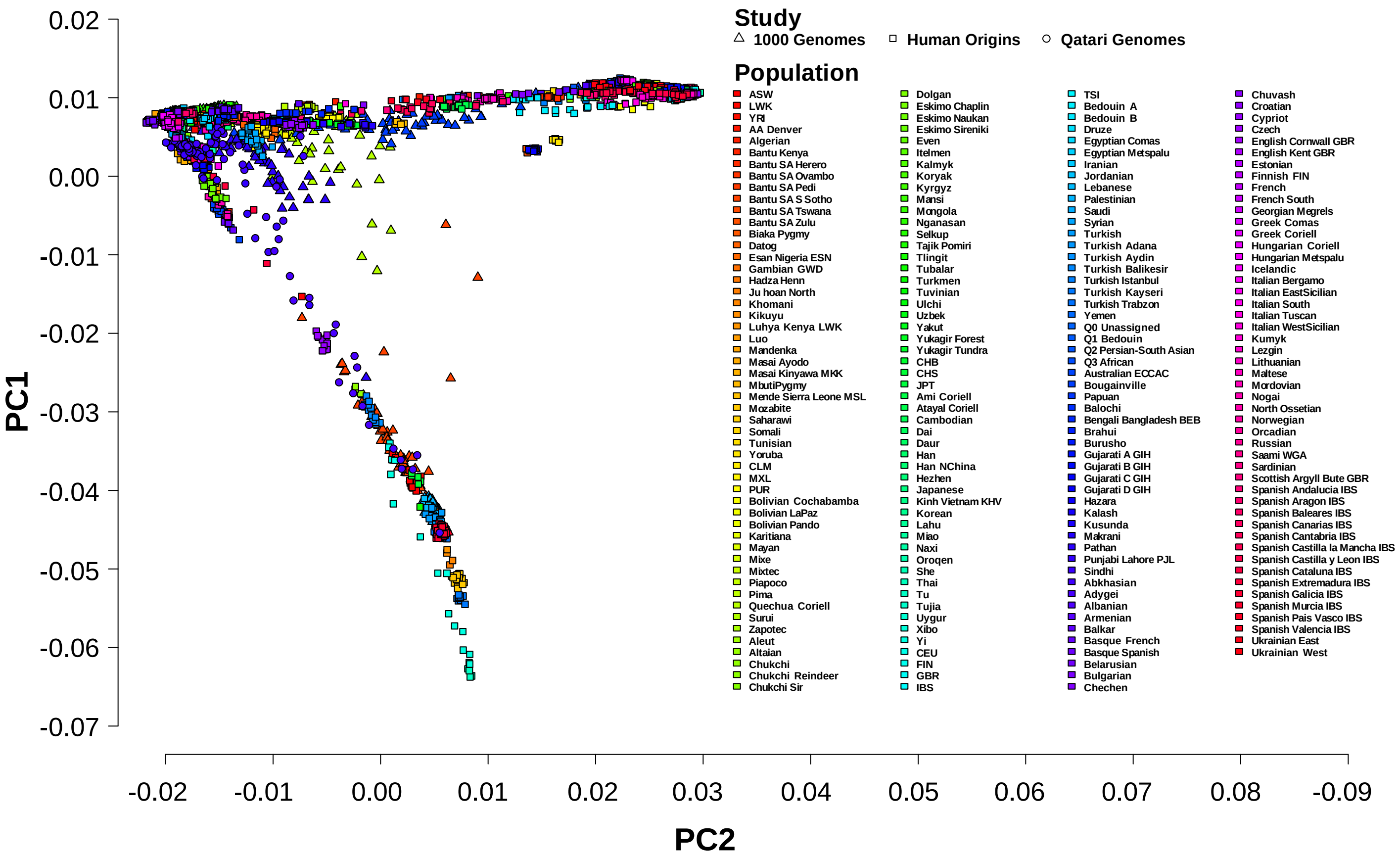

B.

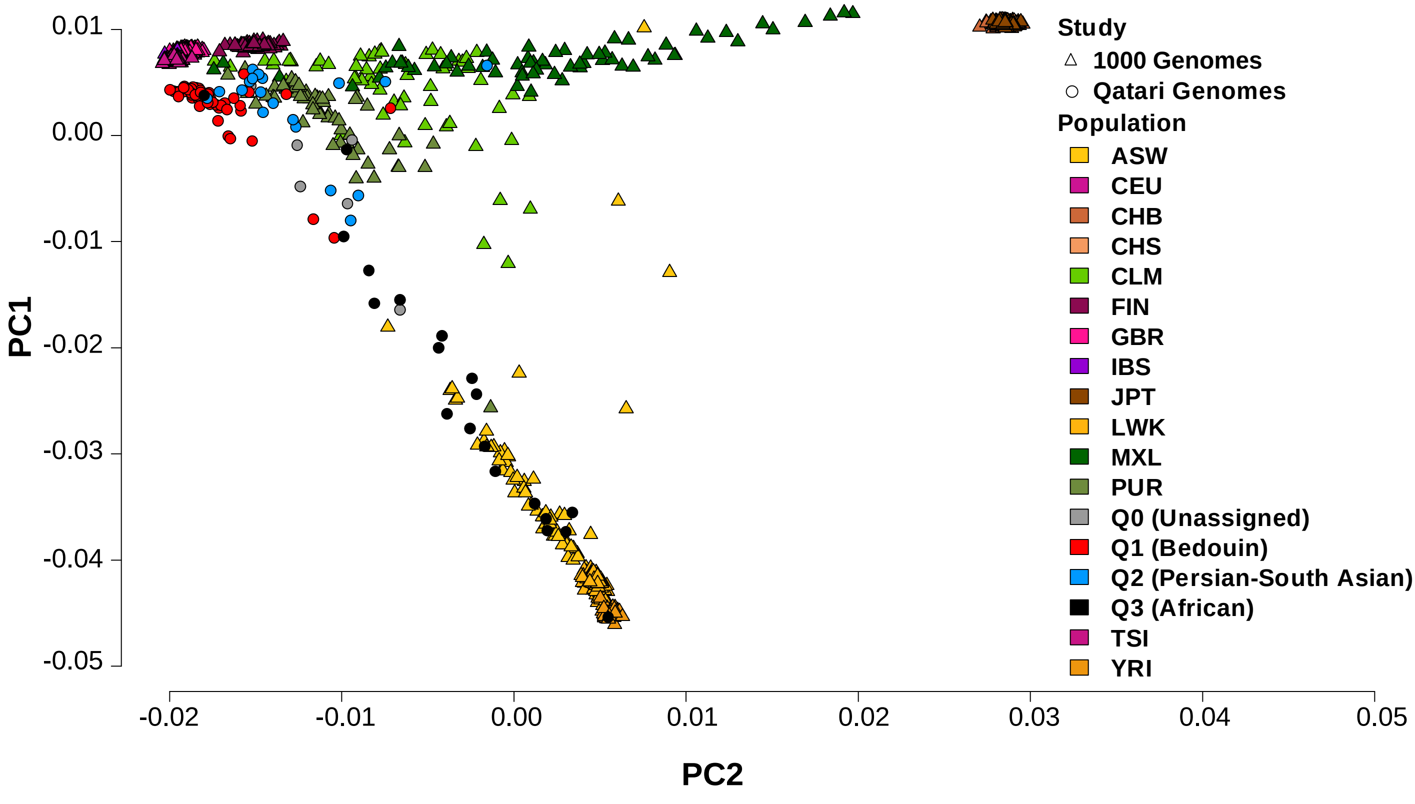

Supplemental Figure 6

Normalized nucleotide diversity in loci >0.18cM from genes

Genome-wide nongenic normalized nucleotide diversity

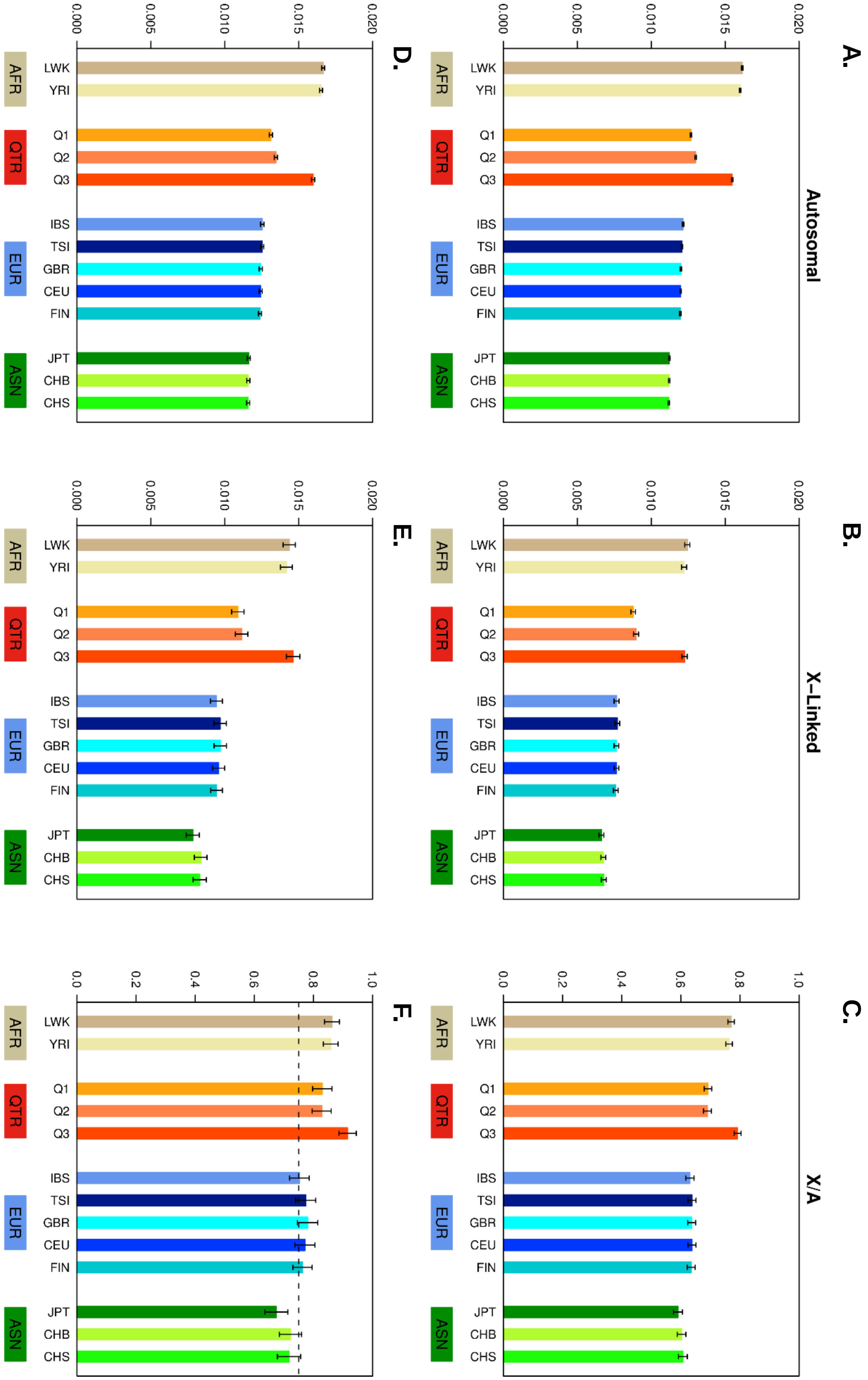

Supplemental Figure 7

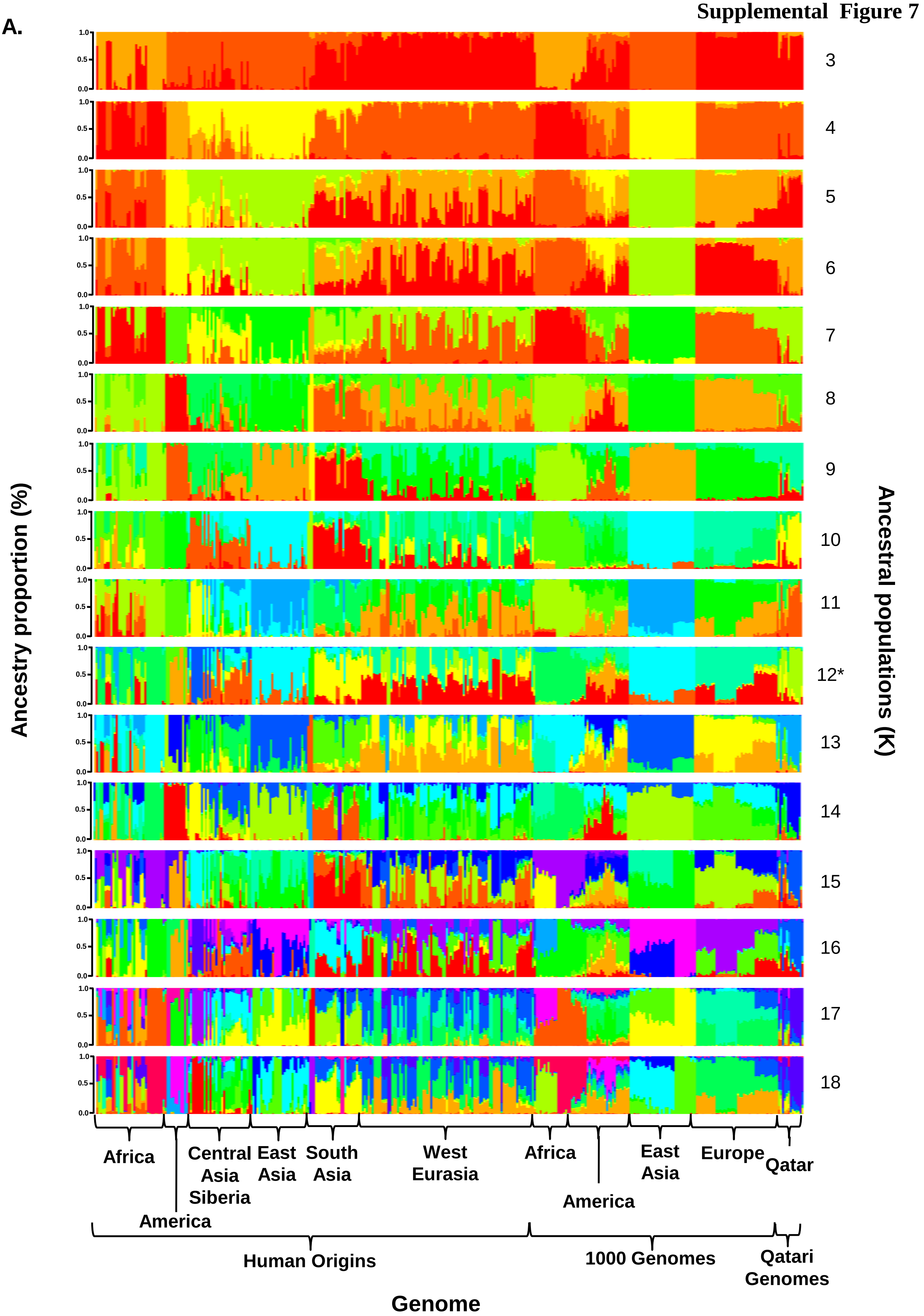

B.

Supplemental Figure 7

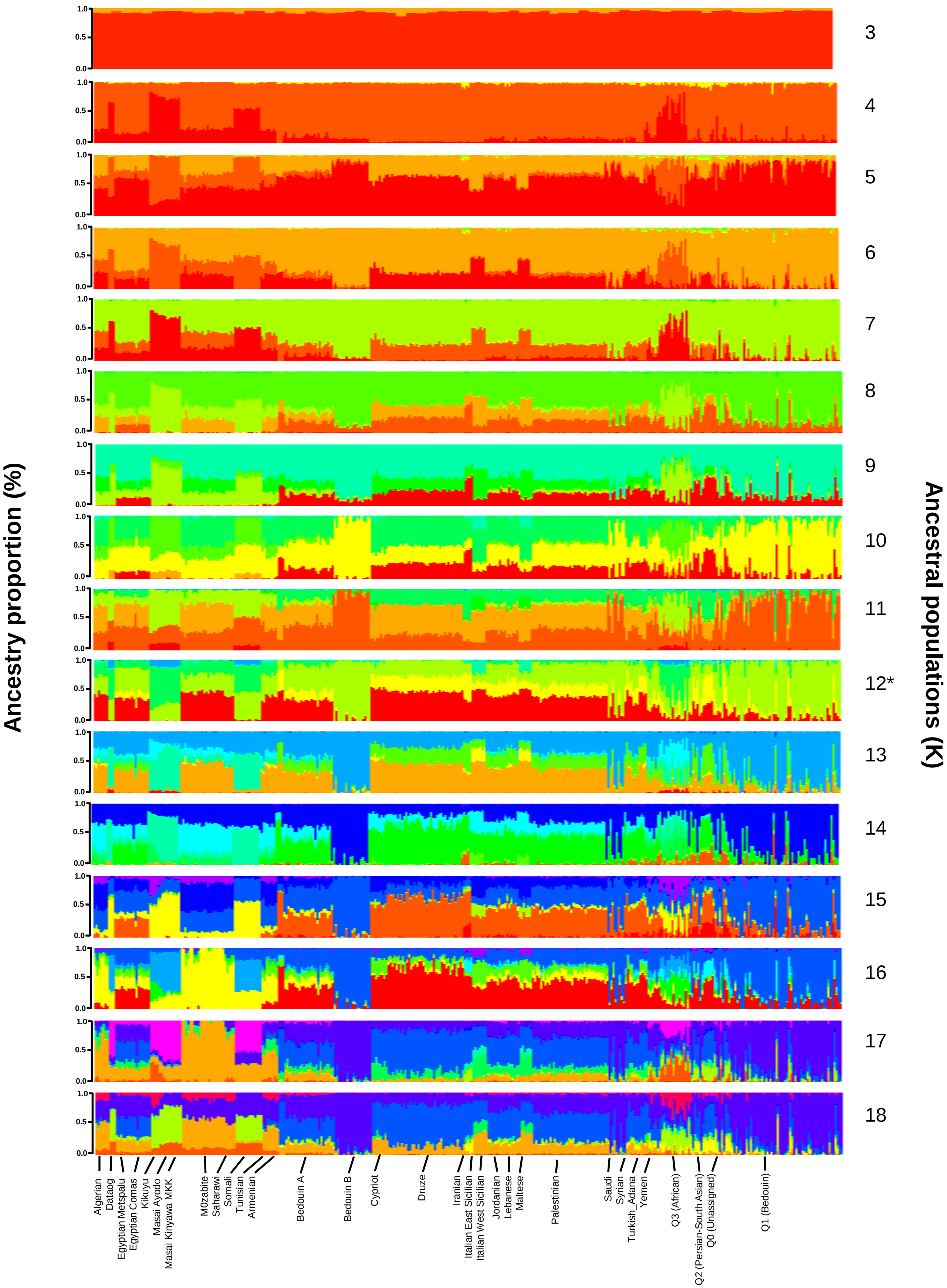

Middle Eastern Genome

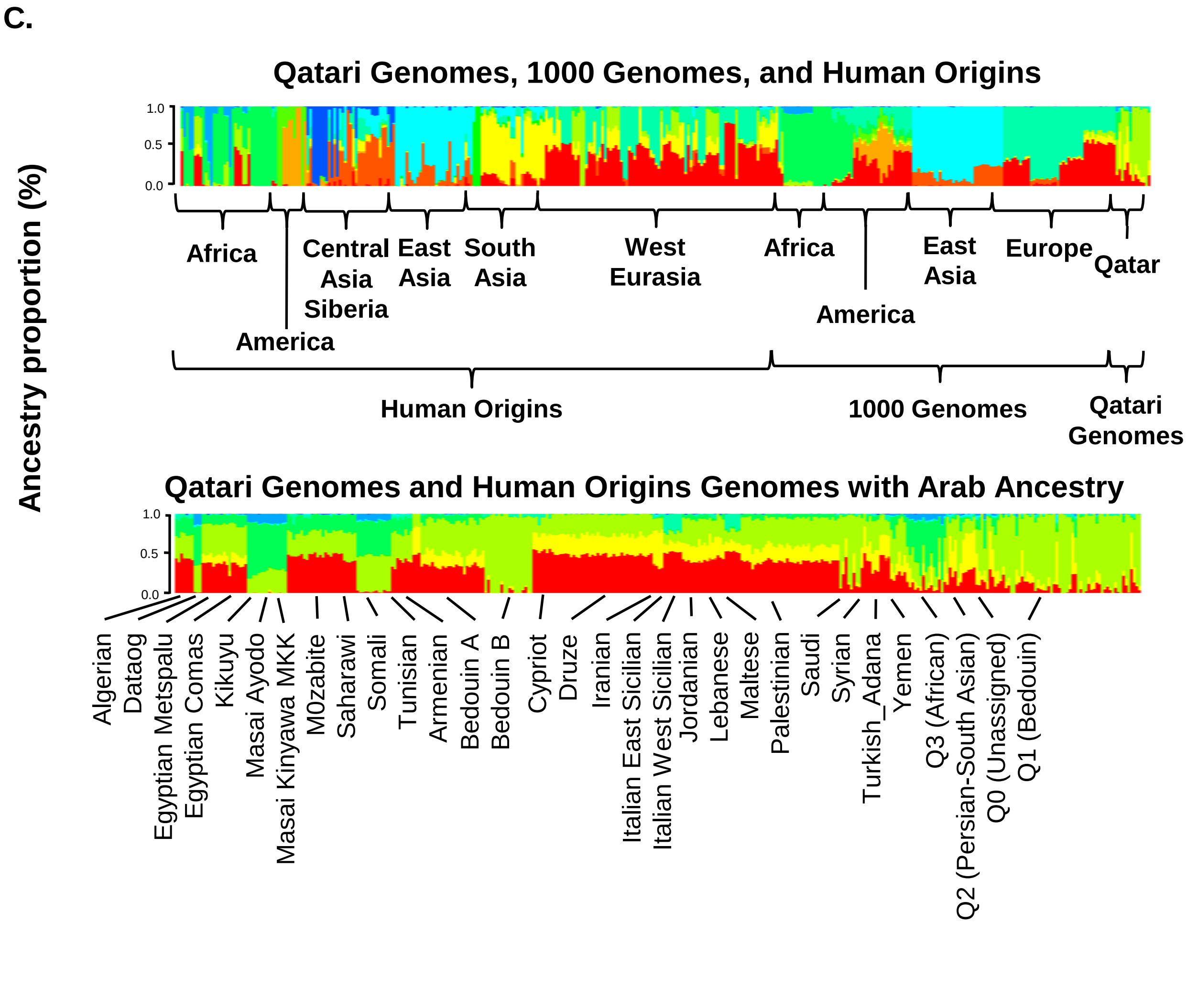
C.
Qatari Genomes, 1000 Genomes, and Human Origins
Ancestry proportion (%)
1.0
0.5
0.0
Africa
America
Central Asia Siberia
East Asia
South Asia
West Eurasia
Africa
America
East Asia
Europe
Qatar
Human Origins
1000 Genomes
Qatari Genomes
Qatari Genomes and Human Origins Genomes with Arab Ancestry
1.0
0.5
0.0
Algerian
Dataog
Egyptian Metspalu
Egyptian Comas
Kikuyu
Masai Ayodo
Masai Kinyawa MKK
M0zabite
Saharawi
Somali
Tunisian
Armenian
Bedouin A
Bedouin B
Cypriot
Druze
Iranian
Italian East Sicilian
Italian West Sicilian
Jordanian
Lebanese
Maltese
Palestinian
Saudi
Syrian
Turkish_Adana
Yemen
Q3 (African)
Q2 (Persian-South Asian)
Q0 (Unassigned)
Q1 (Bedouin)

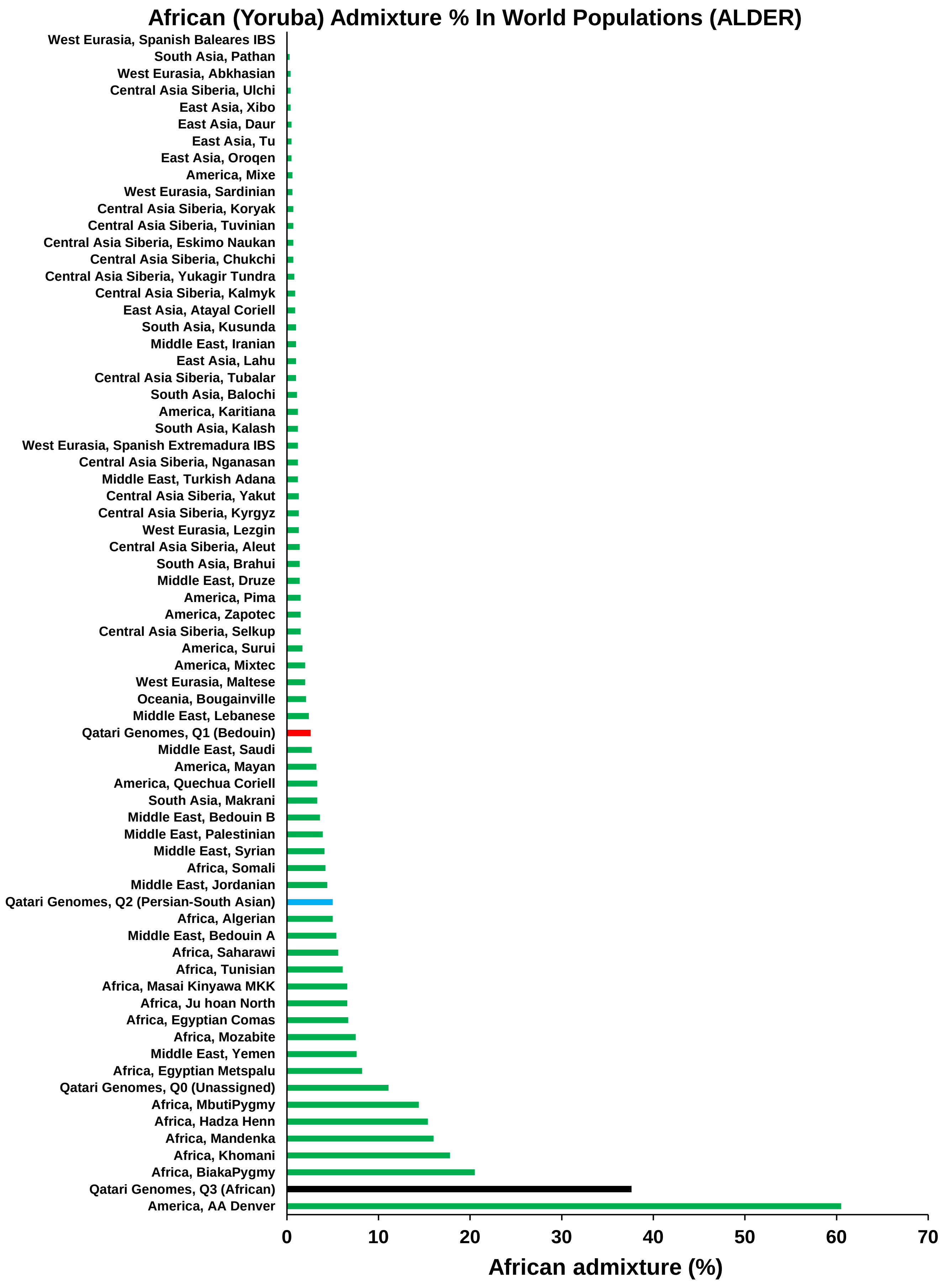
African (Yoruba) Admixture % In World Populations (ALDER)
West Eurasia, Spanish Baleares IBS
South Asia, Pathan
West Eurasia, Abkhasian
Central Asia Siberia, Ulchi
East Asia, Xibo
East Asia, Daur
East Asia, Tu
East Asia, Oroqen
America, Mixe
West Eurasia, Sardinian
Central Asia Siberia, Koryak
Central Asia Siberia, Tuvinian
Central Asia Siberia, Eskimo Naukan
Central Asia Siberia, Chukchi
Central Asia Siberia, Yukagir Tundra
Central Asia Siberia, Kalmyk
East Asia, Atayal Coriell
South Asia, Kusunda
Middle East, Iranian
East Asia, Lahu
Central Asia Siberia, Tubalar
South Asia, Balochi
America, Karitiana
South Asia, Kalash
West Eurasia, Spanish Extremadura IBS
Central Asia Siberia, Nganasan
Middle East, Turkish Adana
Central Asia Siberia, Yakut
Central Asia Siberia, Kyrgyz
West Eurasia, Lezgin
Central Asia Siberia, Aleut
South Asia, Brahui
Middle East, Druze
America, Pima
America, Zapotec
Central Asia Siberia, Selkup
America, Surui
America, Mixtec
West Eurasia, Maltese
Oceania, Bougainville
Middle East, Lebanese
Qatari Genomes, Q1 (Bedouin)
Middle East, Saudi
America, Mayan
America, Quechua Coriell
South Asia, Makrani
Middle East, Bedouin B
Middle East, Palestinian
Middle East, Syrian
Africa, Somali
Middle East, Jordanian
Qatari Genomes, Q2 (Persian-South Asian)
Africa, Algerian
Middle East, Bedouin A
Africa, Saharawi
Africa, Tunisian
Africa, Masai Kinyawa MKK
Africa, Ju hoan North
Africa, Egyptian Comas
Africa, Mozabite
Middle East, Yemen
Africa, Egyptian Metspalu
Qatari Genomes, Q0 (Unassigned)
Africa, MbutiPygmy
Africa, Hadza Henn
Africa, Mandenka
Africa, Khomani
Africa, BiakaPygmy
Qatari Genomes, Q3 (African)
America, AA Denver
0
10
20
30
40
50
60
70
African admixture (%)

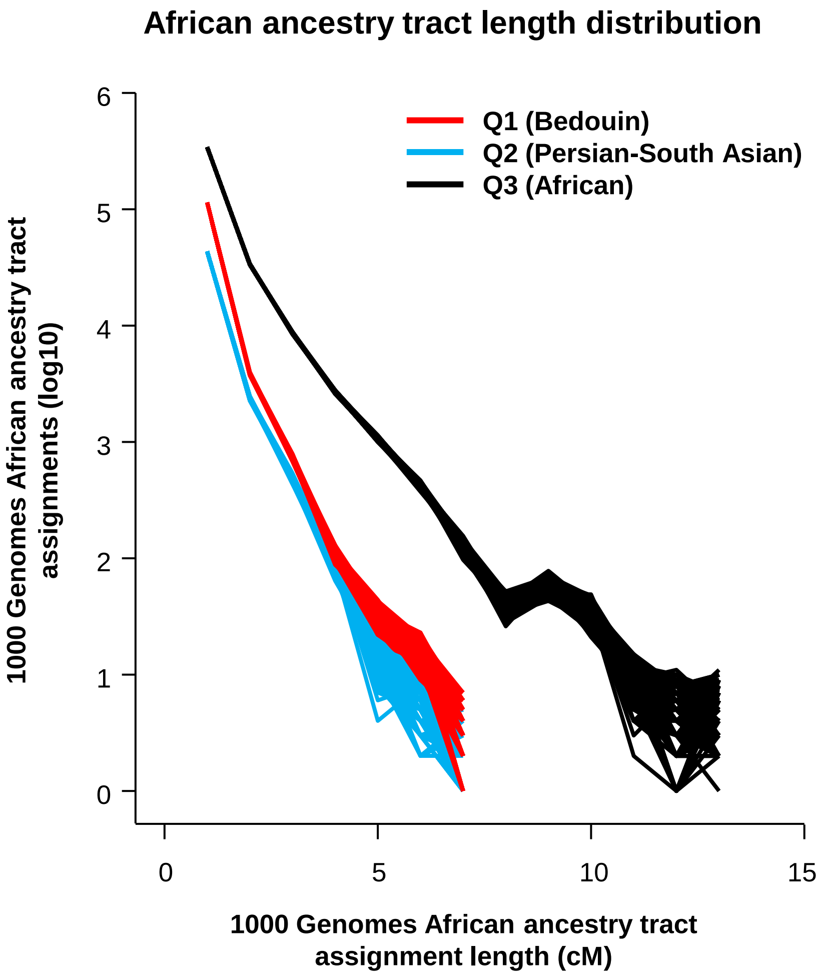
African ancestry tract length distribution
Q1 (Bedouin)
Q2 (Persian-South Asian)
Q3 (African)
1000 Genomes African ancestry tract assignments (log10)
1000 Genomes African ancestry tract assignment length (cM)
0
1
2
3
4
5
6
0
5
10
15

Supplemental Figure 10

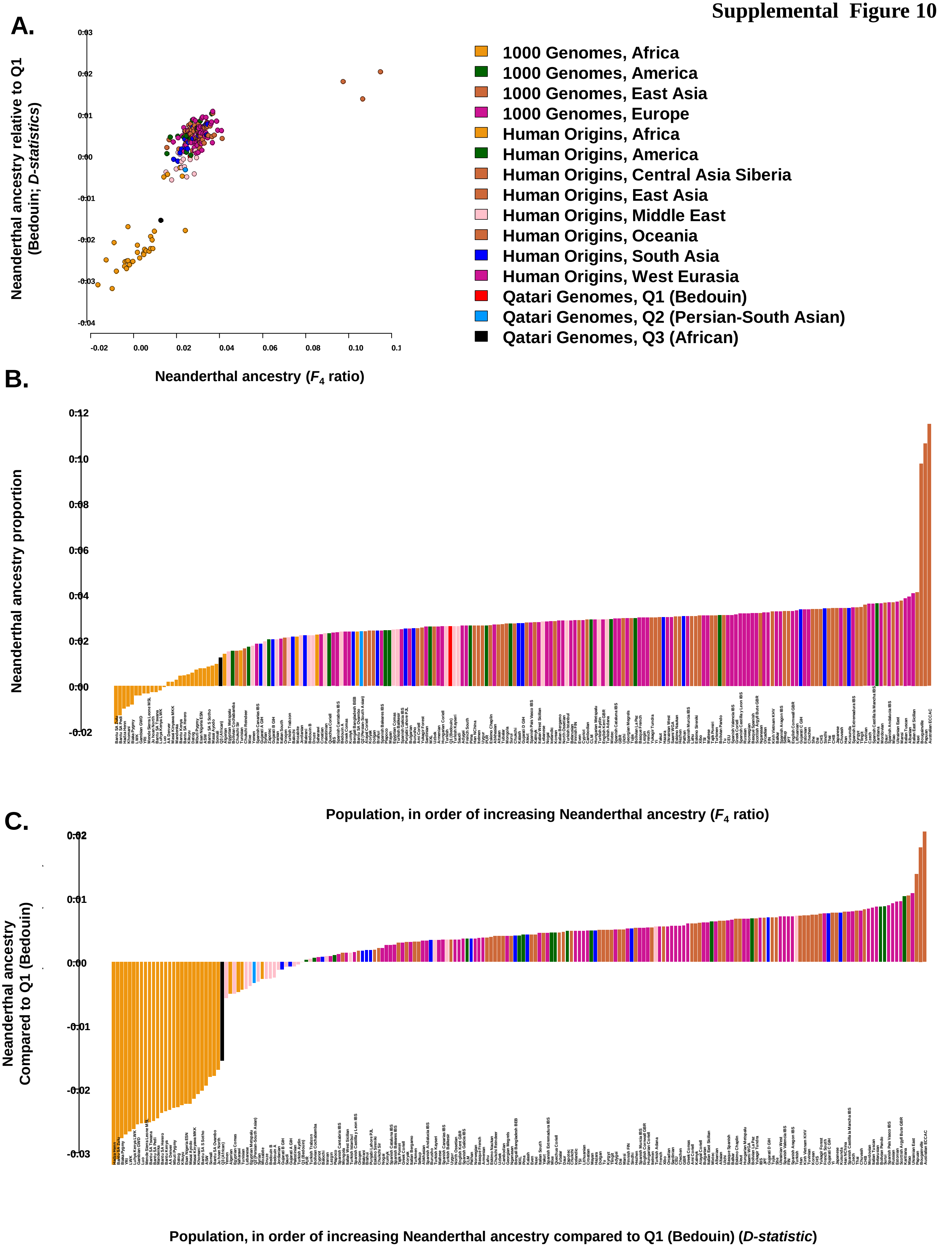

Supplemental Figure 11

A. TreeMix with 0 migration edges

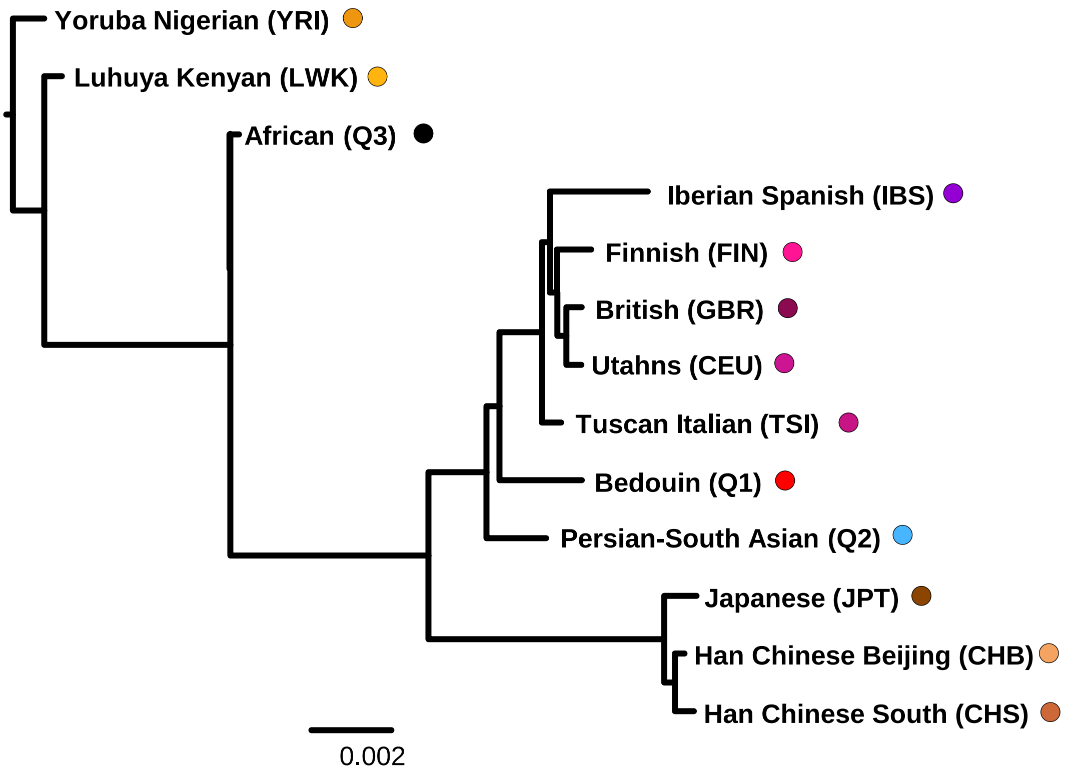

B. TreeMix with 1 migration edges

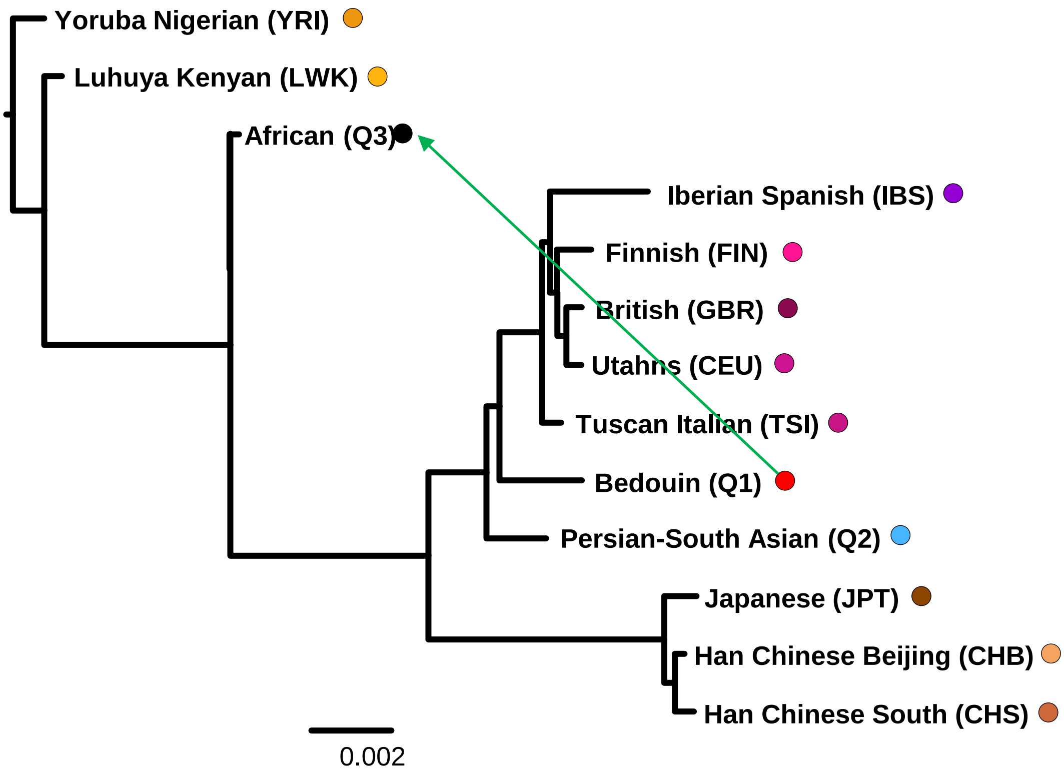

C. TreeMix with 2 migration edges

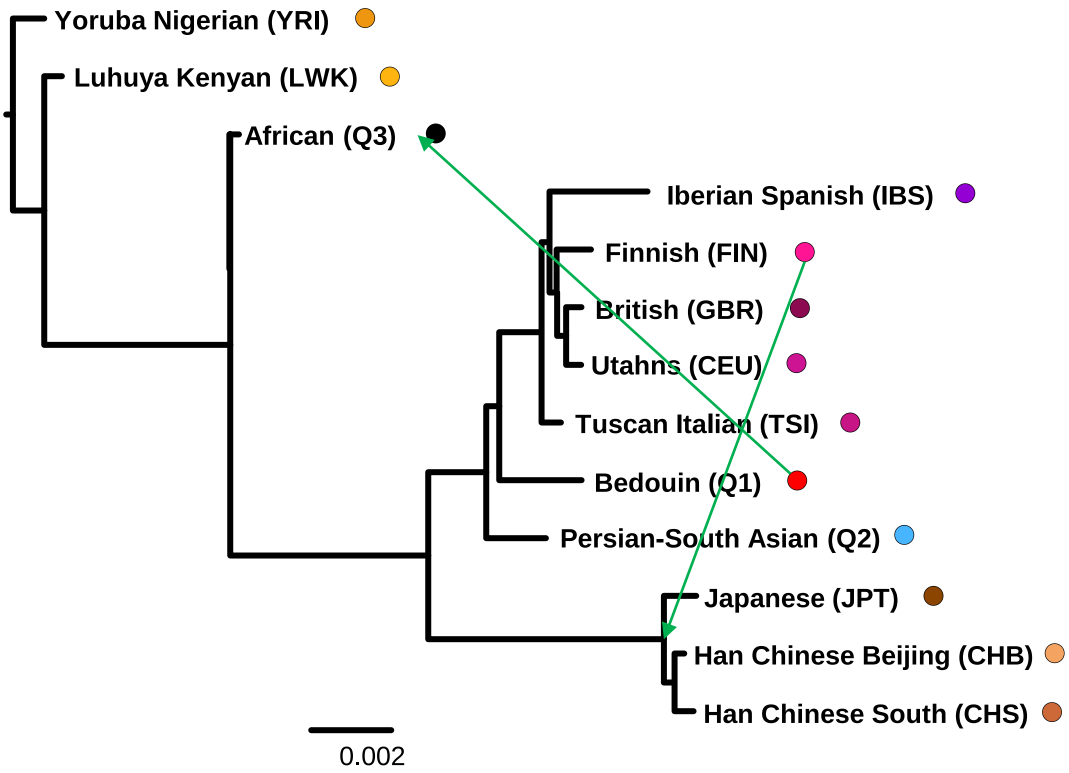

D. TreeMix with 3 migration edges

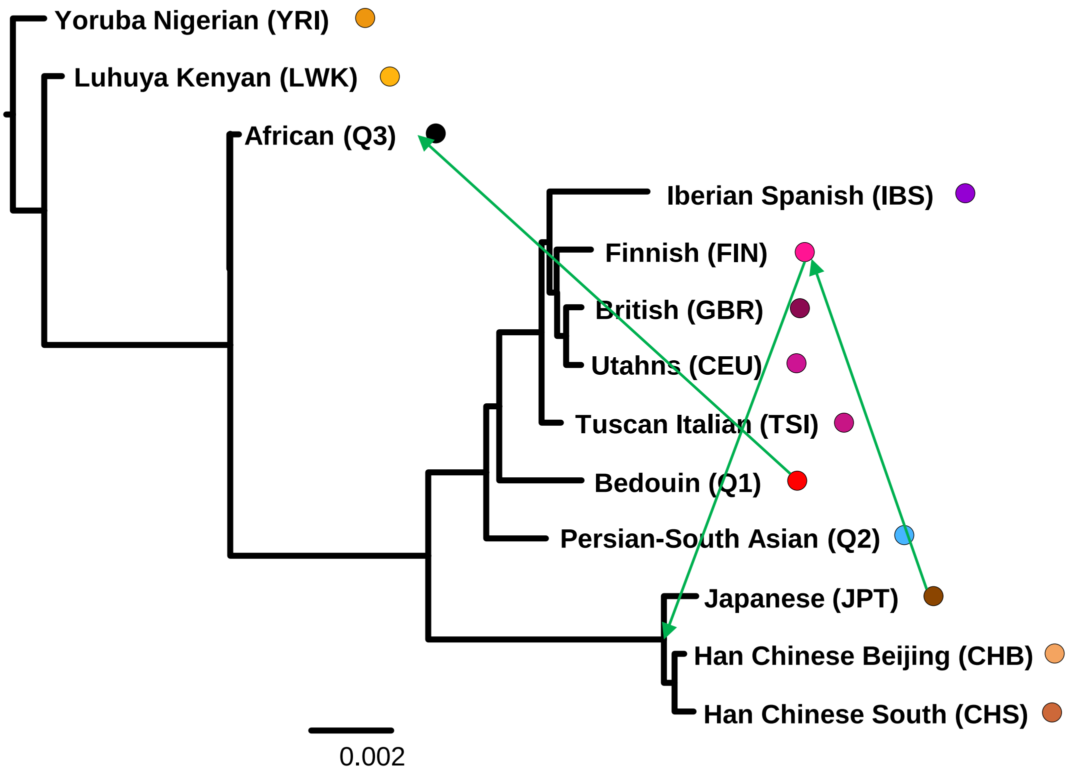

E. TreeMix with 4 migration edges

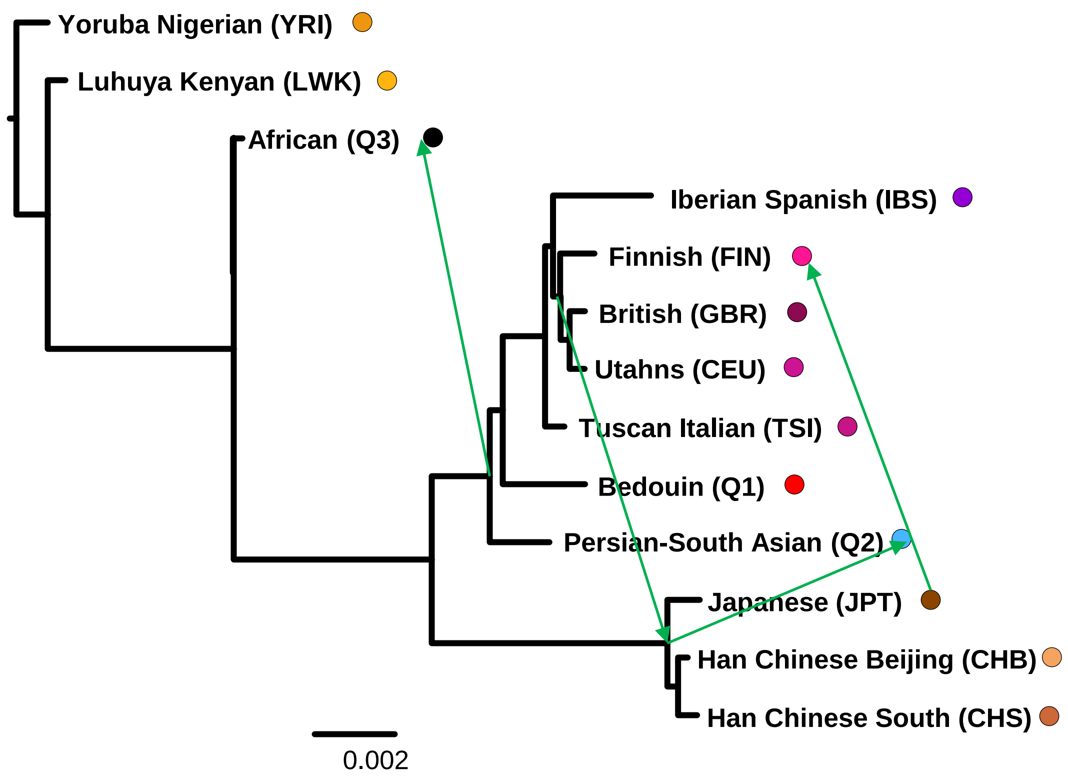

F. TreeMix with 5 migration edges

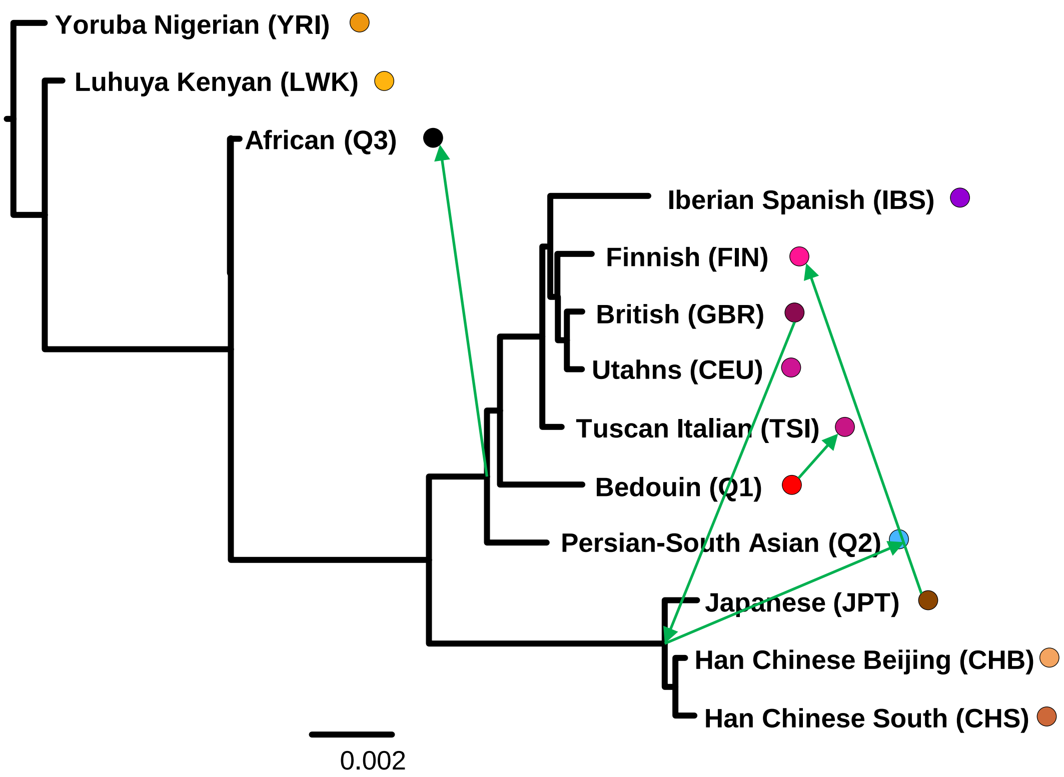

G. TreeMix residuals with 0 migration edges

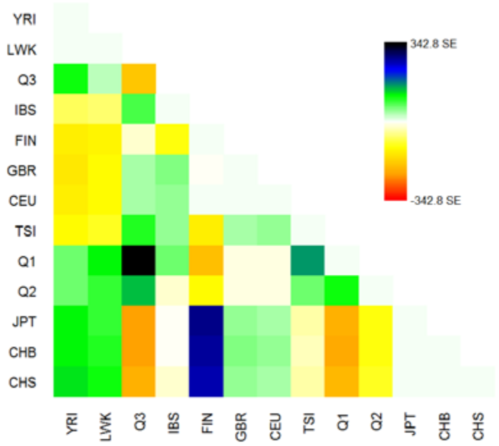

H. TreeMix residuals with 1 migration edge

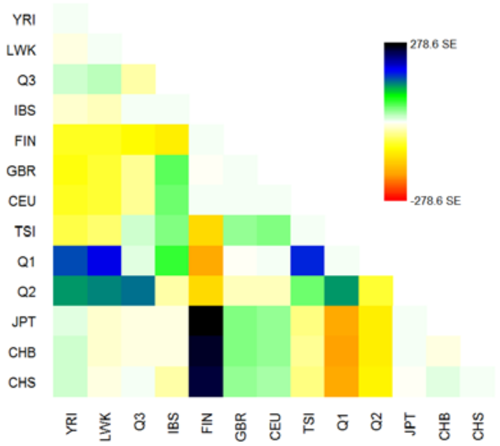

I. TreeMix with 2 migration edges

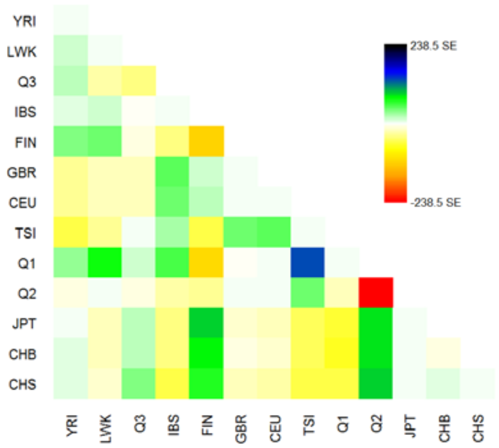

J. TreeMix with 3 migration edges

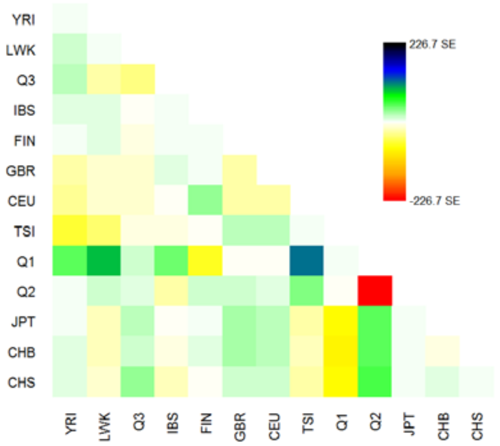

K. TreeMix with 4 migration edges

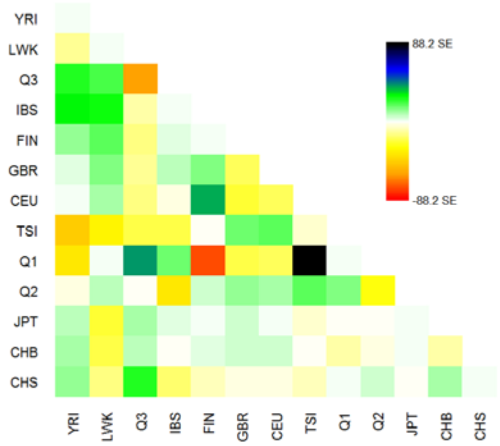

L. TreeMix with 5 migration edges

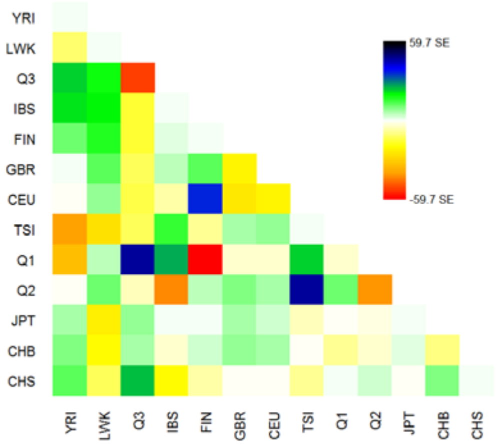

Supplement: Supplemental Material [file supp_gr.191478.115_Supplemental_Data.pdf]
